# Synthesis of fluorinated acid-functionalized, electron-rich nickel porphyrins

**DOI:** 10.3762/bjoc.20.248

**Published:** 2024-11-15

**Authors:** Mike Brockmann, Jonas Lobbel, Lara Unterriker, Rainer Herges

**Affiliations:** 1 Otto Diels-Institute of Organic Chemistry, Christian-Albrechts-Universität zu Kiel, Otto-Hahn-Platz 4, 24118 Kiel, Germany

**Keywords:** acid-functionalized porphyrin, electron-rich porphyrin, nickel porphyrin, perfluorinated aliphatic carboxylic acids, porphyrin synthesis

## Abstract

In this study, novel fluorinated carboxylic acid esters of the generic structure TfO–CH_2_–(CF_2_)*_n_*–COOCH_3_ (*n* = 2,4,6, Tf = triflate) were synthesized. The triflates were reacted with 2-hydroxy-3,4,5-trimethoxybenzaldehyde via Williamson ether syntheses. The resulting electron-rich compounds were used as aldehydes in the Rothemund reaction with pyrrole to form ester-substituted porphyrins. After metalation with Ni(acac)_2_ and hydrolysis electron-rich porphyrins were obtained, that are equipped with covalently attached long chain acid substituents. The target compounds have potential applications in catalysis, sensing, and materials science. The fluorinated aliphatic carboxylic acids (TfO–CH_2_–(CF_2_)*_n_*–COOCH_3_) with triflate as leaving group in terminal position are easily accessible and versatile building blocks for attaching long chain acids (p*K*_a_ 0–1) to substrates in Williamson ether-type reactions.

## Introduction

Metal porphyrins are prosthetic groups in a number of essential biomolecules, including hemoglobin, chlorophyll, and cytochromes, supporting processes such as oxygen transport, photosynthesis, and electron transfer [1–5]. Beyond their essential biological roles, porphyrins and their derivatives are employed in a number of applications, acting as catalysts in numerous reactions, including oxidation, reduction, and cycloaddition [6–10]. Particularly when electron-rich porphyrins act as reducing agents, e.g. in electrocatalytic hydrogen evolution reactions, a proton source is needed [11]. In this context, trifluoroacetic acid is very frequently chosen as the proton source, because it is a strong acid but just not strong enough to destroy (demetallate) the Ni porphyrin [10]. Covalent attachment of acids facilitates proton transfer and increases the efficiency [12]. Three conditions should be met for the target porphyrins of this study. 1. The acid covalently bound to the porphyrin should have an acid strength similar to trifluoroacetic acid. 2. The length of the tether with which the acid group is bound should be sufficient to serve as a proton source for redox reactions at the metal. 3. The electronic properties of the porphyrin, especially the low oxidation potential, should not be increased. We have chosen four-fold meso-3,4,5-trimethoxyphenyl-substituted Ni porphyrin as the electron-rich system, however, the post-synthetic modification of this porphyrin proved to be difficult. Therefore, we have integrated the acid group into the aldehyde component of the Rothemund reaction to prepare the target porphyrin. In initial tests, we have established that trifluoroacetic acid can be replaced by perfluorinated alkyl carboxylic acids [10]. It was therefore obvious to use a perfluoroalkyl chain as a tether. However, a perfluoroalkyl chain as a substituent on the porphyrin has an electron-withdrawing effect and thus a negative influence on the oxidation potential. We have therefore inserted an O–CH_2_ group between the phenyl group of the porphyrin and the perfluoroalkyl chain. The oxygen atom, especially in the 2-position, should even improve the electronic properties of the porphyrin.

## Results and Discussion

**Scheme 1:**
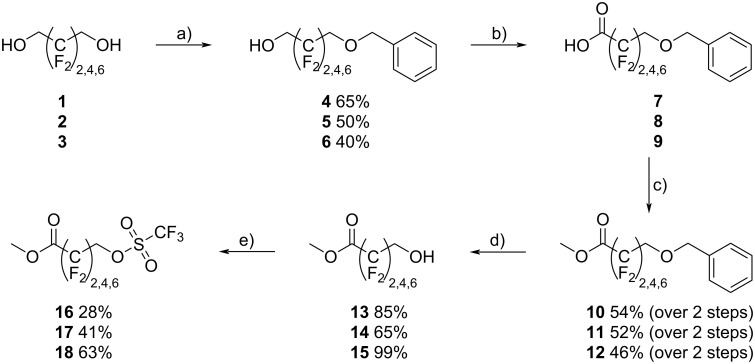
Synthesis of the starting materials **16**, **17**, and **18** for the subsequent Williamson ether synthesis with 2-hydroxy-3,4,5-trimethoxybenzaldehyde (**21**). Conditions: a) K_2_CO_3_, benzyl bromide, abs. MeCN, N_2_, reflux, 18 h; b) TEMPO, KBr, NaOCl, NaHCO_3_, MeCN, rt, 76 h; c) MeOH, H_2_SO_4_, reflux, 18 h; d) Pd/C, H_2_, EtOH, rt, 24 h; e) Tf_2_O, pyridine, DCM, rt, 18 h.

Our synthesis started with the readily available fluorinated symmetric diols HO–CH_2_–(CF_2_)*_n_*–CH_2_–OH (*n* = 2,4,6, see Scheme 1).

In order to break the symmetry and to generate the acid function only on one side, benzyl protection was performed. From diols **1**, **2**, and **3** statistical mixtures of unprotected, mono-, and di-protected products were obtained, from which the isolation of the desired mono-protected products **4** (65%), **5** (50%), and **6** (40%) by chromatography was straightforward. However, the subsequent oxidation of the alcohol with the usual oxidizing agents (Jones reagent, KMnO_4_, etc.) was not successful. A radical oxidation with TEMPO, potassium bromide (KBr), sodium hypochlorite (NaOCl), and sodium bicarbonate (NaHCO₃) provided acids **7**, **8**, and **9**. A byproduct is obtained during oxidation and it is assumed that this is the molecule oxidized at the benzyl position (see Supporting Information File 1, compounds **35**–**40**). Work-up and isolation proved to be difficult, and therefore, the acids were directly converted into the methyl esters **10** (54%), **11** (52%), and **12** (46%). The benzyl-protecting group was removed hydrogenolytically to give products **13** (85%), **14** (65%), and **15** (99%). The alcohols were then converted to the triflates **16** (28%), **17** (41%), and **18** (63%).

**Scheme 2:**
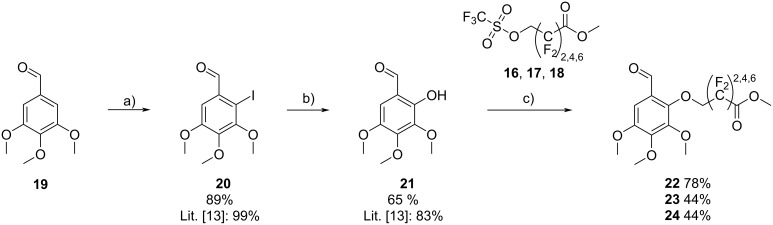
Synthesis of perfluoroalkyl ester-functionalized aldehydes **22**, **23**, and **24**. Conditions: a) NIS, TFA, Na_2_CO_3_, MeCN, reflux, 18 h; b) Cu_2_O·H_2_O, 2-pyridinaldoxime, TBAB, CsOH, H_2_O, N_2_, rt, 18 h; c) Cs_2_CO_3_, DMAc, N_2_, rt, 3 h.

We have chosen 3,4,5-trimethoxybenzaldehyde (**19**) as the aldehyde component due to its commercial availability. A OH group was introduced to serve as the nucleophile in the Williamson ether synthesis with the triflates **16**, **17**, and **18** (Scheme 2).

Towards this end, 3,4,5-trimethoxybenzaldehyde (**19**) was iodinated using *N*-iodosuccinimide (NIS) to give **20** in a yield of 89% [13]. To convert the iodo to an OH group, compound **20** was reacted with Cu_2_O, 2-pyridinaldoxime and CsOH to give 2-hydroxy-3,4,5-trimethoxybenzaldehyde (**21**, 65%) [13]. In a subsequent nucleophilic substitution, the fluorinated alkyl chains of **16**, **17**, and **18** were linked via a Williamson ether synthesis to yield **22** (78%), **23** (44%), and **24** (44%).

**Scheme 3:**
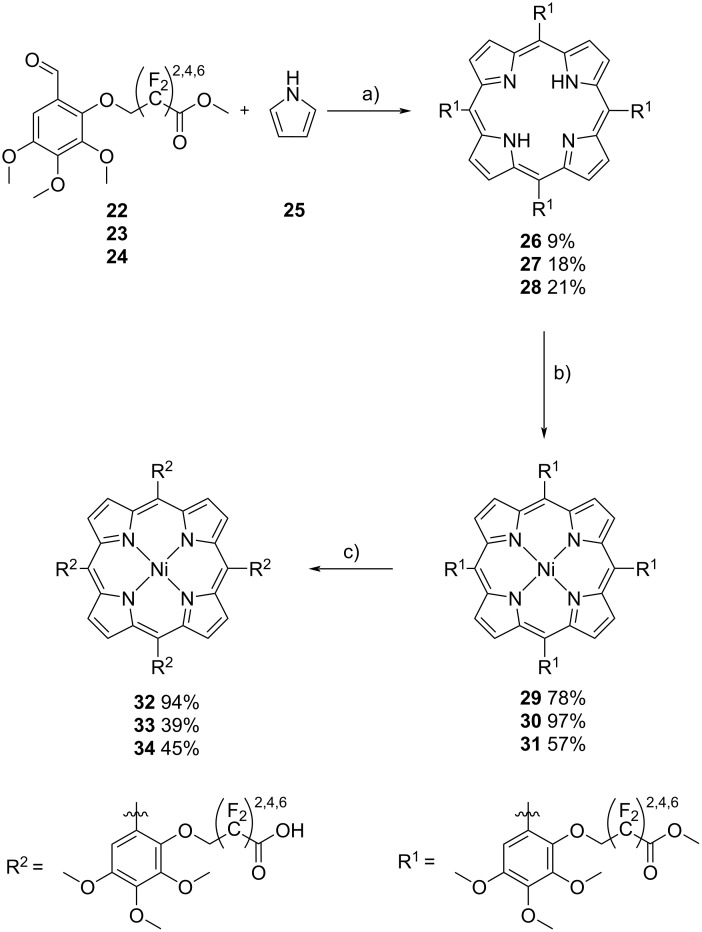
Porphyrin synthesis. a) Rothemund porphyrin synthesis of metal-free porphyrins **26**, **27**, and **28**; b) metalation of porphyrins with Ni(acac)_2_; c) ester hydrolysis to generate the free acids **32**, **33**, and **34**. Conditions: a) 1) **22**/**23**/**24**, TFA, abs. DCM, N_2_, reflux, 30 min, 2) pyrrole, reflux, 2.5 h, 3) DDQ, reflux, 2 h; b) Ni(acac)_2_, toluene, reflux, 20 h; c) 1) LiOH, MeOH, rt, 1 h, 2) HCl.

Compounds **22**, **23**, and **24** were used as aldehyde components in the Rothemund-type synthesis of metal-free porphyrins **26** (9%), **27** (18%), and **28** (21%) (see Scheme 3).

Metalation was achieved with nickel acetylacetonate to obtain the ester-substituted Ni porphyrins **29** (78%), **30** (97%), and **31** (57%). The latter were treated with LiOH and HCl to give the free acids **32** (94%), **33** (39%), and **34** (45%). The HPLC–ESIMS analysis of **32**, **33**, and **34** revealed that two major atropisomers of each porphyrin had formed. In **32** both atropisomers exhibit a roughly 1:1 ratio, in **33** we observed a roughly 1:2 ratio, and in **34** almost only one atropisomer was formed (see Figures S102, S106, and S110 in Supporting Information File 1). We attribute this to the increasing sterical hindrance of the increasing chain lengths in compounds **32**, **33**, and **34**, which should favor an alternating sequence of the chains pointing upward and downward.

## Conclusion

This study reports the synthesis of perfluoroalkyl carboxylic esters with CH_2_–OTf groups in the ω-position of the type TfO–CH_2_–(CF_2_)*_n_*–COOCH_3_ (*n* = 2, 4, 6, Tf = triflate). The latter compounds were used in Williamson ether reactions with 2-hydroxy-3,4,5-trimethoxybenzaldehyde (**21**) to prepare the aldehyde component for a Rothemund-type porphyrin synthesis of acid-functionalized electron-rich porphyrins. The corresponding Ni porphyrins are potential compounds for electrocatalysis and sensor applications. The ω-triflated, perfluoroalkylated carboxylic acids **16**, **17**, and **18** are easily accessible and versatile building blocks for connecting long chain acids (p*K*_a_ range between 0 and 1) to substrates in Williamson ether-type reactions.

## Supporting Information

File 1 Experimental procedures, characterization data of all products, and copies of ^1^H, ^13^C, and ^19^F NMR spectra.

## Data Availability

All data that supports the findings of this study is available in the published article and/or the supporting information of this article.
