# Supplementary material for: Synthesis of fluorinated acid-functionalized, electron-rich nickel porphyrins
Source: Beilstein J Org Chem. 2024 Nov 15;20:2954–8. doi: 10.3762/bjoc.20.248 (PMC11572008; doi:10.3762/bjoc.20.248)
Supplement: File 1 — Experimental procedures, characterization data of all products, and copies of 1H, 13C, and 19F NMR spectra. [file Beilstein_J_Org_Chem-20-2954-s001.pdf]

BEILSTEIN JOURNAL OF ORGANIC CHEMISTRY

Supporting Information

for

Synthesis of fluorinated acid-functionalized, electron-rich nickel porphyrins

Mike Brockmann, Jonas Lobbel, Lara Unterriker and Rainer Herges

Beilstein J. Org. Chem. **2024,** *20,* 2954–2958. doi:10.3762/bjoc.20.248

Experimental procedures, characterization data of all products, and copies of ^{1}H, ^{13}C, and ^{19}F NMR spectra

License and Terms: This is a supporting information file under the terms of the Creative Commons Attribution License (https://creativecommons.org/licenses/by/4.0). Please note that the reuse, redistribution and reproduction in particular requires that the author(s) and source are credited and that individual graphics may be subject to special legal provisions.
The license is subject to the *Beilstein Journal of Organic Chemistry* terms and conditions: (https://www.beilstein-journals.org/bjoc/terms)

Table of contents

1. Mass spectrometry....S2
2. NMR spectroscopy....S2
3 Synthesis....S4
3.1 General procedure for benzyation of **1–3**....S4
3.2 General procedure for oxidation of **4–6**....S12
3.3 General procedure for the esterification of **7**, **8** and **9**....S22
3.5 General procedure for the triflation of **13**, **14** and **15**....S44
3.6 Synthesis of 2-Iodo-3,4,5-trimethoxybenzaldehyde (**20**)....S53
3.7 Synthesis of 2-Hydroxy-3,4,5-trimethoxybenzaldehyde (**21**)....S56
3.8 General procedure for the ethers **22**, **23** and **24**....S58
3.9 General procedure for the metal-free porphyrins **26**, **27** and **28**....S68
3.10 General procedure for the nickel porphyrins **29**, **30** and **31**....S76
3.11 General procedure of nickel porphyrins **32**, **33** and **34** (free acid)....S84
References....S100

Experimental section

1. Mass spectrometry

Electron ionization mass spectra were measured at 70 eV ionization energy using a Jeol Accu-TOF 4G mass spectrometer (EI). Electrospray ionization mass spectra (ESI–MS) were recorded using a Finnigan LCQ Deca quadrupole ion trap mass spectrometer. The separation with HPLC was carried out using a Thermo Scientific Ultimate 3000 UHPLC device coupled with the aforementioned ESI–MS. The Zorbax RRHD Rapid Resolution High Definition (1.8 µm) column from Agilent Technologies was used.

2. NMR spectroscopy

All NMR spectra were recorded in deuterated solvents from Deutero. Two-dimensional NMR techniques were used to assign the signals (COSY, HSQC, HMBC). The following table indicates both the degree of deuteration of each solvent and the solvent signal. The ^{1}H and ^{13}C NMR spectra were referenced to remaining non-deuterated or partially deuterated solvents. ^{19}F NMR spectra were externally referenced to CCl_3F. The ^{13}C NMR spectra and the ^{19}F NMR spectra were measured with hydrogen decoupling.

solvent	deuteration level	^{1}H-signal / ppm	^{13}C-signal / ppm
$CDCl_3$	99.8 %	7.26 (singlet)	77.16 (triplet)

The following spectrometers were used:

device name	frequency / MHz		
	^{1}H NMR spectra	^{13}C NMR spectra	^{19}F NMR spectra
Bruker DRX 500	500	125	470
Bruker AV 600	600	150	

For the evaluation of the NMR spectra of the porphyrins, the carbon atoms were numbered as follows:

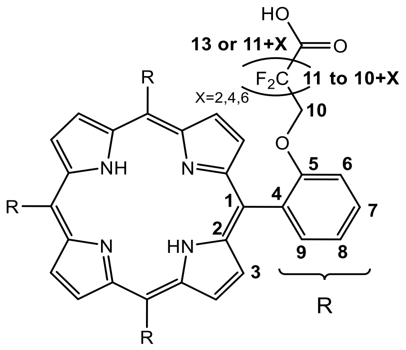

Starting from the *meso* position of the porphyrin, the porphyrin was numbered consecutively. The substituent at the *meso* position of the porphyrin follows the scheme above. The symmetry of the molecules was considered in the numbering.

Some carbon atoms of certain porphyrins could be assigned via the heteronuclear single quantum coherence (HSQC) and the heteronuclear multiple bond correlation (HMBC) spectra. Since the porphyrins are mixtures of atropisomers assignment of the signal was not possible in all cases.

3 Synthesis

The syntheses of the fluorinated alkyl chains of different lengths are given as general procedures.

3.1 General procedure for benzylidation of 1–3

Under a nitrogen atmosphere 1 equiv of the corresponding diol (**1**–**3**) was mixed with potassium carbonate (2 equiv). Then, abs. acetonitrile (100 mL) and benzyl bromide (1 equiv) were added and stirred for 18 h under reflux. Afterwards, the solution was filtered over Celite® 545 and the solvent was removed in vacuo. The crude product was then purified by flash column chromatography (cyclohexane/ethyl acetate, gradient 5%–60% ethyl acetate). A colorless oil was obtained.

4-Benzyloxy-2,2,3,3-tetrafluorobutan-1-ol (4)

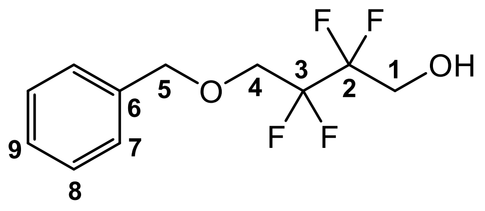

Yield: 65%.

^{1}H NMR (500 MHz, $CDCl_3$, 300 K): δ = 7.39-7.31 (m, 5 H, *H*-7, *H*-7‘, *H*-8, *H*-8‘,*H*-9), 4.66 (s, 2 H, *H*-5), 3.98 (tt, ^3J (H-F) = 13.7 Hz, ^4J (H-F) = 1.5 Hz, 2 H, *H*-1), 3.89 (tt, ^3J (H-F) = 13.2 Hz, ^4J (H-F) = 1.6 Hz, 2 H, *H*-4), 2.63 (s, 1 H, *OH*) ppm.

^{13}C NMR (125 MHz, $CDCl_3$, 300 K): δ = 136.1 (*C*-6), 128.7 (*C*-8), 128.5 (*C*-9), 128.0 (*C*-7),117.1 (dtd, ^1J (C-F) = 253.3 Hz, ^2J (C-F) = 30.0 Hz, *C*-2), 115.1 (dtd, ^1J (C-F) = 253.3 Hz, ^2J (C-F) = 31.1 Hz, *C*-3), 74.6 (*C*-5), 66.7 (t, ^2J (C-F) = 27.6 Hz, *C*-4), 60.6 (^2J (C-F) = 27.3 Hz, *C*-1) ppm.

^{19}F NMR (500 MHz, $CDCl_3$, 300 K): δ = -122.4 (broad s, 2 F, *F*-2), -125.0 (broad s, 2 *F*, F-3) ppm.

FT-IR: $\tilde{\nu}$ = 3414 (br, w), 2880 (br, w), 2249 (w), 1741 (w), 1498 (w), 1455 (w), 1370 (w), 1216 (m), 1184 (m), 1111 (s), 1027 (m), 932 (m), 913 (m), 781 (m), 751 (s), 740 (s), 698 (s), 672 (m), 649 (m), 595 (m), 555 (m), 569 (m), 526 (s) cm^{-1}.

MS (EI, 70 eV): *m/z* (%) = 252.08 (29) $[M]^{+\cdot}$, 107.05 (40) $[C_7H_7O]^{+\cdot}$, 91.06 (100) $[C_7H_7]^{+\cdot}$.

MS (EI, HR, 70 eV): $C_{11}H_{12}F_4O_2$ *m/z* = calc.: 252.07734, found: 252.07719, diff.: -0.60 ppm.

Figure S1: ^{1}H NMR spectrum of compound **4**, measured in $CDCl_3$ at 300 K.

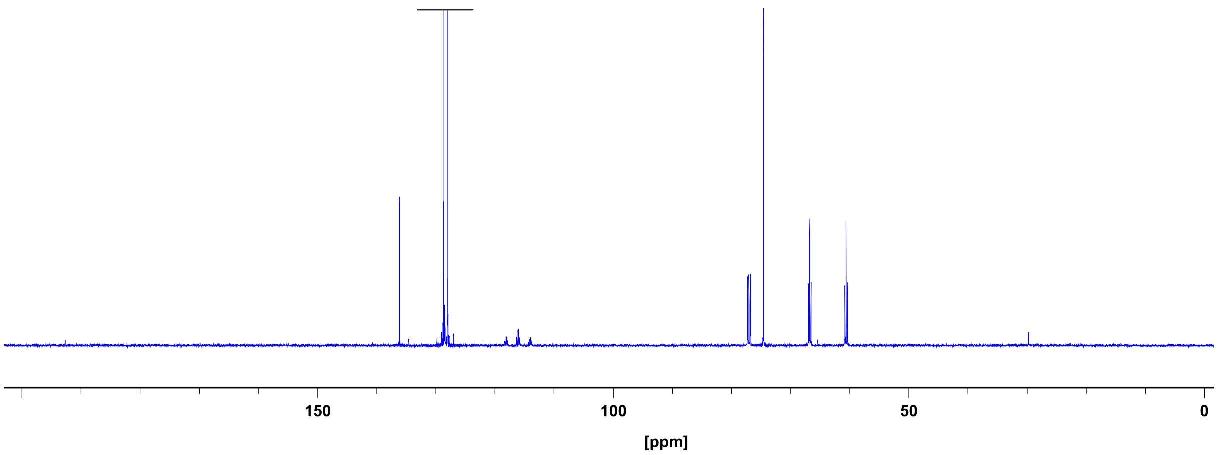

Figure S2: ^{13}C NMR spectrum of compound **4**, measured in $CDCl_3$ at 300 K.

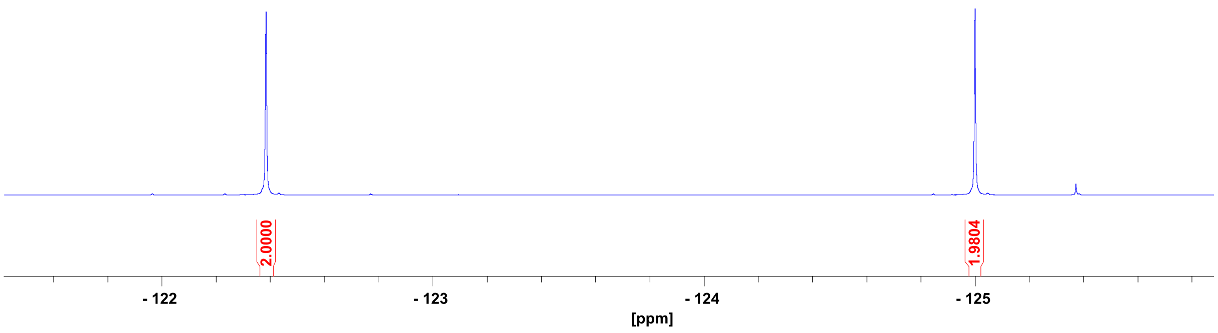

Figure S3: ^{19}F NMR spectrum of compound **4**, measured in $CDCl_3$ at 300 K.

6-Benzyloxy-2,2,3,3,4,4,5,5-octafluorohexan-1-ol (5)

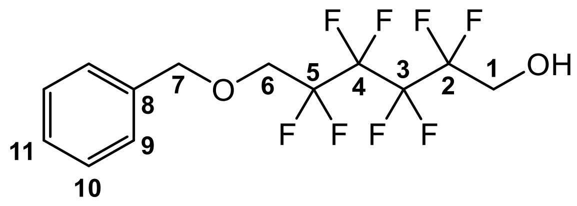

Yield: 50%.

^{1}H NMR (500 MHz, $CDCl_3$, 300 K): δ = 7.40-7.31 (m, 5 H, *H*-9, *H*-9‘, *H*-10, *H*-10‘, *H*-11), 4.68 (s, 2 H, *H*-7), 4.08 (tdt, ^3J (H-F) = 14.2 Hz, ^3J (OH-H) = 7.5 Hz, ^4J (H-F) = 1.5 Hz, 2 H, *H*-1), 3.94 (tt, ^3J (H-F) = 14.1 Hz, ^4J (H-F) = 1.6 Hz, 2 H, *H*-6), 1.80 (t, ^3J (OH-H) = 7.5 Hz, 1 H, *OH*) ppm.

^{13}C NMR (125 MHz, $CDCl_3$, 300 K): δ = 136.5 (*C*-8), 128.6 (*C*-10), 128.3 (*C*-11),

127.8 (*C*-9), 117.8-113.4 (*C*-2-5), 74.4 (*C*-7), 66.7 (t, ^2J (C-F) = 25.4 Hz, *C*-6), 60.7 (t, ^2J (C-F) = 26.1 Hz, *C*1) ppm.

Due to the low electron density and multiple couplings, the carbon atoms C-2, C-3, C-4 and C-5 are not visible in the ^{13}C NMR. The range was determined by 2D spectra (see Figure S7).

^{19}F NMR (500 MHz, $CDCl_3$, 300 K): δ = -120.0 (td, ^3J (H-F) = 13.3 Hz, ^4J (H-F) = 3.0 Hz, 2F, *F*-5), -123.1 to -123.2 (m, 2 F, *F*-2), -124.2 to -124.3 (m, 2 F, *F*-4), -124.5 to -124.6 (m, 2 F, *F*-3) ppm.

FT-IR: $\tilde{\nu}$ = 2883 (br, w), 2190 (w), 1705 (w), 1498 (w), 1456 (m), 1370 (w), 1168 (s), 1121 (s), 1030 (m), 992 (m), 937 (m), 866 (m), 821 (m), 738 (s), 698 (s) cm^{-1}.

MS (EI, 70 eV): *m/z* (%) = 352.08 (21) $[M]^{+\cdot}$, 107.05 (9) $[C_7H_7O]^{+\cdot}$, 91.06 (100) $[C_7H_7]^{+\cdot}$.

MS (EI, HR, 70 eV): $C_{13}H_{12}F_8O_2$ *m/z* = calc.: 352.07095, found: 352.07089, diff.: -0.19 ppm.

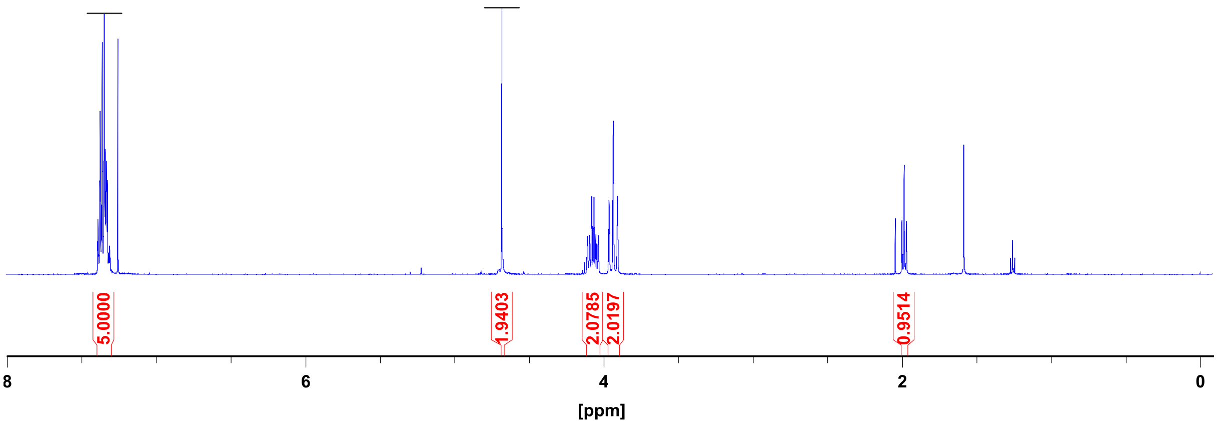

Figure S4: ^{1}H NMR spectrum of compound **5**, measured in $CDCl_3$ at 300 K.

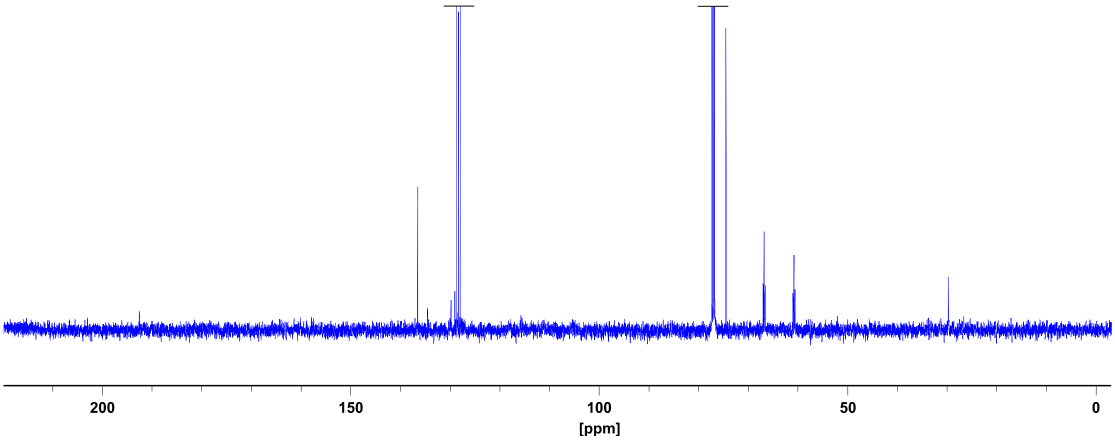

Figure S5: ^{13}C NMR spectrum of compound **5**, measured in $CDCl_3$ at 300 K.

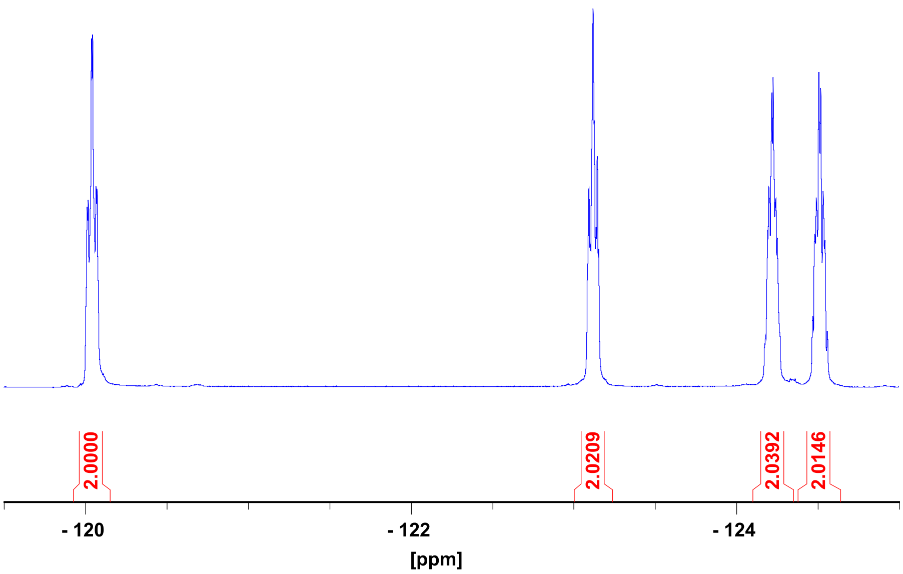

Figure S6: ^{19}F NMR spectrum of compound **5**, measured in $CDCl_3$ at 300 K.

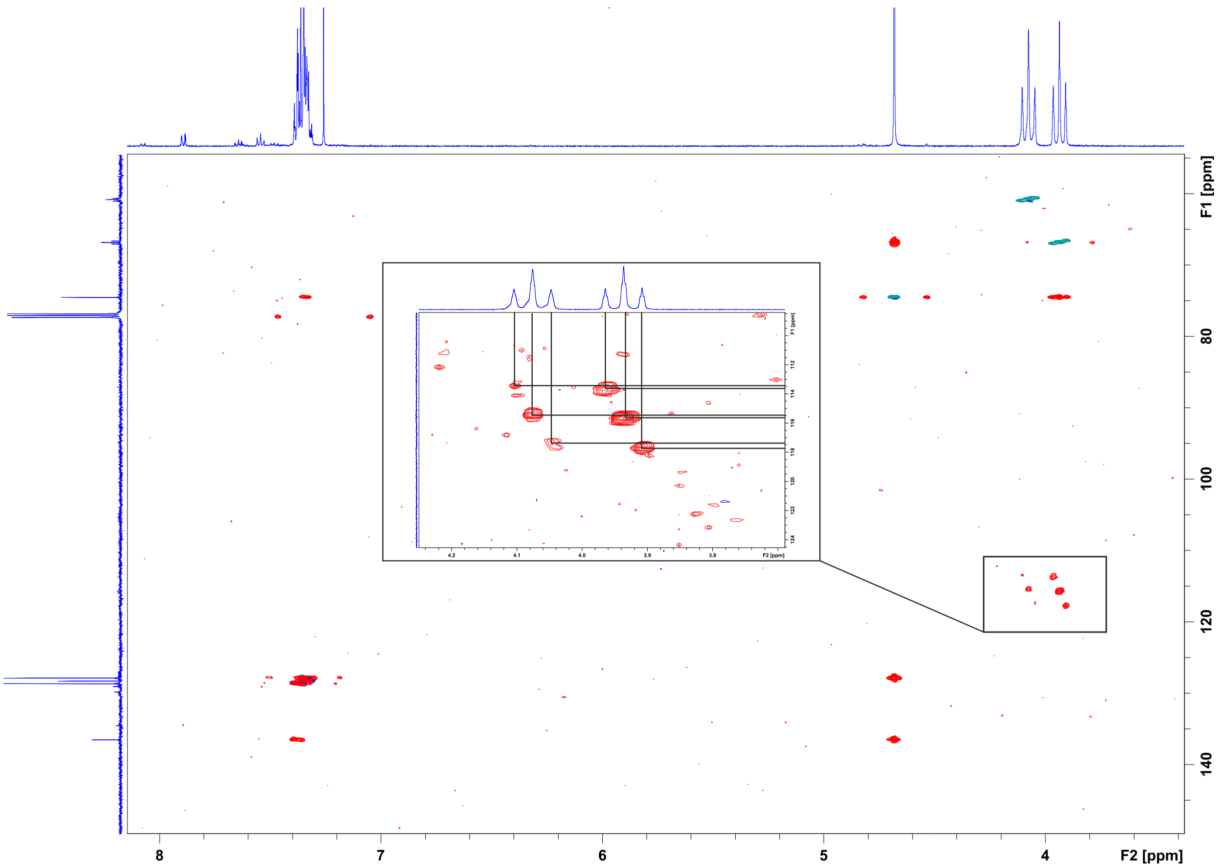

Figure S7: 2D spectra of compound **5**, showing the HSQC in blue/turquoise and the HMBC in red.

8-Benzyloxy-2,2,3,3,4,4,5,5,6,6,7,7-dodecafluorooctan-1-ol (6)

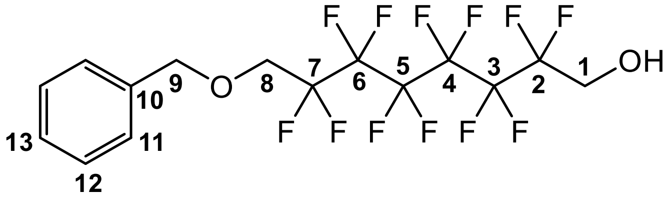

Yield: 40%.

^{1}H NMR (500 MHz, $CDCl_3$, 300 K): δ = 7.40-7.31 (m, 5 H, *H*-11, *H*-11‘, *H*-12, *H*-12‘, *H*-13), 4.68 (s, 2 H, *H*-9), 4.08 (tdt, ^{3}J (H-F) = 14.1 Hz, ^{3}J (OH-H) = 7.6 Hz ^{4}J (H-F) = 1.4 Hz, 2 H, *H*-1), 3.94 (tt, ^{3}J (H-F) = 13.9 Hz, ^{4}J (H-F) = 1.5 Hz, 2 H, *H*-8), 1.87 (t, ^{3}J (OH-H) = 7.6 Hz, 1 H, *OH*) ppm.

^{13}C NMR (125 MHz, $CDCl_3$, 300 K): δ = 136.3 (*C*-10), 128.6 (*C*-12), 128.3 (*C*-13), 127.8 (*C*-11), 117.8-113.2 (*C*-2-7), 74.4 (*C*-9), 66.7 (t, ^{2}J (C-F) = 25.6 Hz, *C*-8), 60.7 (t, ^{2}J (C-F) = 26.2 Hz, *C*-1) ppm.

Due to the low electron density and multiple couplings, the carbon atoms C-2, C-3, C-4, C-5, C-6 and C-7 are not visible in the ^{13}C NMR. The range was determined by 2D spectra (see Figure S11).

^{19}F NMR (500 MHz, $CDCl_3$, 300 K): δ = -119.8 to -119.9 (m, 2 F, *F*-7), -122.5 to 122.7 (m, 4 F, *F*-4, *F*-5), -122.9 to 123.0 (m, 2 F, *F*-2), -123.8 to -123.9 (m, 2 F, *F*-6), -124.1 to -124.2 (m, 2 F, *F*-3) ppm.

FT-IR: $\tilde{\nu}$ = 1707 (m), 1498 (w), 1456 (m), 1370 (m), 1191 (s), 1139 (s) 1029 (w), 939 (w), 908 (w), 837 (w), 791 (w), 745 (m), 698 (m), 657 (s) cm^{-1}.

MS (EI, 70 eV): *m/z* (%) = 452.06 (21) $[M]^{+\cdot}$, 107.05 (3) $[C_7H_7O]^{+\cdot}$, 91.06 (100) $[C_7H_7]^{+\cdot}$.

MS (EI, HR, 70 eV): $C_{15}H_{12}F_{12}O_2$ *m/z* = calc.: 452.06457, found: 452.06435, diff.: -0.48 ppm.

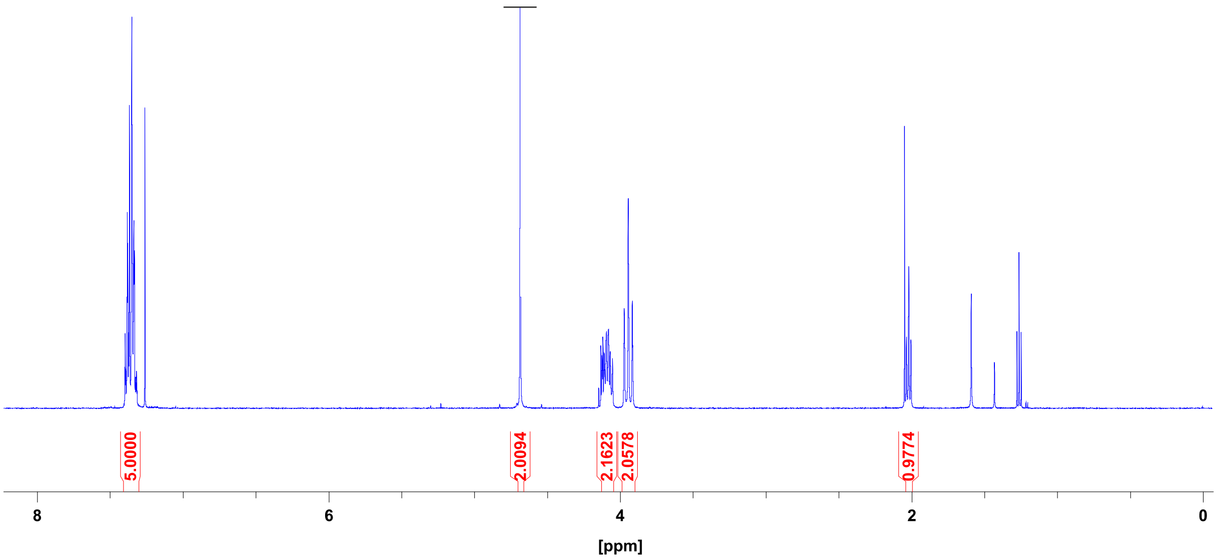

Figure S8: ^{1}H NMR spectrum of compound **6**, measured in $CDCl_3$ at 300 K.

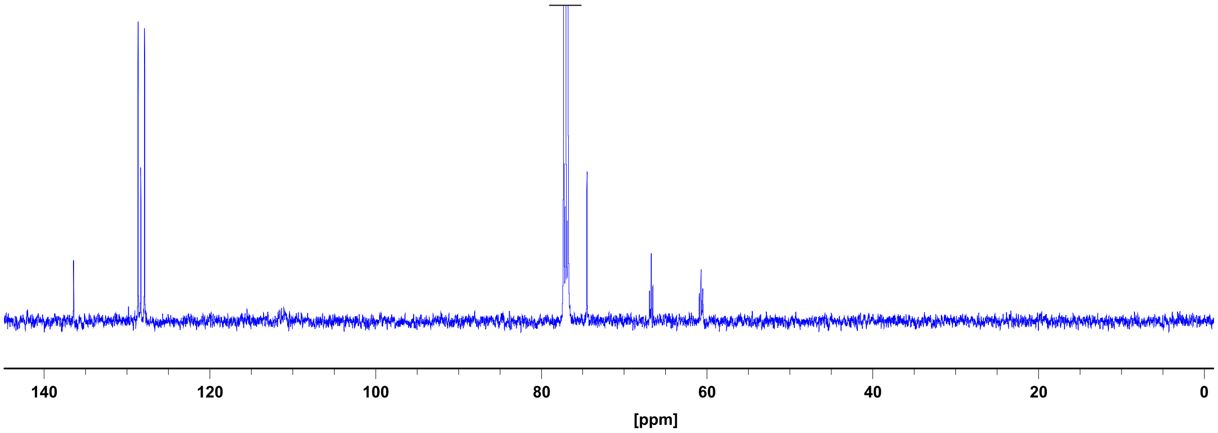

Figure S9: ^{13}C NMR spectrum of compound **6**, measured in $CDCl_3$ at 300 K.

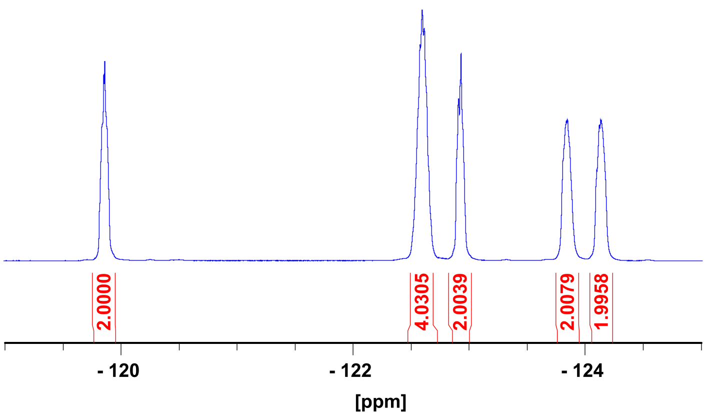

Figure S10: ^{19}F NMR spectrum of compound **6**, measured in $CDCl_3$ at 300 K.

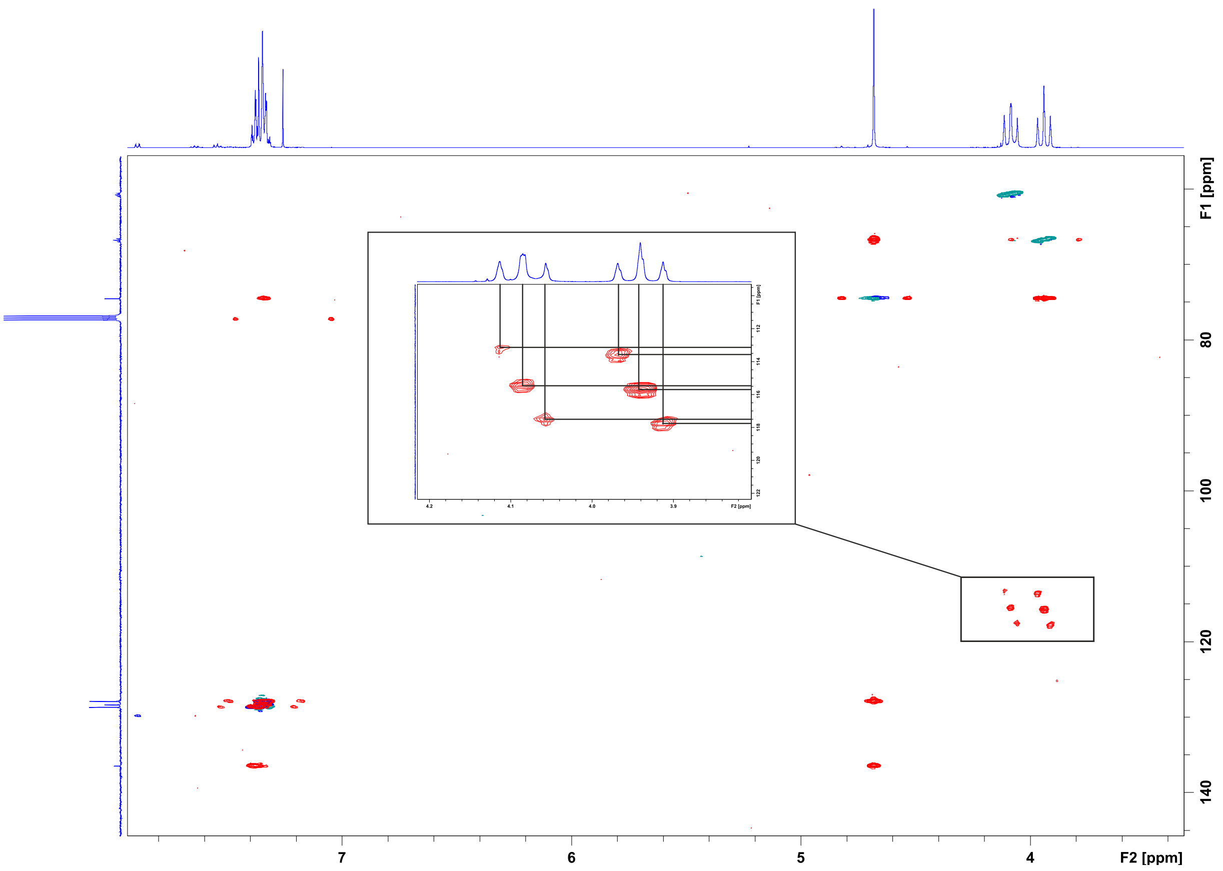

Figure S11: 2D spectra of compound **6**, showing the HSQC in blue/turquoise and the HMBC in red.

3.2 General procedure for oxidation of 4–6

One equivalent of the corresponding alcohol **4–6** was dissolved in acetonitrile (100 mL) and 2,2,6,6-tetramethylpiperidinyloxyl (TEMPO, 0.029 equiv) and potassium bromide (0.136 equiv) were added with stirring. Separately, a solution of sodium hypochlorite (4.2 equiv) and sodium bicarbonate (4.2 equiv) was prepared. This solution was added in three portions. The first addition was made at −10 °C, the second after 16 h at room temperature, and the third after a further 6 h, also at room temperature. The reaction mixture was then stirred for a further 54 h at room temperature before the reaction was stopped by adding conc. sulfuric acid (5 mL), and dist. water (100 mL). Subsequently, the reaction was extracted with diethyl ether (3 × 100 mL), the organic phases were dried over magnesium sulfate, filtered and the solvent was removed in vacuo. A clear, yellowish liquid was obtained. The raw product was not further worked up and was used without purification in the next stage.

4-Benzyloxy-2,2,3,3-tetrafluorobutanoic acid (7)

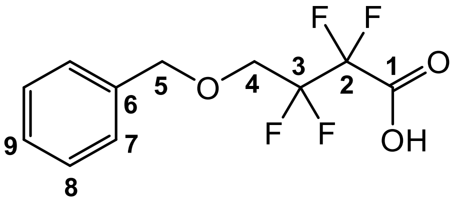

^{1}H NMR (500 MHz, $CDCl_3$, 300 K, TMS): δ = 7.37-7.28 (m, 5 H, *H*-7, *H*-7‘, *H*-8, *H*-8‘, *H*-9), 4.63 (s, 2 H, *H*-5), 3.93 (tt, ^3J (H-F) = 13.1 Hz, ^4J (H-F) = 1.5 Hz, 2 H, *H*-4) ppm.

^{13}C NMR (125 MHz, $CDCl_3$, 300 K, TMS): δ = 162.7 (t, ^2J (C-F) = 30.3 Hz, *C*-1), 136.2 (*C*-6), 128.6 (*C*-8), 128.4 (*C*-9), 128.0 (*C*-7), 115.3 (tt, ^1J (C-F) = 256.5 Hz, ^2J (C-F)

= 28.5 Hz, *C*-2), 108.7 (tt, ^{1}J (C-F) = 261.9 Hz, ^{2}J (C-F) = 31.2 Hz, *C*-3), 74.6 (*C*-5), 66.7 (t, ^{2}J (C-F) = 26.8 Hz, *C*-4) ppm.

^{19}F NMR (500 MHz, $CDCl_3$, 300 K): δ = -121.2 (s, 2 F, *F*-2), -122.4 (s, 2 F, *F*-3) ppm.

FT-IR: $\tilde{\nu}$ = 3490 (br, w), 2879 (br, w), 2526 (br, w), 1763 (m), 1497 (w), 1455 (m), 1370 (m), 1246 (m), 1209 (m), 1127 (s), 1031 (m), 987 (m), 777 (m), 750 (m), 714 (m), 698 (s), 594 (m), 537 (m) cm^{-1}.

MS (EI, 70 eV): *m/z* (%) = 266.06 (26) $[M]^{+\cdot}$, 159.01 (9) $[M-C_7H_7O]^{+}$, 107.05 (42) $[C_7H_7O]^{\cdot}$, 91.06 (100) $[C_7H_7]^{+\cdot}$.

MS (EI, HR, 70 eV): $C_{11}H_{10}F_4O_3$ *m/z* = calc.: 266.05661, found: 266.05651, diff.: -0.35 ppm.

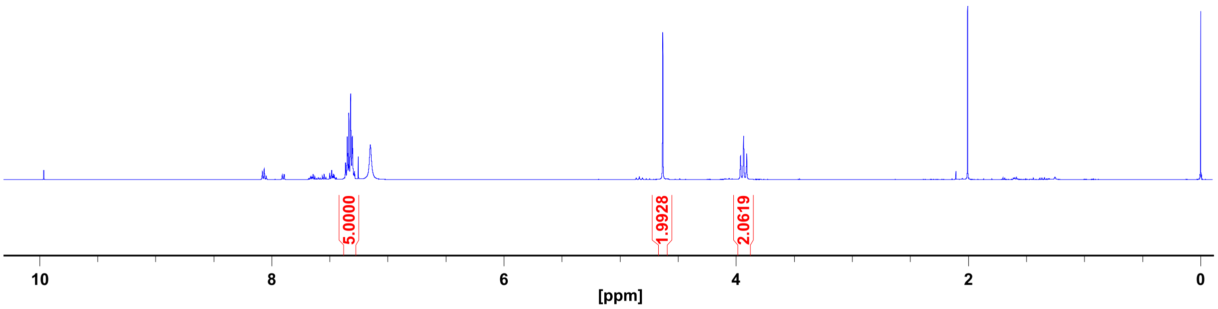

Figure S12: ^{1}H NMR spectrum of compound **7**, measured in $CDCl_3$ at 300 K. The reaction was not worked up and an NMR of the crude product was measured. It is assumed that the additional signals are the by-product **35** (see below).

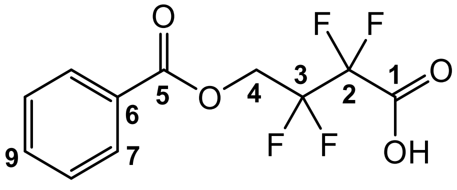

35

Figure S13: It is assumed that radical oxidation leads to the formation of the by-product **35**.

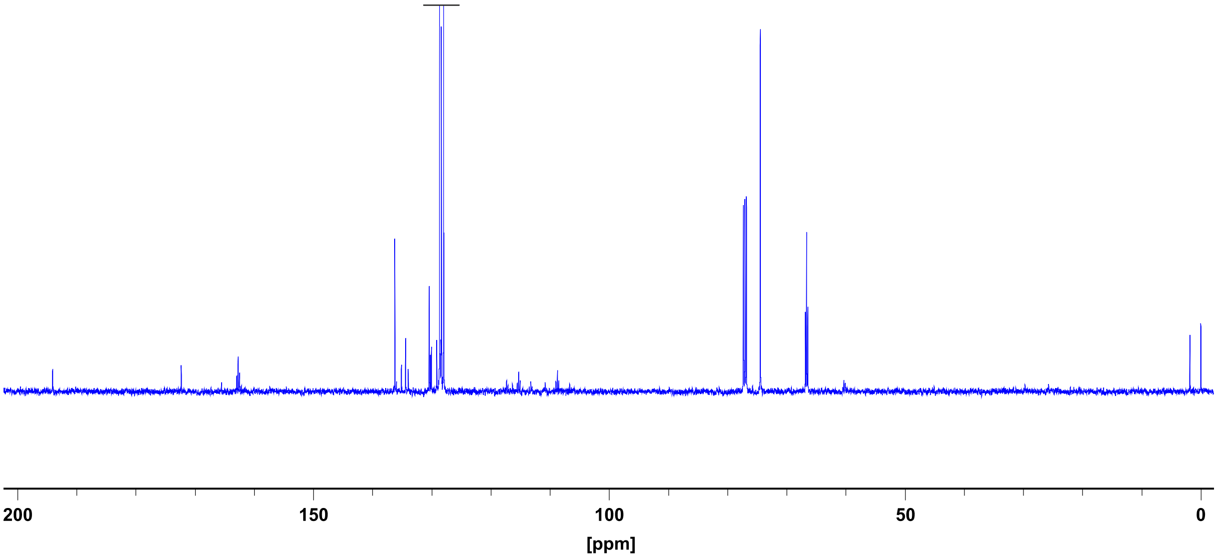

Figure S14: ^{13}C NMR spectrum of compound **7**, measured in $CDCl_3$ at 300 K. It is assumed that the additional signals are the by-product **35** (see above).

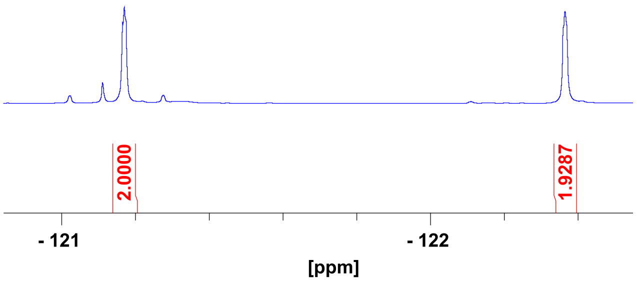

Figure S15: ^{19}F NMR spectrum of compound **7**, measured in $CDCl_3$ at 300 K. It is assumed that the additional signals are the by-product **35** (see above).

6-Benzyloxy-2,2,3,3,4,4,5,5-octafluorohexanoic acid (8)

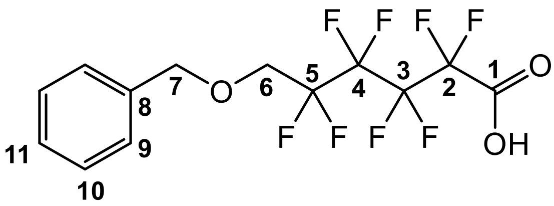

^{1}H NMR (500 MHz, $CDCl_3$, 300 K): δ = 7.40-7.31 (m, 5 H, *H*-9, *H*-9‘, *H*-10, *H*-10‘, *H*-11), 4.68 (s, 2 H, *H*-7), 3.94 (tt, ^{3}J (H-F) = 13.9 Hz, ^{4}J (H-F) = 1.5 Hz, 2 H, *H*-6) ppm.

^{13}C NMR (125 MHz, $CDCl_3$, 300 K): δ = 160.7 (t, ^{2}J (C-F) = 30.1 Hz, *C*-1), 136.3 (*C*-8), 128.3 (*C*-10), 128.3 (*C*-11), 127.9 (*C*-9), 117.7-113.6 (*C*-3-5), 74.5 (*C*-7), 66.6 (t, ^{2}J (C-F) = 25.2 Hz, *C*-6) ppm.

Due to the large number of ^{19}F-coupled C atoms, it was not possible to obtain sufficient signal intensity for the *C*-2 atom. For C-3–5 the range was determined by 2D spectra (see Figure S20).

^{19}F NMR (500 MHz, $CDCl_3$, 300 K): δ = -119.7 (t, ^{3}J (H-F)= 10.7 Hz, 2 F, *F*-2), -119.9 to -120.0 (m, 2 F, *F*-5), -123.5 to -123.7 (m, 4 F, *F*-3, F-4) ppm.

FT-IR: $\tilde{\nu}$ = 2946 (br, w), 1774 (m), 1456 (w), 1272 (m), 1179 (s), 1122 (s), 1029 (m), 951 (w), 867 (m), 846 (m), 750 (m), 698 (s) cm^{-1}.

MS (EI, 70 eV): *m/z* (%) = 366 (21) $[M]^{+\cdot}$, 107.05 (8) $[C_7H_7O]^{+\cdot}$, 91.06 (100) $[C_7H_7]^{+\cdot}$.

MS (EI, HR, 70 eV): $C_{13}H_{10}F_8O_3$ *m/z* = calc.: 366.05022, found: 366.05018, diff.: -0.11 ppm.

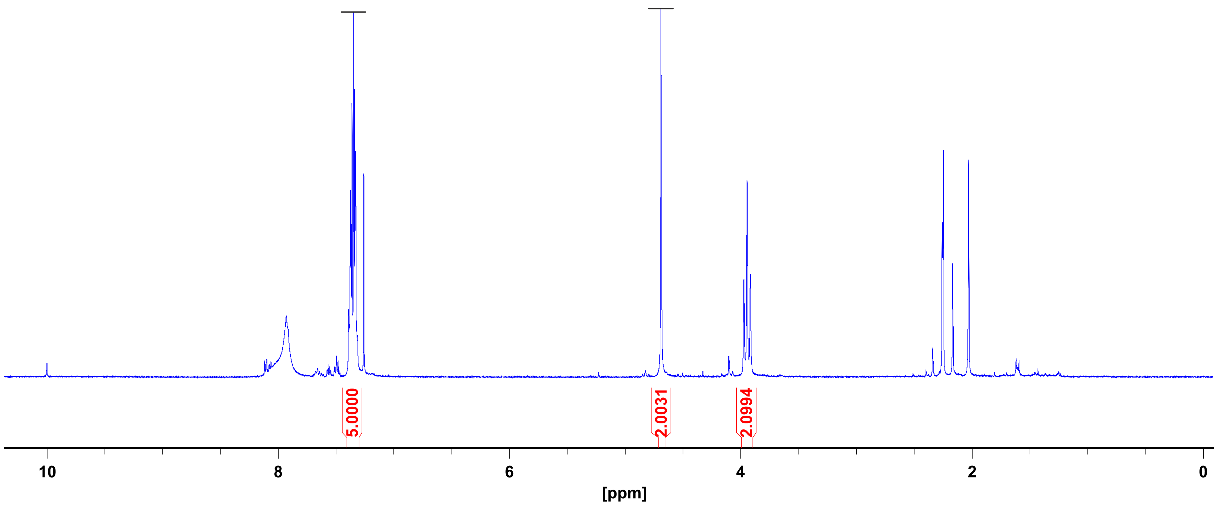

Figure S16: ^{1}H NMR spectrum of compound **8**, measured in $CDCl_3$ at 300 K. The reaction was not worked up and an NMR of the crude product was measured. It is assumed that the additional signals are the by-product **36** (see below).

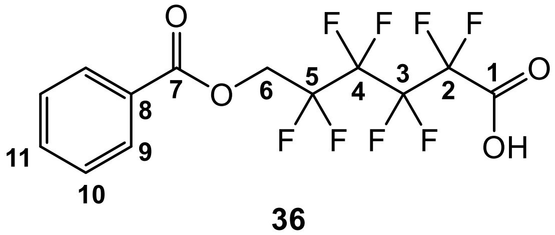

36

Figure S17: It is assumed that radical oxidation leads to the formation of the by-product **36**.

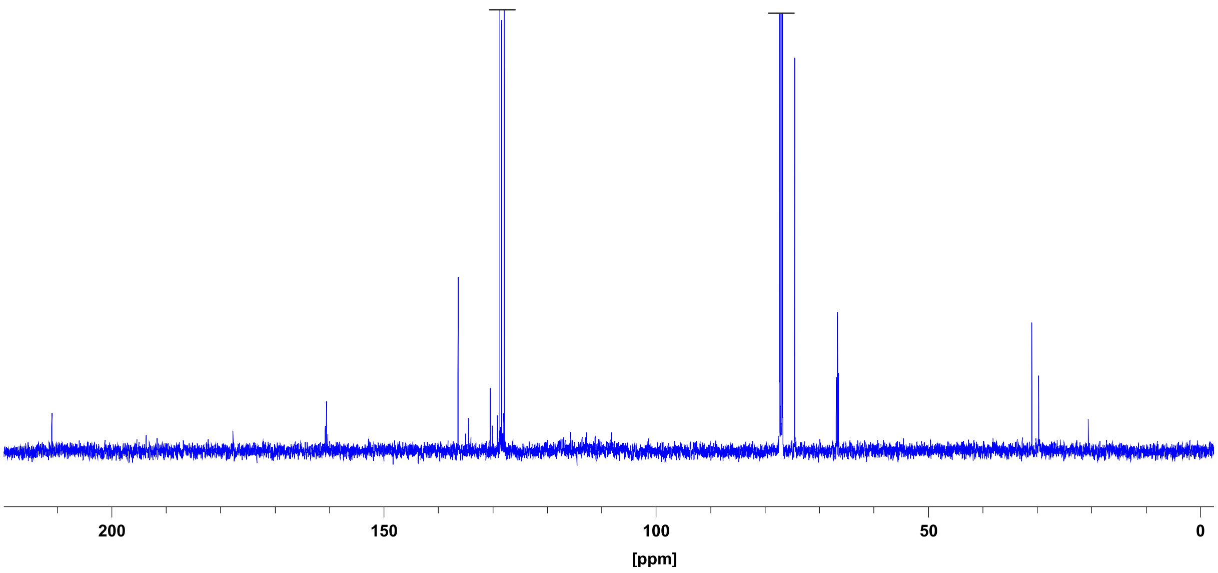

Figure S18: ^{13}C NMR spectrum of compound **8**, measured in $CDCl_3$ at 300 K. It is assumed that the additional signals are the by-product **36** (see above).

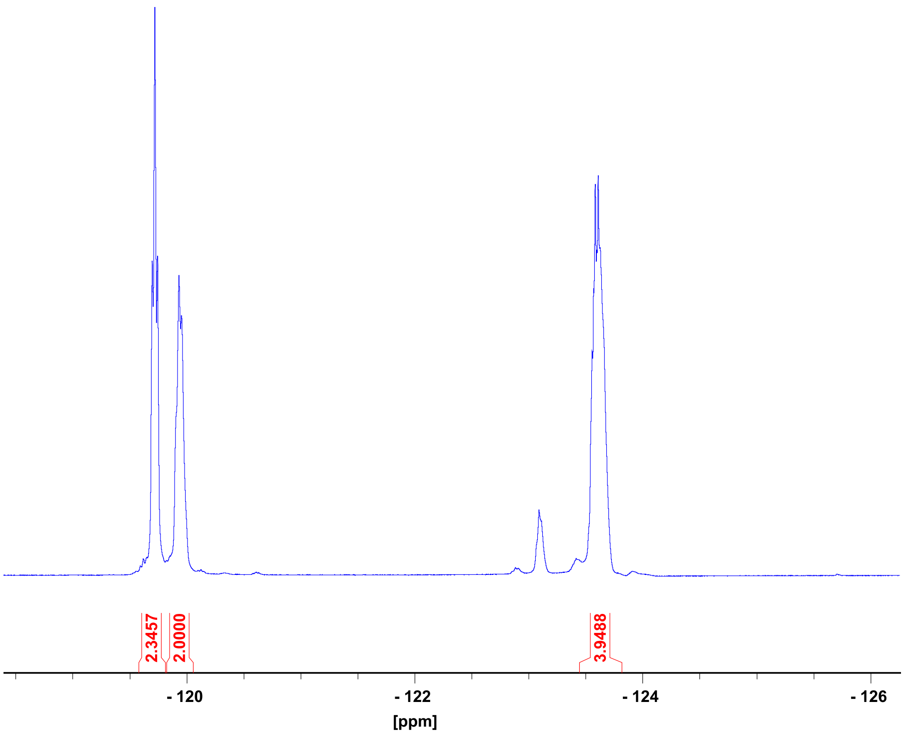

Figure S19: ^{19}F NMR spectrum of compound **8**, measured in $CDCl_3$ at 300 K. It is assumed that the additional signals are the by-product **36** (see above).

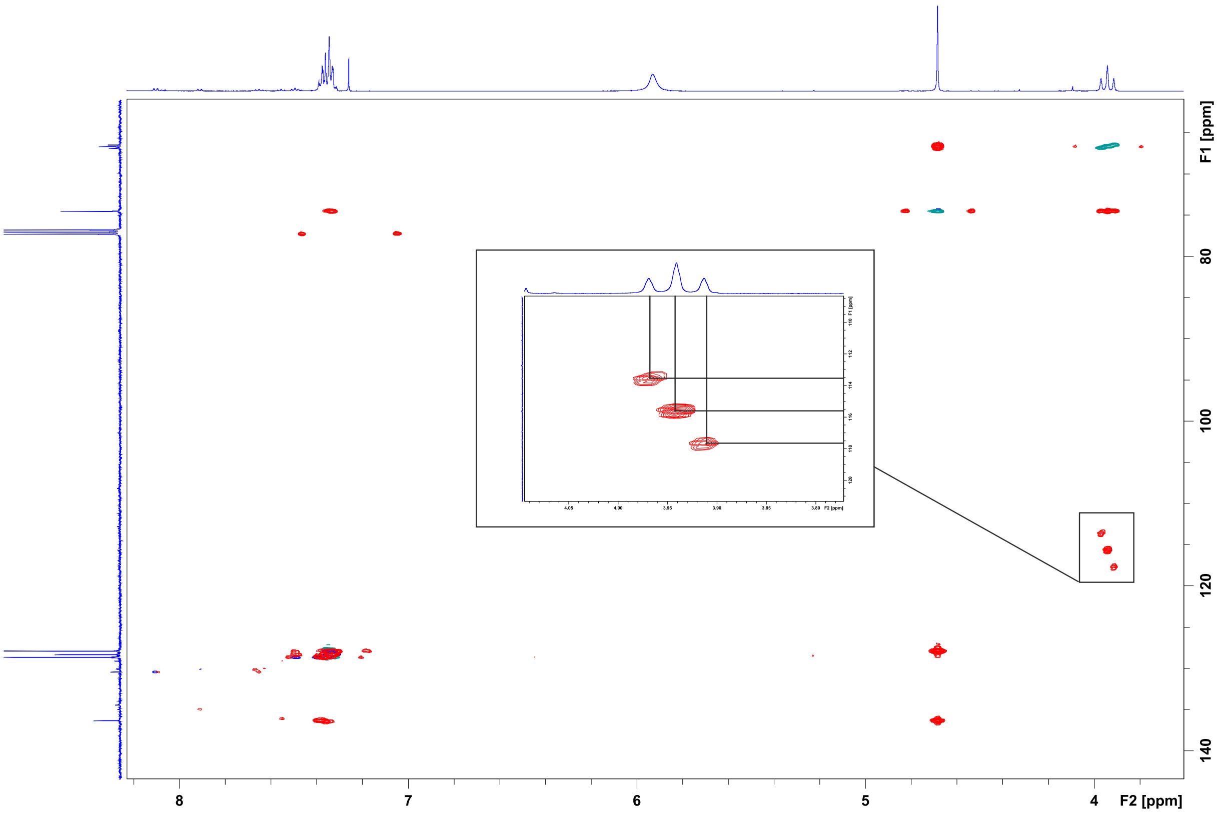

Figure S20: 2D spectra of compound **8**, showing the HSQC in blue/turquoise and the HMBC in red.

8-Benzyloxy-2,2,3,3,4,4,4,5,5,6,6,7,7-dodecafluoroctanoic acid (9)

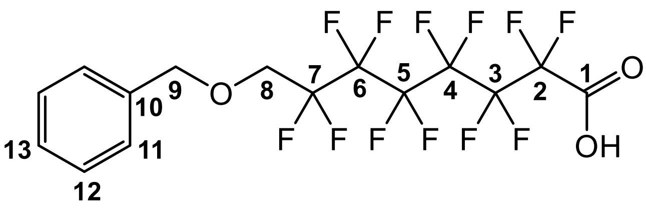

^{1}H NMR (500 MHz, $CDCl_3$, 300 K, TMS): δ = 7.40-7.31 (m, 5 H, *H*-11, *H*-11‘, *H*-12, *H*-12‘, *H*-13), 5.90 (s, 1 H, COO*H*), 4.70 (s, 2 H, *H*-9), 3.96 (tt, ^3J (H-F) = 13.9 Hz, ^4J (H-F) = 1.5 Hz, 2 H, *H*-8) ppm.

^{13}**C NMR** (125 MHz, $CDCl_3$, 300 K, TMS): δ = 160.1 (t, ^2J (C-F) = 29.5 Hz, *C*-1), 136.3 (*C*-10), 128.4 (*C*-12), 128.3 (*C*-13), 127.9 (*C*-11), 117.8-113.6 (*C*-4-7), 74.5 (*C*-9), 66.6 (t, 2J (C-F) = 24.9 Hz, *C*-8) ppm.

Due to the large number of ^{19}F-coupled C atoms, it was not possible to obtain sufficient signal intensity for the *C*-2 and C-3 atom. For C-4-7 the range was determined by 2D spectra (see Figure 25).

^{19}**F NMR** (500 MHz, $CDCl_3$, 300 K): δ = -119.5 to -119.6 (m, 2 F, *F*-2), -119.8 to -119.9 (m, 2 F, *F*-7), -122.0 to -122.2 (m, 2 F, *F*-4), -122.5 to -122.7 (m, 2 F, *F*-5) -123.2 to -123.3 (m, 2 F, *F*-3), -123.8 to 123.9 (m, 2 F, *F*-6) ppm.

FT-IR: ṽ = 1774 (m), 1498 (w), 1456 (m), 1196 (s), 1137 (s), 1028 (m) 905 (w), 836 (w), 779 (w), 732 (m), 714 (w), 698 (m), 656 (m) cm^{-1}.

MS (ESI): *m/z* (%) = 465 (100) $[M-H]^{-\cdot}$, 374.99 (30) $[M-C_7H_7]^{-\cdot}$.

MS (ESI, HR): $C_{15}H_9F_{12}O_3$ *m/z* = calc.: 465.03656, found: 465.03706 diff.: -1.09 ppm.

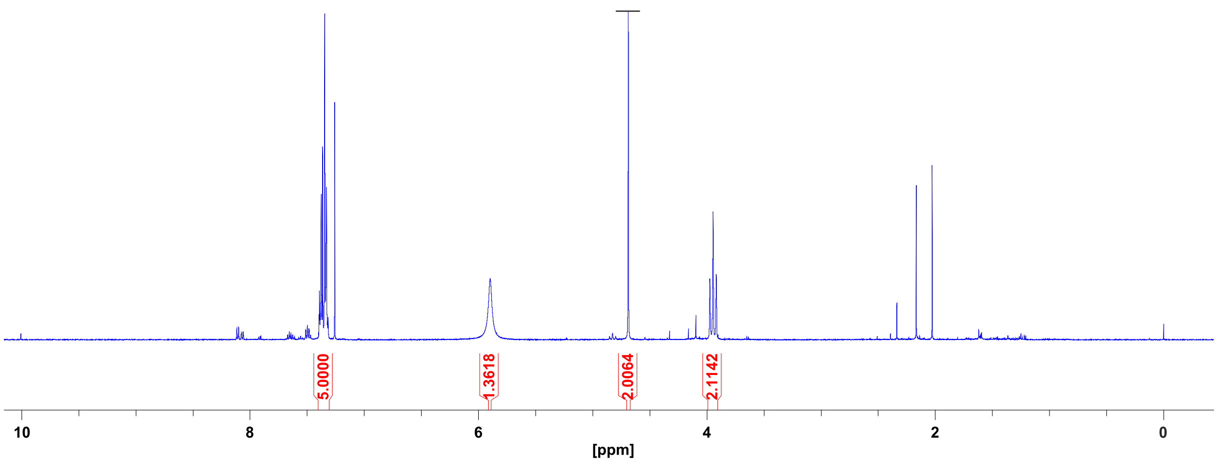

Figure S21: ^{1}H NMR spectrum of compound **9**, measured in $CDCl_3$ at 300 K. The reaction was not worked up and an NMR of the crude product was measured. It is assumed that the additional signals are the by-product **37** (see below).

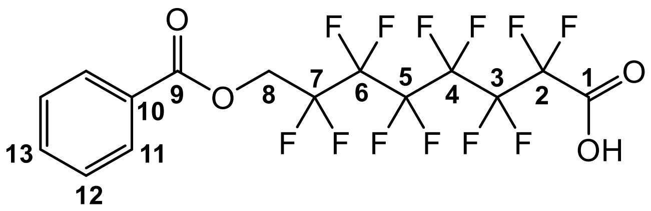

37

Figure S22: It is assumed that radical oxidation leads to the formation of the by-product **37**.

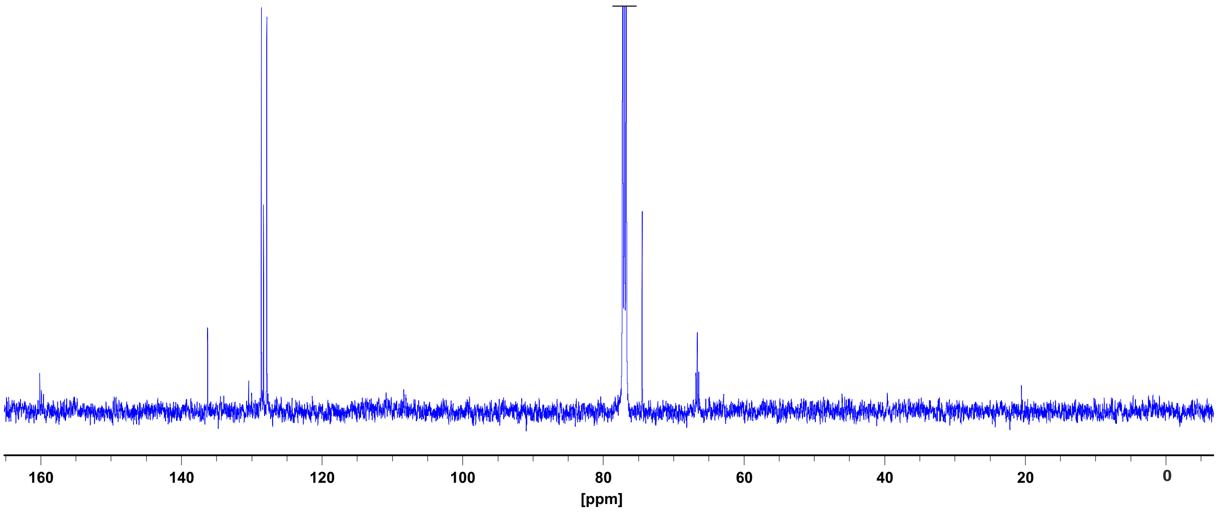

Figure S23: ^{13}C NMR spectrum of compound **9**, measured in $CDCl_3$ at 300 K. It is assumed that the additional signals are the by-product **37** (see above).

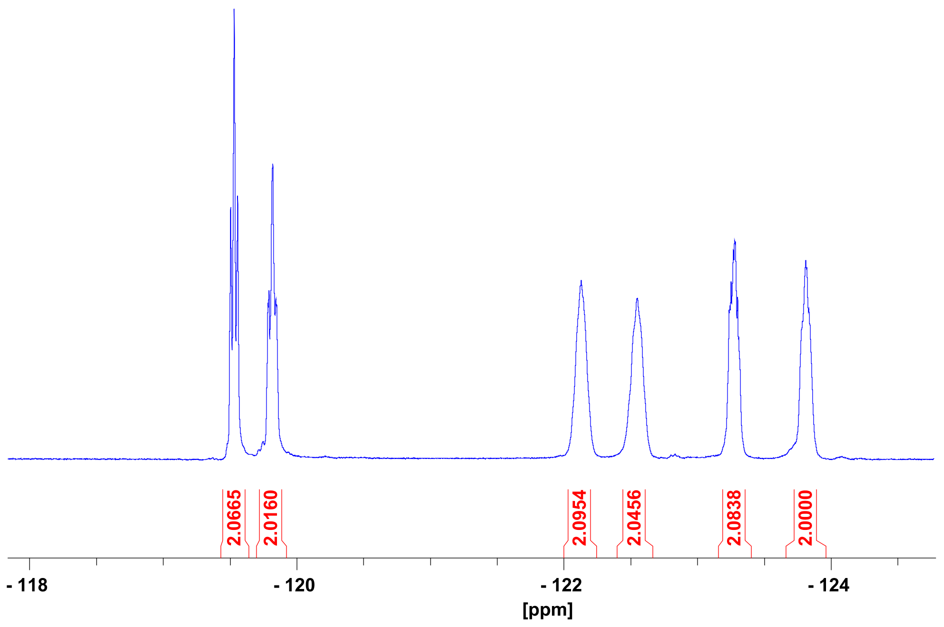

Figure S24: ^{19}F NMR spectrum of compound **9**, measured in $CDCl_3$ at 300 K.

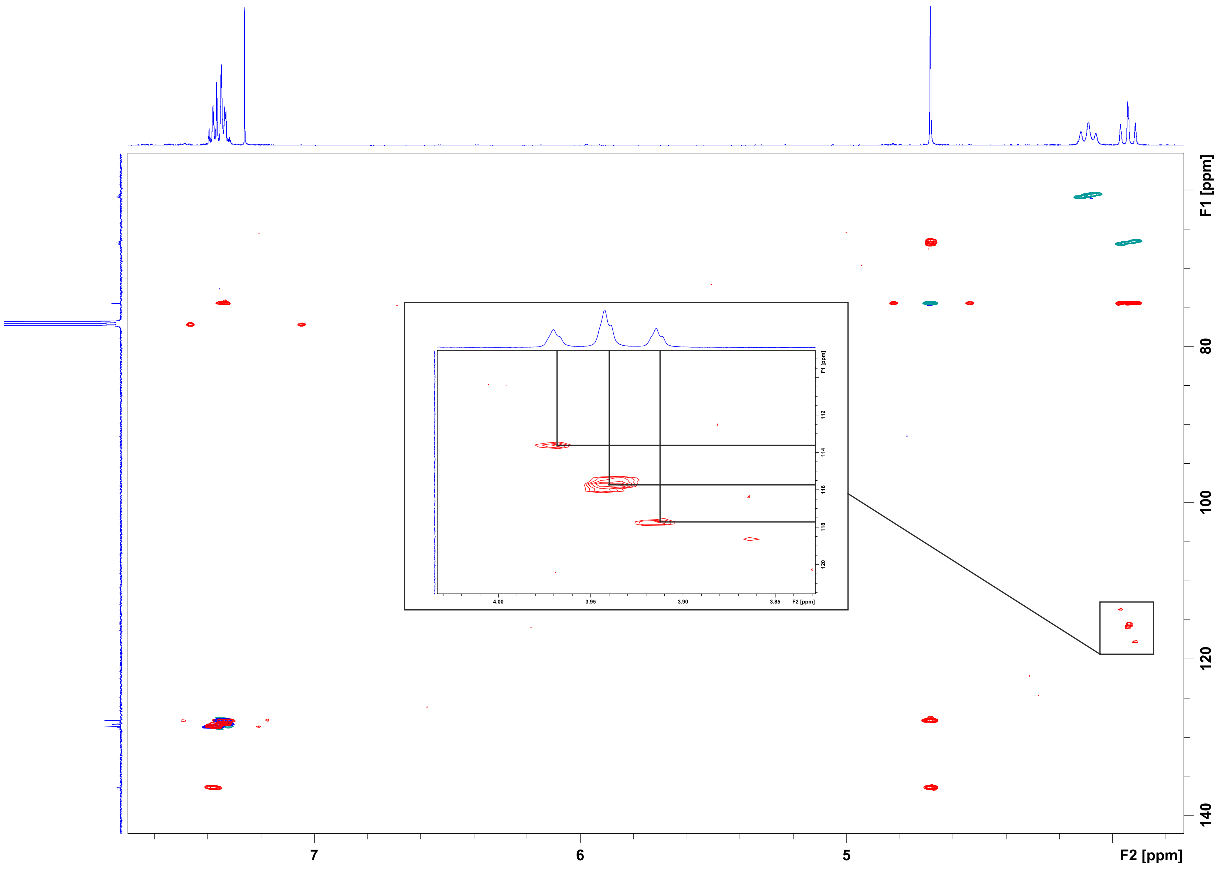

Figure S25: 2D spectra of compound **9**, showing the HSQC in blue/turquoise and the HMBC in red.

3.3 General procedure for the esterification of 7, 8 and 9

The corresponding crude product **7–9** (1 equiv) from the synthesis above and conc. sulfuric acid (300 µL) were dissolved in MeOH (100 mL) and stirred under reflux for 18 h. After cooling to room temperature, dist. water (100 mL) and saturated sodium chloride solution (100 mL) were added, extracted with diethyl ether (3 × 100 mL) and the organic phases were washed with dist. water (2 × 50 mL) and saturated sodium chloride solution (2 × 50 mL). The organic phase was dried over magnesium sulfate, filtered and the solvent removed in vacuo. The crude product was separated by flash column chromatography on silica gel (cyclohexane/ethyl acetate, gradient: 8%–60% ethyl acetate). A colorless liquid was obtained.

Methyl 4-benzyloxy-2,2,3,3-tetrafluoromethylbutanoate (10)

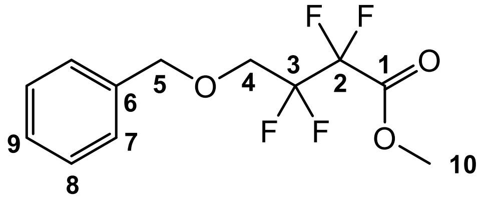

Yield (2 steps): 54 %.

^{1}H NMR (500 MHz, $CDCl_3$, 300 K): δ = 7.39-7.30 (m, 5 H, *H*-7, *H*-7‘, *H*-8, *H*-8‘, *H*-9), 4.60 (s, 2 H, *H*-5), 3.91 (tt, ^{3}J (H-F) = 13.7 Hz, ^{4}J (H-F) = 1.5 Hz, 2 H, *H*-4), 3.79 (s, 3 H, *H*-10) ppm.

^{13}C NMR (125 MHz, $CDCl_3$, 300 K): δ = 160.9 (*C*-1), 136.3 (*C*-6), 128.6 (*C*-8), 128.3 (*C*-9), 128.0 (*C*-7), 117.2-113.0 (*C*-2-3), 74.4 (*C*-5), 66.7 (t, ^{2}J (C-F) = 26.8 Hz, *C*-4), 53.9 (*C*-10) ppm.

Due to the large number of ^{19}F-coupled C atoms, it was not possible to obtain sufficient signal intensity for the *C*-1, C-2 and C-3 atom. For C-1 the shift was determined by 2D spectra. For C-2-3 the range was also determined by 2D spectra (see Figure S29).

^{19}F NMR (500 MHz, $CDCl_3$, 300 K): δ = -121.4 (m, 2 F, *F*-2), -122.2 (m, 2 F, *F*-3) ppm.

FT-IR: $\tilde{\nu}$ = 2960 (br, w), 1774 (s), 1498 (w), 1455 (m), 1442 (m), 1331 (m), 1309 (m), 1244 (m), 1205 (m), 1163 (s), 1116 (s), 1039 (s), 1000 (m), 929 (m), 816 (m), 742 (s), 698 (s), 594 (m), 571 (m) cm^{-1}.

MS (EI, 70 eV): *m/z* (%) = 280 (24) $[M]^{+\cdot}$, 173.06 (34) $[M-C_7H_7O]^+$, 107.07 (41) $[C_7H_7O]^{\cdot}$, 91.07 (100) $[C_7H_7]^{+\cdot}$.

MS (EI, HR, 70 eV): $C_{12}H_{12}F_4O_3$ *m/z* = calc.: 280.07226, found: 280.07226, diff.: -0.00 ppm.

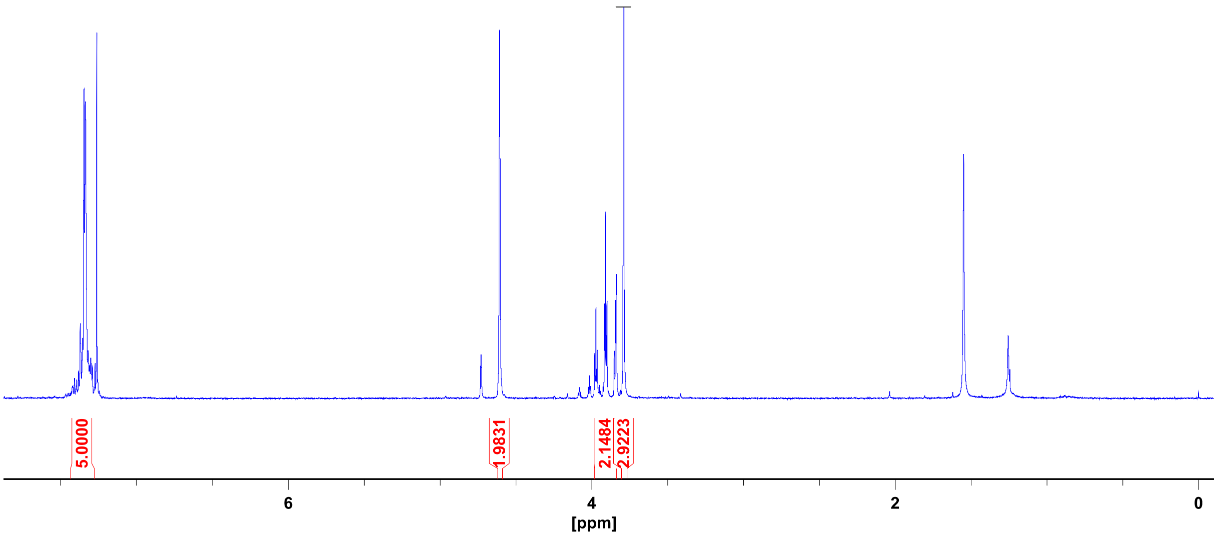

Figure S26: ^{1}H NMR spectrum of compound **10**, measured in $CDCl_3$ at 300 K.

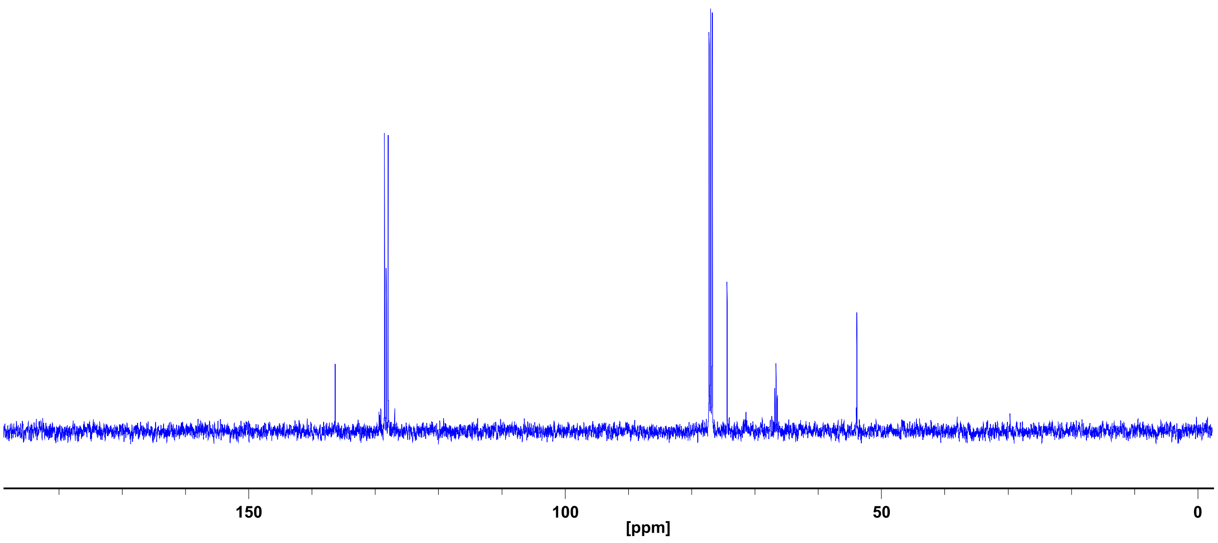

Figure S27: ^{13}C NMR spectrum of compound **10**, measured in $CDCl_3$ at 300 K.

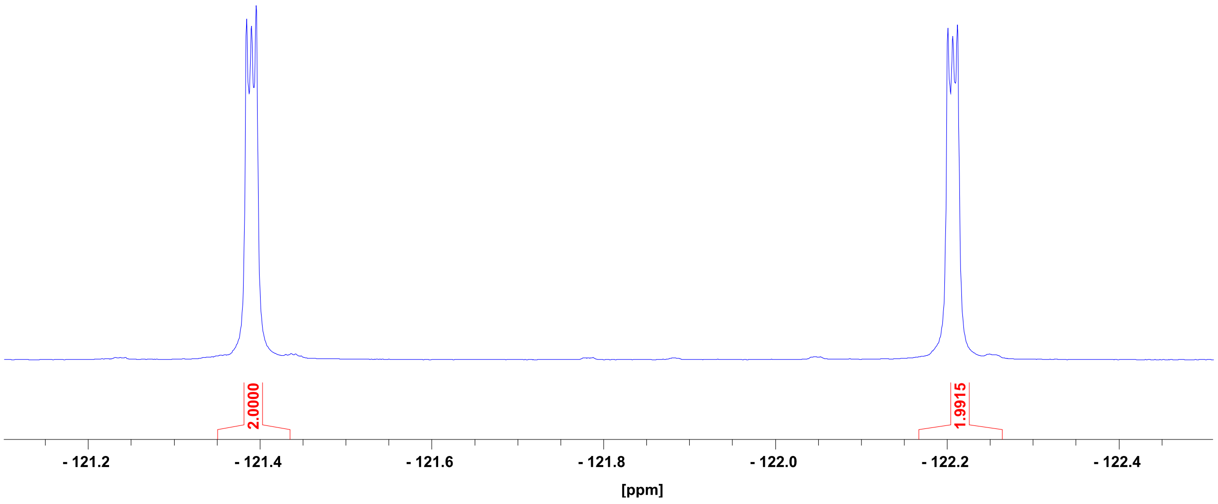

Figure S28: ^{19}F NMR spectrum of compound **10**, measured in $CDCl_3$ at 300 K.

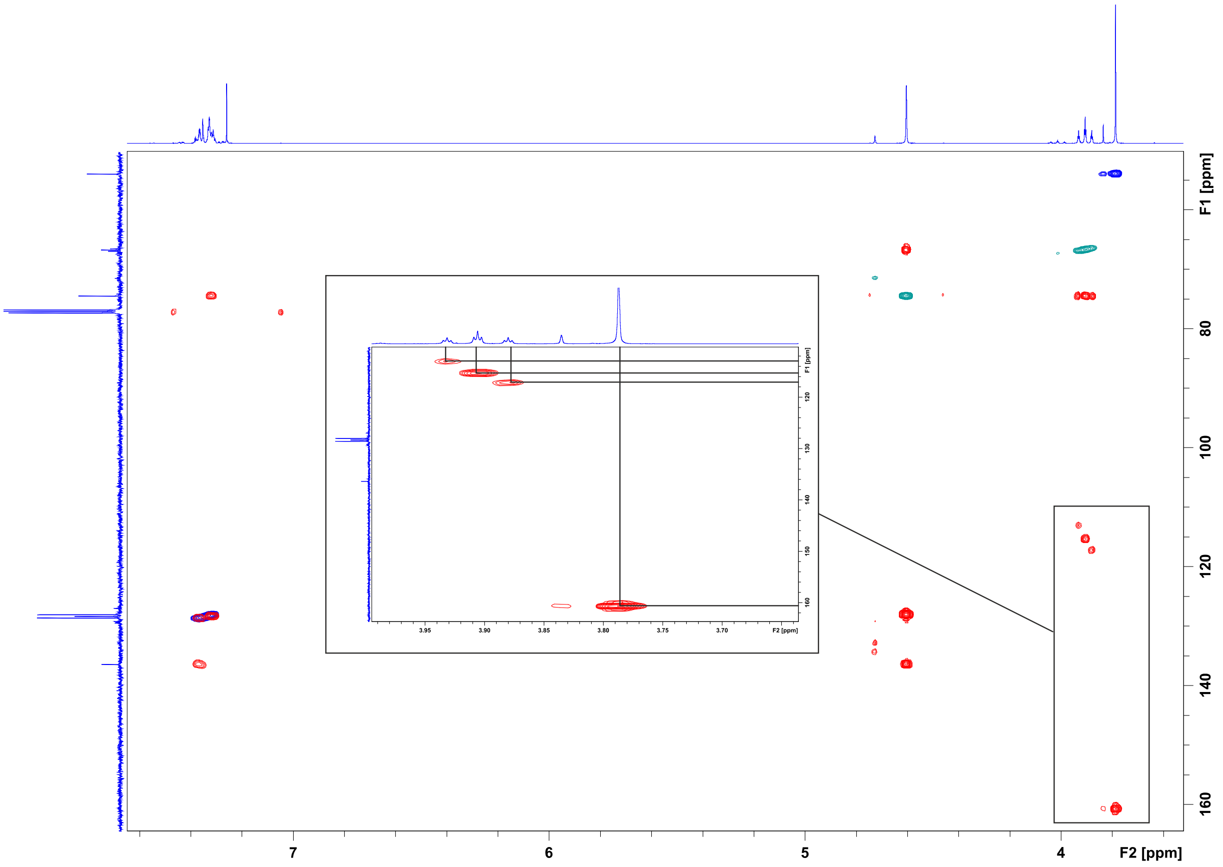

Figure S29: 2D spectra of compound **10**, showing the HSQC in blue/turquoise and the HMBC in red.

Methyl 6-benzyloxy-2,2,3,3,4,4,5,5-octafluoromethylhexanoate (11)

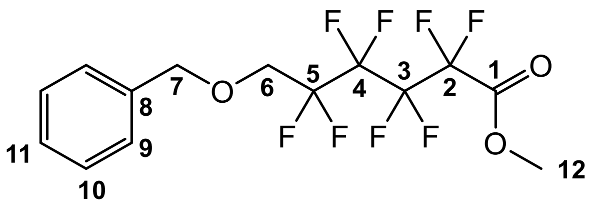

Yield (2 steps): 52 %.

^{1}H NMR (500 MHz, $CDCl_3$, 300 K): δ = 7.40-7.31 (m, 5 H, *H*-9, *H*-9‘, *H*-10, *H*-10‘, *H*-11), 4.67 (s, 2 H, *H*-7), 3.97 (s, 3 H, *H*-12), 3.92 (tt, ^3J (H-F) = 14.0 Hz, ^4J (H-F) = 1.6 Hz, 2 H, *H*-6) ppm.

^{13}C NMR (125 MHz, $CDCl_3$, 300 K): δ = 159.3 (t, ^2J (C-F) = 29.7, *C*-1), 136.4 (*C*-8), 128.6 (*C*-10), 128.3 (*C*-11), 127.8 (*C*-9), 117.7-111.2 (*C*-2-5), 74.4 (*C*-7), 66.7 (t, ^2J (C-F) = 25.1, *C*-6), 54.5 (*C*-12) ppm.

For C-2-5 the range was determined by 2D spectra (see Figure S34).

^{19}F NMR (500 MHz, $CDCl_3$, 300 K): δ = -119.1 to -119.2 (m, 2 F, *F*-2), -120.0 (td, ^3J (H-F) = 13.1 Hz, ^4J (H-F) = 3.20 Hz, 2 F, *F*-5), -123.6 to -123.7 (m, 2 F, *F*-3), -123.8 to -123.9 (m, 2 F, *F*-4) ppm.

FT-IR: $\tilde{\nu}$ = 2964 (br, w), 1782 (s), 1498 (w), 1456 (m), 1442 (m), 1370 (w) 1322 (m), 1266 (w), 1187 (s), 1137 (s), 1048 (m), 1029 (w), 959 (m), 868 (m), 807 (m), 746 (m), 699 (m) cm^{-1}.

MS (EI, 70 eV): *m/z* (%) = 380 (23) $[M]^{+\cdot}$, 273.02 (3) $[M-C_7H_7O]^+$, 107.05 (14) $[C_7H_7O]^{\cdot}$, 91.06 (100) $[C_7H_7]^{+\cdot}$.

MS (EI, HR, 70 eV): $C_{14}H_{12}F_8O_3$ *m/z* = calc.: 380.06587, found: 380.06576, diff.: -0.29 ppm.

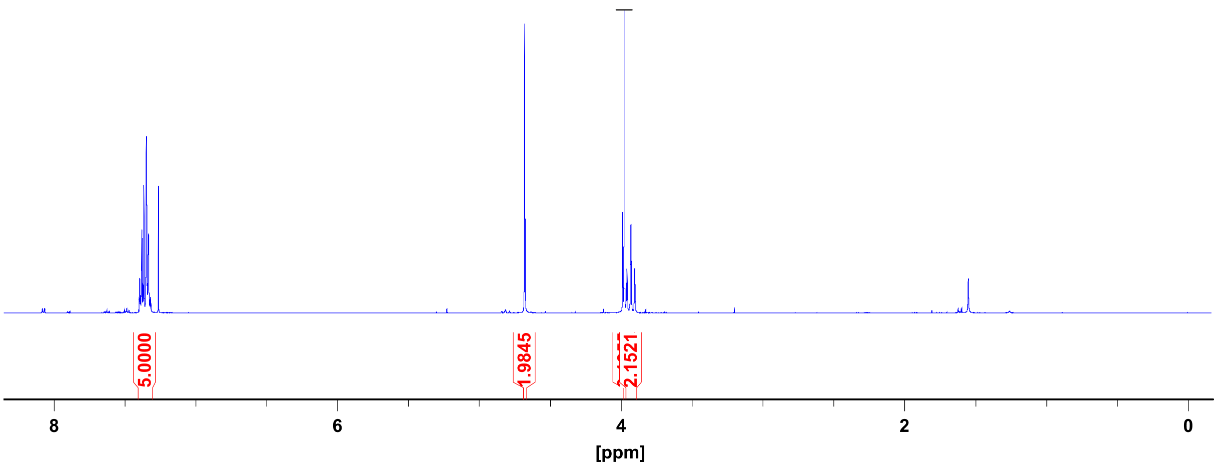

Figure S30: ^1H NMR spectrum of compound **11**, measured in $CDCl_3$ at 300 K. The additional signals in the aromatic range can presumably be assigned to by-product **38** (see below). Column chromatography did not separate the product from the by-product.

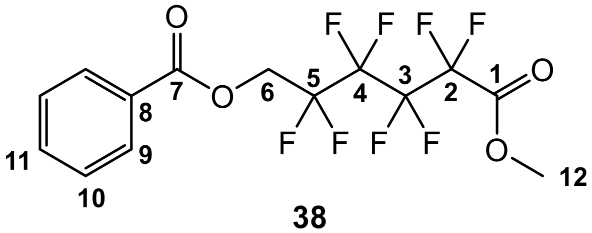

38

Figure S31: Since the by-product from the previous synthesis could not be removed, it was presumably also esterified in this step and formed the by-product **38**.

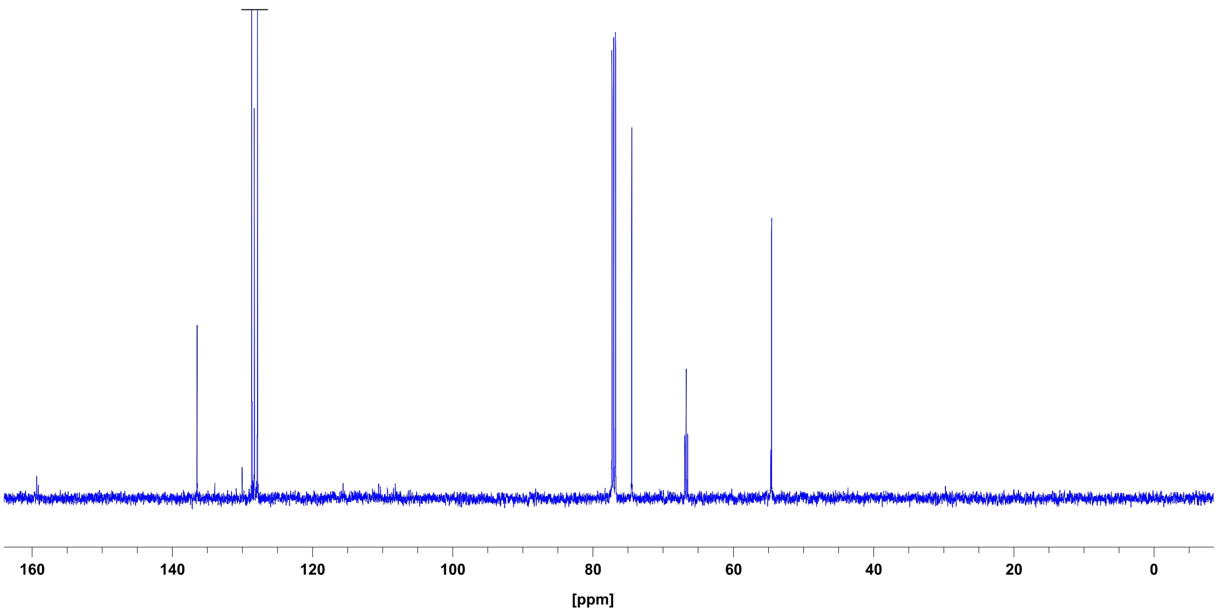

Figure S32: ^{13}C NMR spectrum of compound **11**, measured in $CDCl_3$ at 300 K. The additional signals can presumably be assigned to by-product **38** (see above). Column chromatography did not separate the product from the by-product.

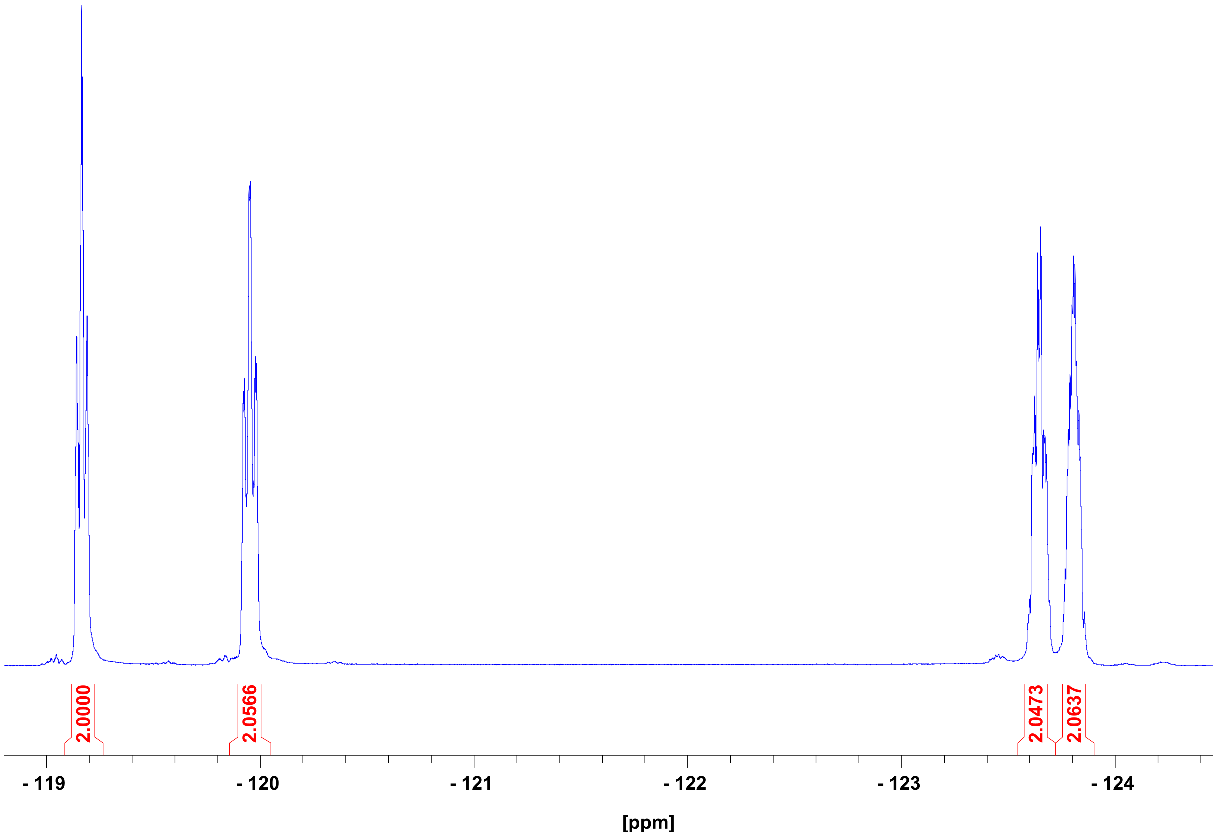

Figure S33: ^{19}F NMR spectrum of compound **11**, measured in $CDCl_3$ at 300 K. The

additional signals can presumably be assigned to by-product **38** (see above). Column chromatography did not separate the product from the by-product.

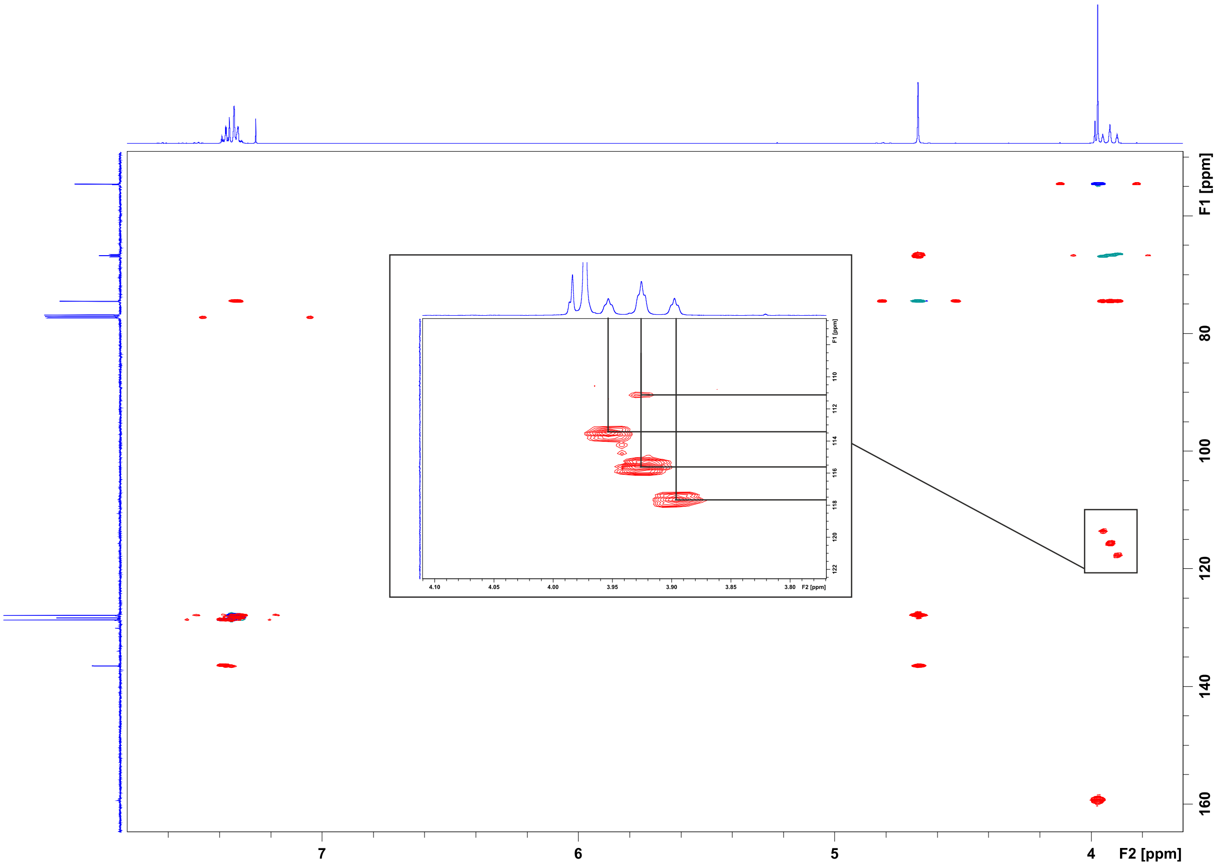

Figure S34: 2D spectra of compound **11**, showing the HSQC in blue/turquoise and the HMBC in red.

Methyl 8-benzyloxy-2,2,3,3,4,4,5,5,6,6,7,7-dodecafluormethyloctanoate (12)

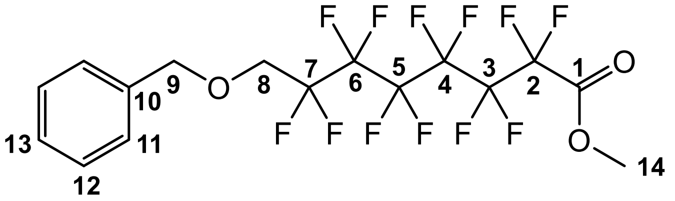

Yield (2 steps): 46 %.

^{1}H NMR (500 MHz, $CDCl_3$, 300 K): δ = 7.40-7.31 (m, 5 H, *H*-11, *H*-11', *H*-12, *H*-12', *H*-13), 4.68 (s, 2 H, *H*-9), 3.99 (s, 3 H, *H*-14), 3.94 (tt, ^{3}J (H-F) = 13.9 Hz, ^{4}J (H-F) = 1.4 Hz, 2 H, *H*-8) ppm.

^{13}C NMR (125 MHz, $CDCl_3$, 300 K): δ = 159.0 (t, ^{2}J (C-F) = 29.6 Hz, *C*-1), 136.4 (*C*-10), 128.6 (*C*-12), 128.3 (*C*-13), 127.8 (*C*-11), 117.7-113.6 (*C*-4-7), 74.4 (*C*-9), 66.7 (t, ^{2}J (C-F) = 25.3 Hz, *C*-8), 54.6 (*C*-14) ppm.

Due to the large number of ^{19}F-coupled C atoms, it was not possible to obtain sufficient signal intensity for the *C*-2 and *C*-3. For C-4-7 the range was determined by 2D spectra (see Figure 39).

^{19}F NMR (500 MHz, $CDCl_3$, 300 K): δ = -118.9 to -119.0 (m, 2 F, *F*-2), -119.8 to -119.9 (m, 2 F, *F*-7), -122.2 to -122.4 (m, 2 F, *F*-4), -122.5 to -122.7 (m, 2 F, *F*-5), -123.2 to -123.4 (m, 2 F, *F*-3), -123.7 to -123.9 (m, 2 F, *F*-6) ppm.

FT-IR: $\tilde{\nu}$ = 2965 (br, w), 2189 (w), 1784 (s), 1498 (w), 1456 (m), 1443 (m) 1323 (m), 1198 (s), 1138 (s), 1030 (w), 963 (w), 909 (w), 840 (w), 816 (w), 804 (w), 788 (w), 751 (m), 716 (w), 698 (m), 663 (m) cm^{-1}.

MS (EI, 70 eV): *m/z* (%) = 480 (22) $[M]^{+\cdot}$, 465.04 (4) $[M-CH_3]^{+\cdot}$, 91.06 (100) $[C_7H_7]^{+\cdot}$.

MS (EI, HR, 70 eV): $C_{16}H_{12}F_{12}O_3$ *m/z* = calc.: 480.05948, found: 480.05953, diff.: -0.09 ppm.

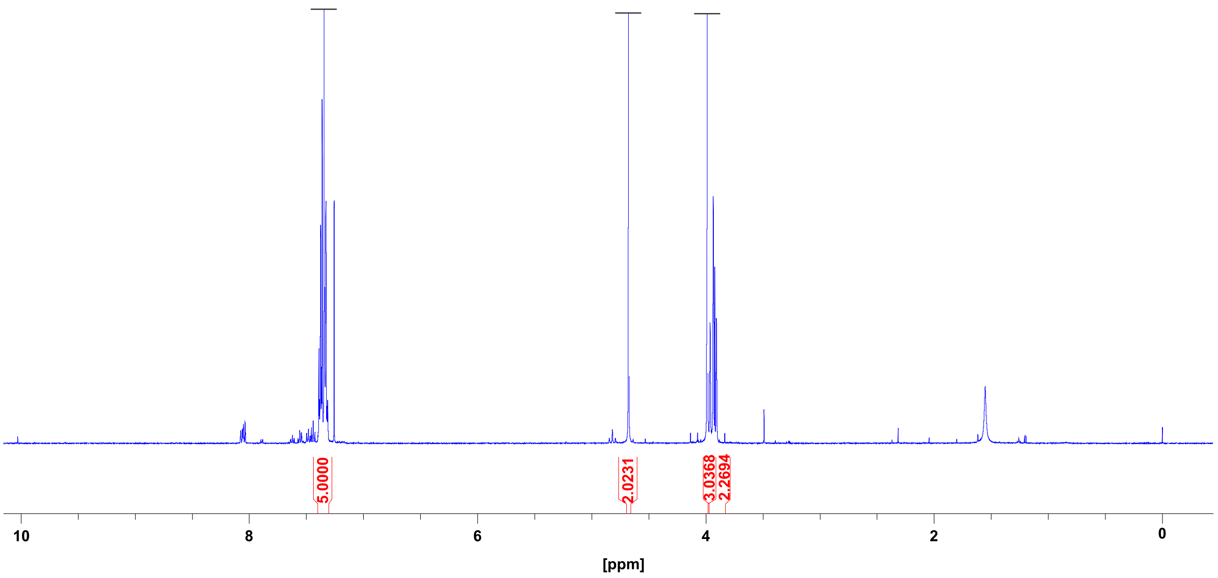

Figure S35: ^{1}H NMR spectrum of compound **12**, measured in $CDCl_3$ at 300 K. The additional signals in the aromatic range can presumably be assigned to by-product **39** (see below). Column chromatography did not separate the product from the by-product.

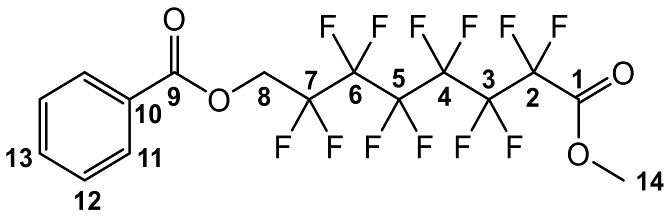

39

Figure S36: Since the by-product from the previous synthesis could not be removed, it was presumably also esterified in this step and formed the by-product **39**.

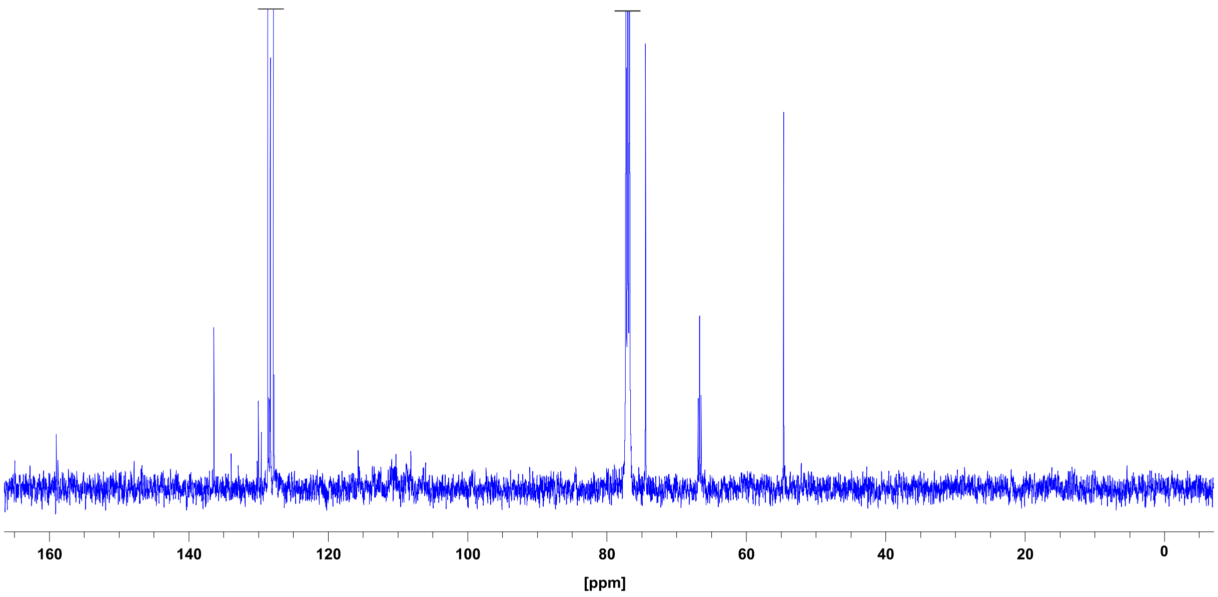

Figure S37: ^{13}C NMR spectrum of compound **12**, measured in $CDCl_3$ at 300 K. The additional signals can presumably be assigned to by-product **39** (see above). Column chromatography did not separate the product from the by-product.

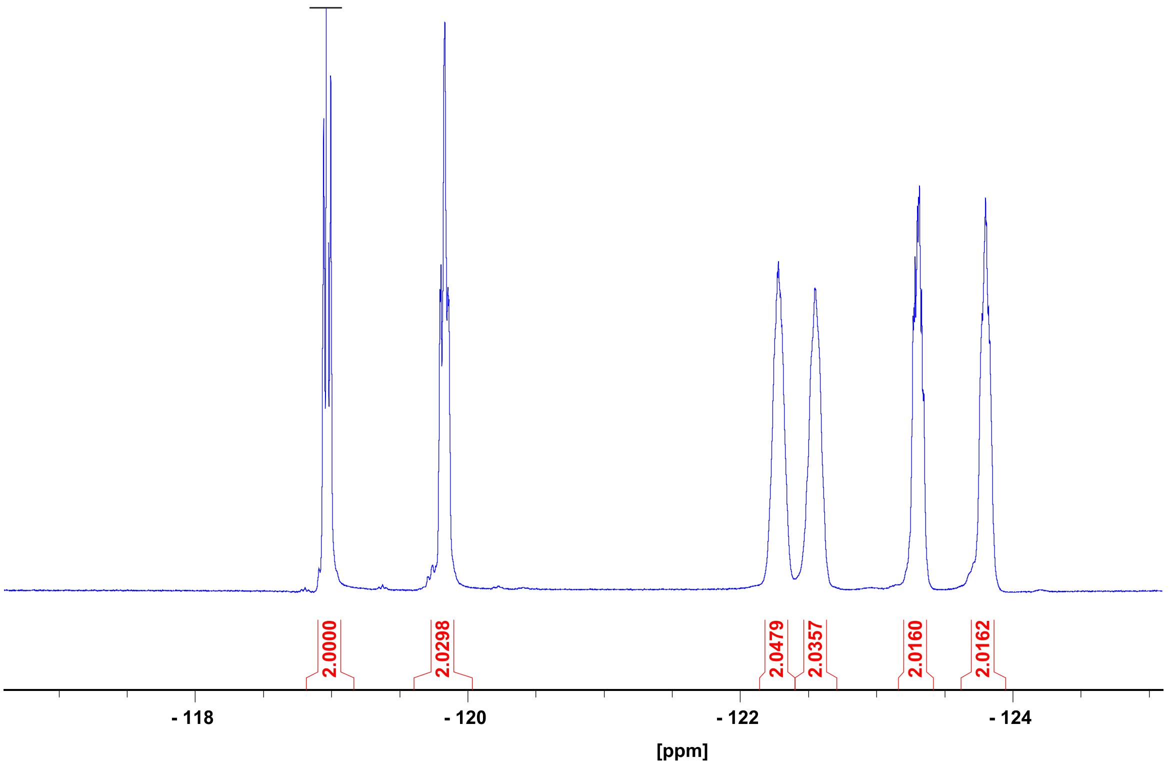

Figure S38: ^{19}F NMR spectrum of compound **12**, measured in $CDCl_3$ at 300 K.

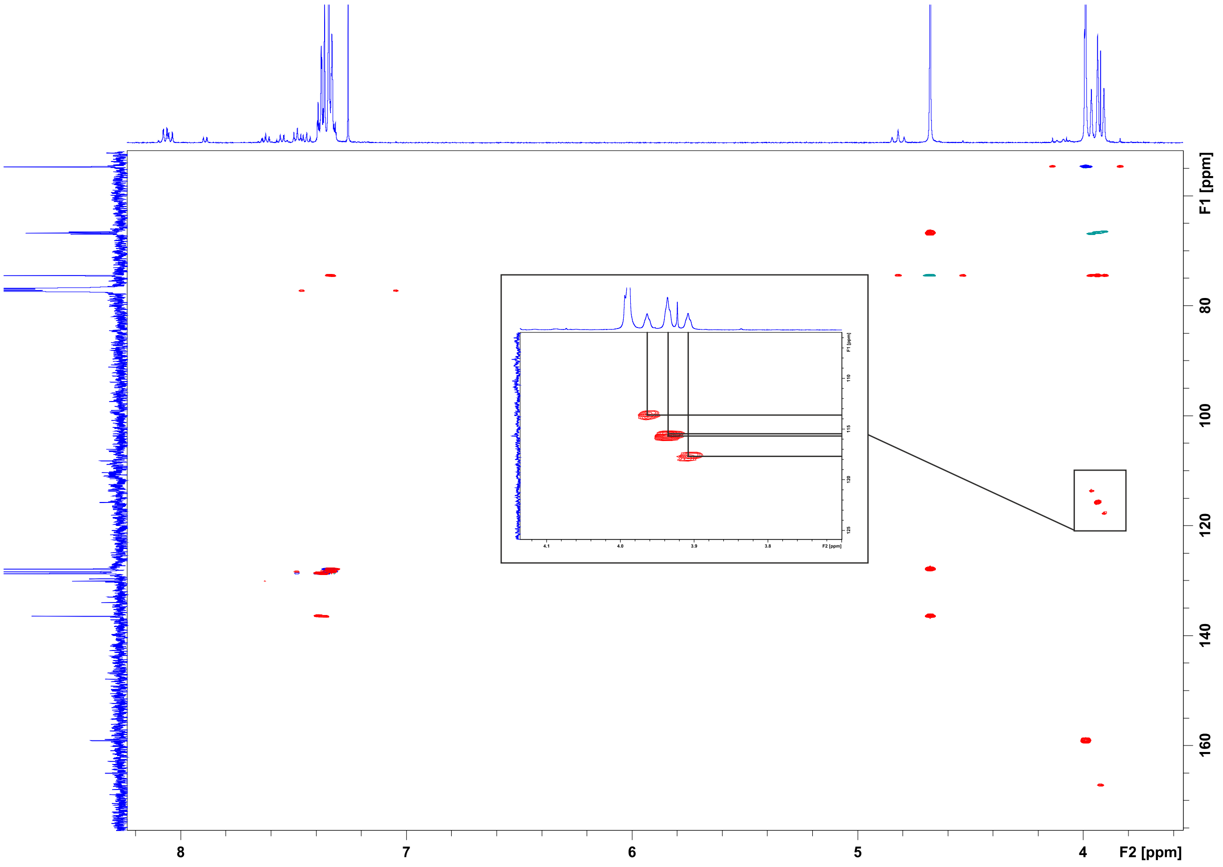

Figure S39: 2D spectra of compound **12**, showing the HSQC in blue/turquoise and the HMBC in red.

3.4 General procedure for the deprotection of 10, 11 and 12

Under a nitrogen atmosphere, the corresponding ester **10–12** (1 equiv) was dissolved in ethanol (300 mL) in a chicane flask and placed under a nitrogen atmosphere. In counter stream, palladium on activated carbon (10 mg, 10%) was added. The reaction solution was evacuated and placed under a hydrogen atmosphere; it was stirred vigorously for 24 h at room temperature. The catalyst was removed by filtration over Celite®545 and the solvent was removed in vacuo. A yellowish oil was obtained.

Methyl 4-hydroxy-2,2,3,3-tetrafluoromethylbutanoate (13)

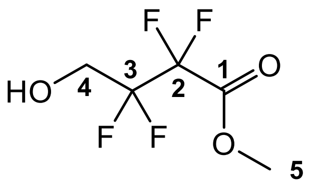

Yield: 85 %.

^{1}H NMR (500 MHz, $CDCl_3$, 300 K): δ = 4.07 (tt, ^3J (H-F) = 13.4 Hz, ^4J (H-F) = 1.4 Hz, 2 H, *H*-4), 3.96 (s, 3 H, *H*-5), 1.95 (broad s, 1H, *OH*) ppm.

^{13}C NMR (125 MHz, $CDCl_3$, 300 K): δ = 161.0 (t, ^2J (C-F) = 29.2, *C*-1), 117.3-113.4 (*C*-2-3), 60.5 (t, ^2J (C-F) = 25.9, *C*-4), 54.3 (*C*-5) ppm.

For C-2-3 the range was determined by 2D spectra (see Figure S44).

^{19}F NMR (500 MHz, $CDCl_3$, 300 K): δ = -121.5 (t, ^3J (H-F) = 1.8 Hz, 2 F, *F*-2), -123.9 (t, ^3J (H-F) = 1.8 Hz, 2 F, *F*-3) ppm.

FT-IR: $\tilde{\nu}$ = 3382 (br, w), 1771 (s), 1445 (w), 1335 (m), 1119 (s), 1078 (m), 1033 (m), 979 (w), 936 (w), 817 (w), 774 (w), 746 (w), 542 (w), 522 (w), 493 (w), 473 (w), 454 (w), 433 (w), 416 (w), 403 (m) cm^{-1}.

MS (EI, 70 eV): *m/z* (%) = no result.

MS (ESI): *m/z* (%) = no result.

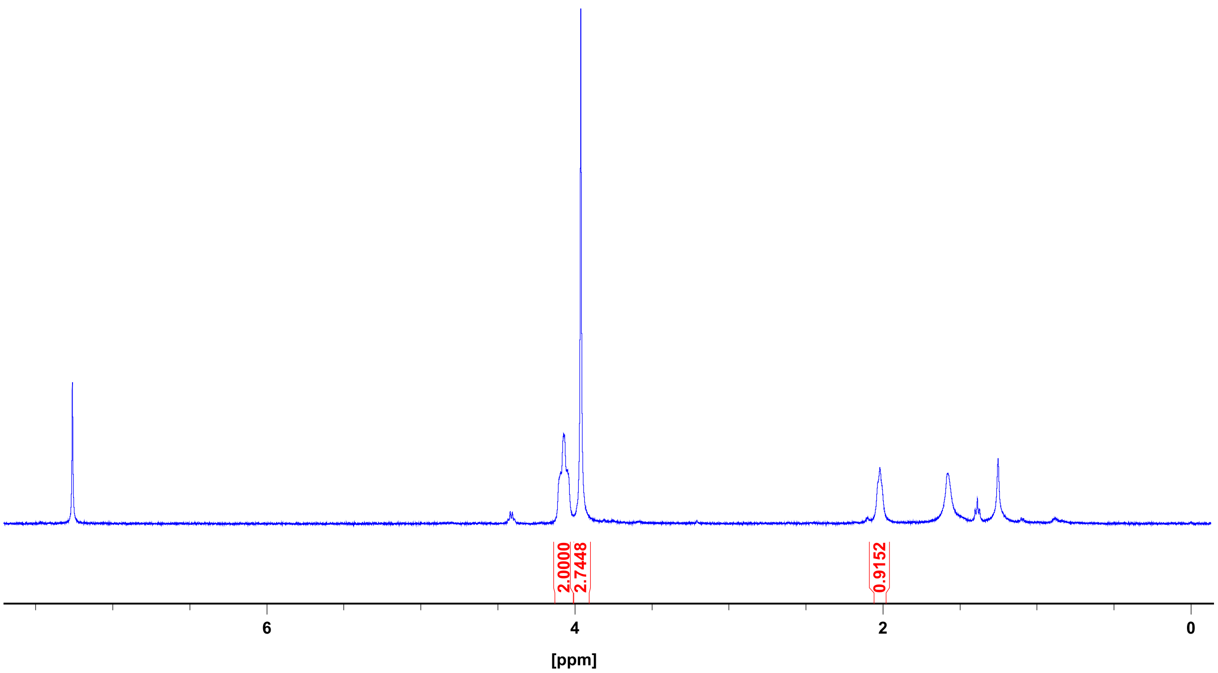

Figure S40: ^{1}H NMR spectrum of compound **13**, measured in $CDCl_3$ at 300 K.

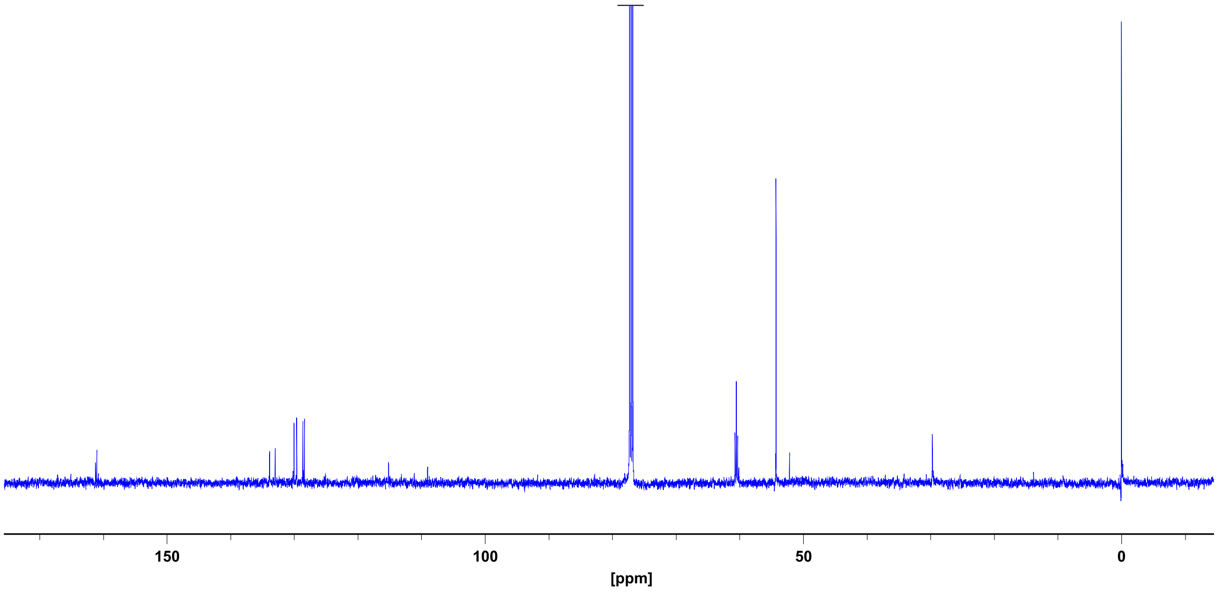

Figure S41: ^{13}C NMR spectrum of compound **13**, measured in $CDCl_3$ at 300 K. The additional signals can be assigned to side product **40** (see below).

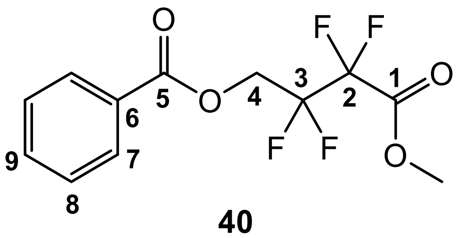

40

Figure S42: The by-product from the previous synthesis could not be hydrogenolytically deprotected in this step, which is probably why it remained as by-product **40**.

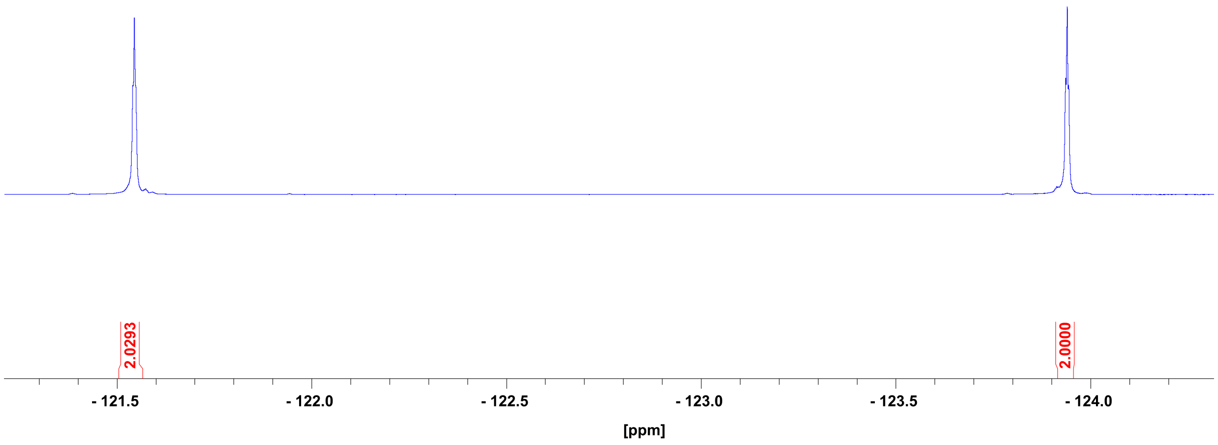

Figure S43: ^{19}F NMR spectrum of compound **13**, measured in $CDCl_3$ at 300 K.

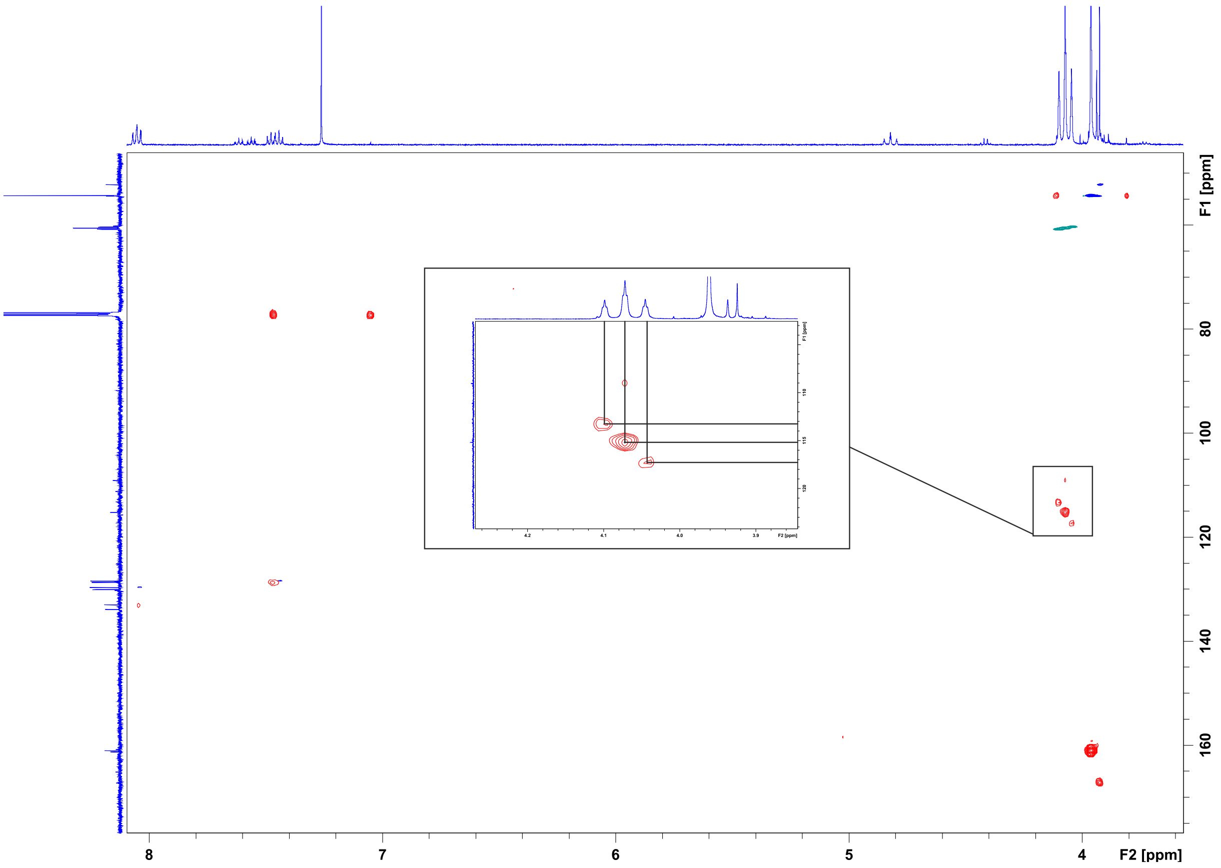

Figure S44: 2D spectra of compound **13**, showing the HSQC in blue/turquoise and the HMBC in red.

Methyl 6-hydroxy-2,2,3,3,4,4,5,5-octafluoromethylhexanoate (14)

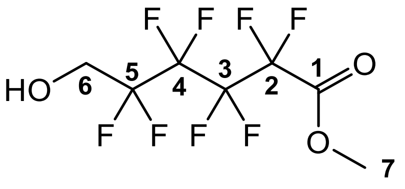

Yield: 65 %.

^{1}H NMR (500 MHz, $CDCl_3$, 300 K): δ = 4.08 (tt, ^3J (H-F) = 14.4 Hz, ^4J (H-F) = 1.5 Hz, 2 H, *H*-6), 3.99 (s, 3 H, *H*-7), 2.01(s, 1 H, *OH*) ppm.

^{13}C NMR (125 MHz, $CDCl_3$, 300 K): δ = 159.3 (t, ^{2}J (C-F) = 29.7, *C*-1), 117.5-113.2 (*C*-3-5), 60.6 (t, ^{2}J (C-F) = 25.5, *C*-6), 54.5 (*C*-7) ppm.

Due to the large number of ^{19}F-coupled C atoms, it was not possible to obtain sufficient signal intensity for the *C*-2. For C-3-5 the range was determined by 2D spectra (see Figure S48).

^{19}F NMR (500 MHz, $CDCl_3$, 300 K): δ = -119.1 to -119.2 (m, 2 F, *F*-2), -123.0 to -123.1 (m, 2 F, *F*-5), -123.6 to -123.7 (m, 2 F, *F*-3), -124.1 to -124.2 (m, 2 F, *F*-4) ppm.

FT-IR: $\tilde{\nu}$ = 2968 (w), 1779 (s), 1444 (m), 1325 (m), 1270 (m), 1179 (s), 1139 (s), 1045 (w), 1014 (w), 941 (m), 867 (m), 854 (w), 833 (w), 808 (m), 755 (m), 712 (m), 651 (w), 617 (w) cm^{-1}.

MS (ESI): *m/z* (%) = 324.99 (100) [M+Cl]$^{-}$.

MS (ESI, HR): $C_7H_6F_8O_3Cl$ *m/z* = calc.: 324.98832, found: 324.98888 diff.: -1.73 ppm.

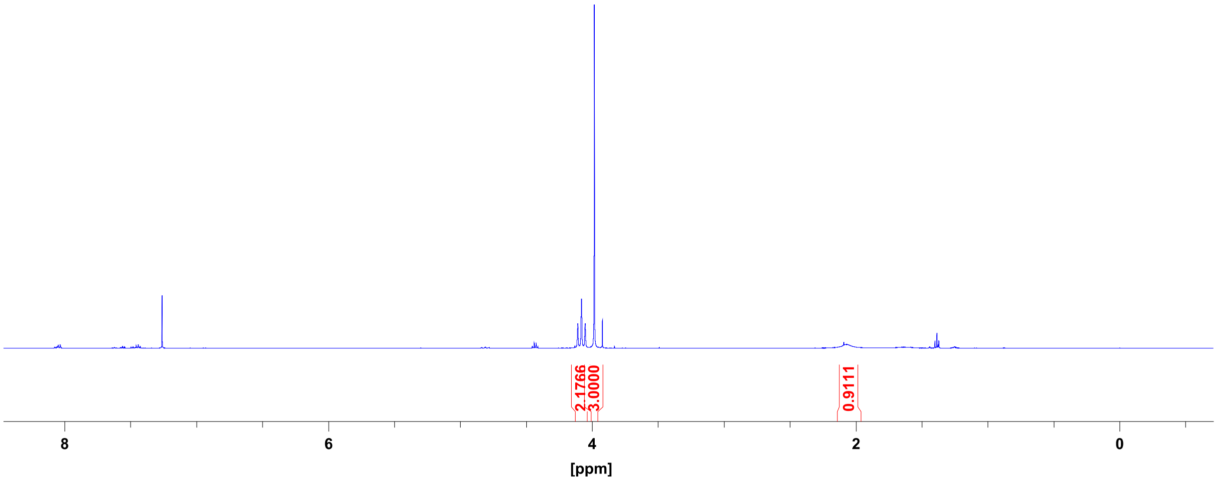

Figure S45: ^{1}H NMR spectrum of compound **14**, measured in $CDCl_3$ at 300 K. The additional signals in the aromatic range can presumably be assigned to by-product **38** (see above).

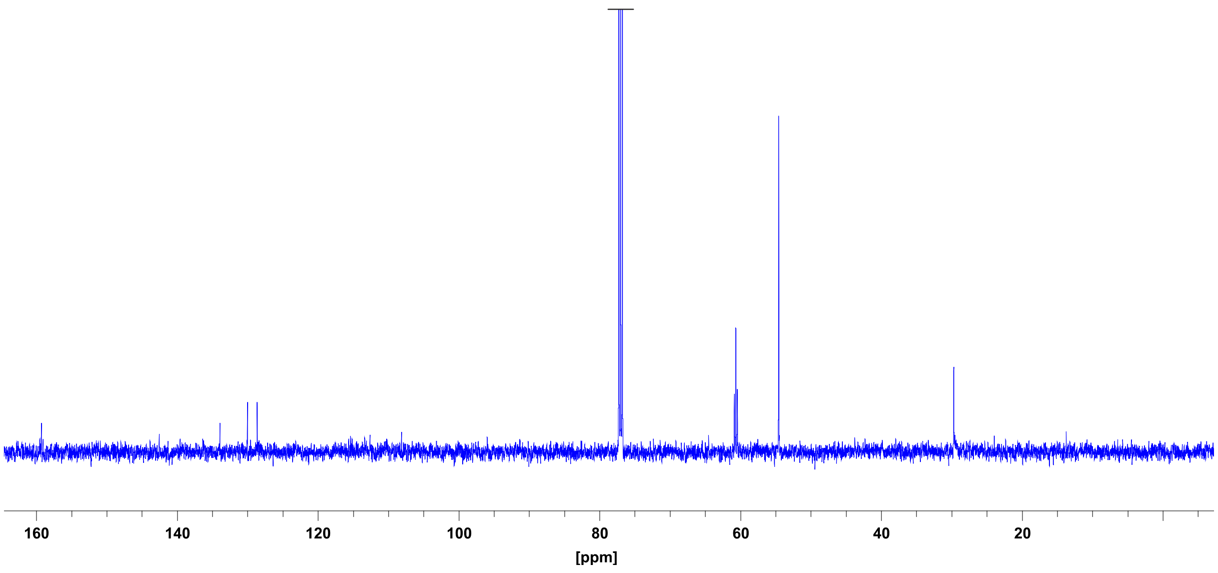

Figure S46: ^{13}C NMR spectrum of compound **14**, measured in $CDCl_3$ at 300 K. The additional signals can presumably be assigned to by-product **38** (see above).

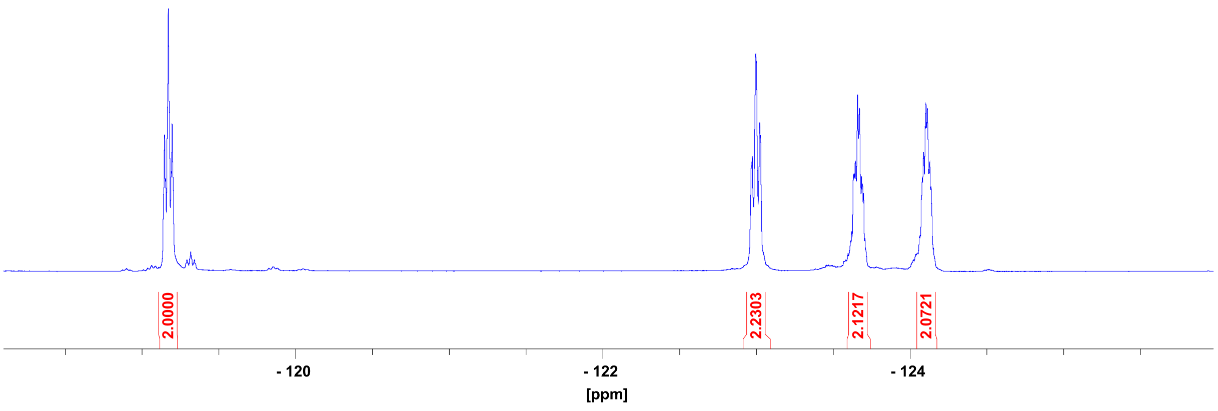

Figure S47: ^{19}F NMR spectrum of compound **14**, measured in $CDCl_3$ at 300 K. The additional signals can presumably be assigned to by-product **38** (see above).

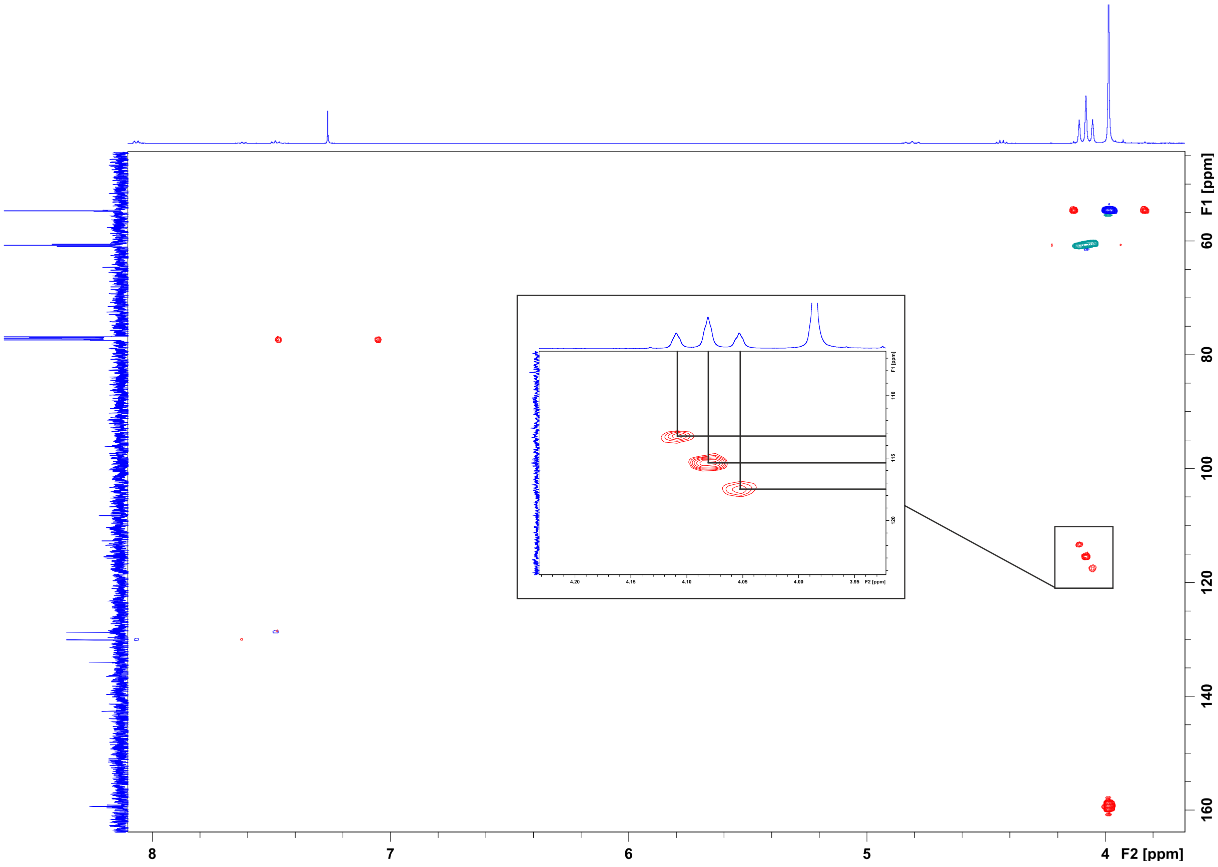

Figure S48: 2D spectra of compound **14**, showing the HSQC in blue/turquoise and the HMBC in red.

Methyl 8-hydroxy-2,2,3,3,4,4,5,5,6,6,7,7-dodecafluormethyloctanoate (15)

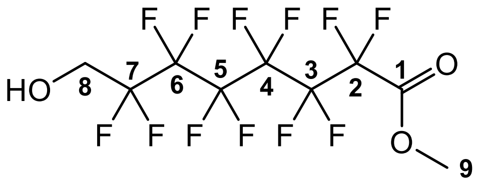

Yield: 99 %.

^{1}H NMR (500 MHz, $CDCl_3$, 300 K, TMS): δ = 4.09 (tt, ^3J (H-F) = 14.2 Hz, ^4J (H-F) = 1.4 Hz, 2 H, *H*-8), 4.00 (s, 3 H, *H*-9), 1.93 (s, 1 H, *OH*) ppm.

^{13}C NMR (125 MHz, $CDCl_3$, 300 K, TMS): δ = 159.0 (t, ^2J (C-F) = 29.5, *C*-1), 117.6-111.0 (*C*-4-7), 60.7 (t, ^2J (C-F) = 26.2, *C*-8), 54.6 (*C*-9) ppm.

Due to the large number of ^{19}F-coupled C atoms, it was not possible to obtain sufficient signal intensity for the *C*-2 and *C*-3. For C-4-7 the range was determined by 2D spectra (see Figure S52).

^{19}F NMR (500 MHz, $CDCl_3$, 300 K): δ = -118.9 to -119.0 (m, 2 F, *F*-2), -122.2 to 122.4 (m, 2 F, *F*-4), -122.5 to -122.7 (m, 2 F, *F*-5), -122.9 to -123.0 (m, 2 F, *F*-7), -123.2 to -123.4 (m, 2 F, *F*-3), -124.0 to -124.2 (m, 2 F, *F*-6) ppm.

FT-IR: $\tilde{\nu}$ = 3376 (w), 2921 (w), 2851 (w), 1752 (w), 1444 (w), 1348 (w), 1276 (w), 1257 (w), 1195 (m), 1173 (m), 1146 (m), 1135 (m), 1118 (m), 1083 (w), 1026 (w), 988 (w), 764 (w), 748 (w), 729 (w), 715 (w), 674 (w), 646 (w), 626 (w), 572 (w), 534 (m), 516 (w), 409 (w) cm^{-1}.

MS (EI, 70 eV): *m/z* (%) = 391.02 (13) $[M+H]^{+\cdot}$.

MS (EI, HR, 70 eV): $C_9H_7F_{12}O_3$ *m/z* = calc.: 391.02036, found: 391.02059, diff.: -0.60 ppm.

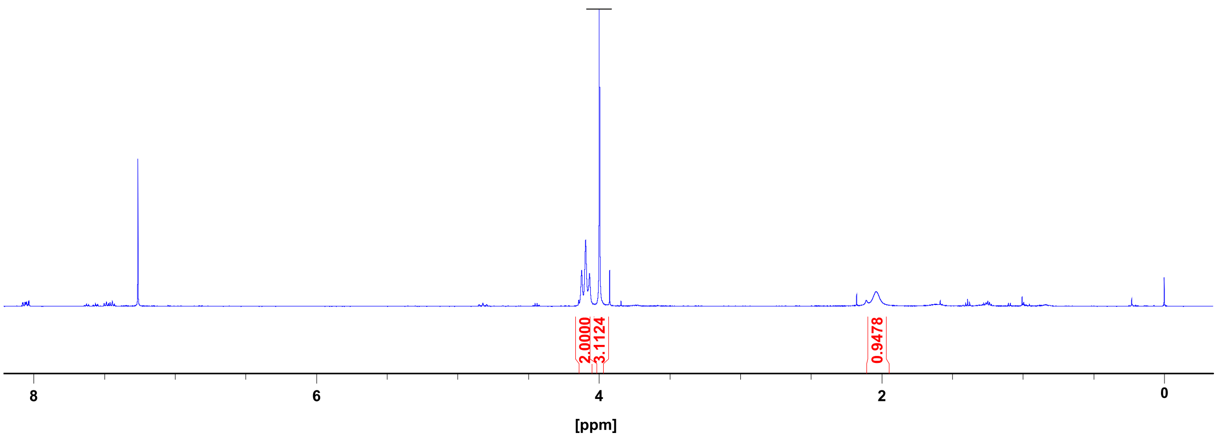

Figure S49: ^{1}H NMR spectrum of compound **15**, measured in $CDCl_3$ at 300 K. The additional signals in the aromatic range can presumably be assigned to by-product **39** (see above).

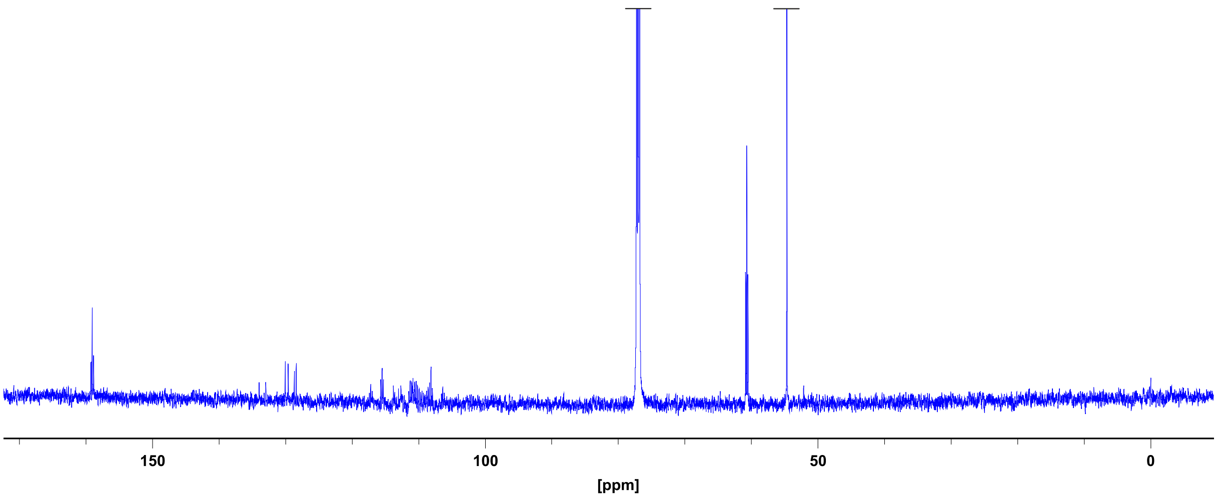

Figure S50: ^{13}C NMR spectrum of compound **15**, measured in $CDCl_3$ at 300 K. The additional signals can presumably be assigned to by-product **39** (see above).

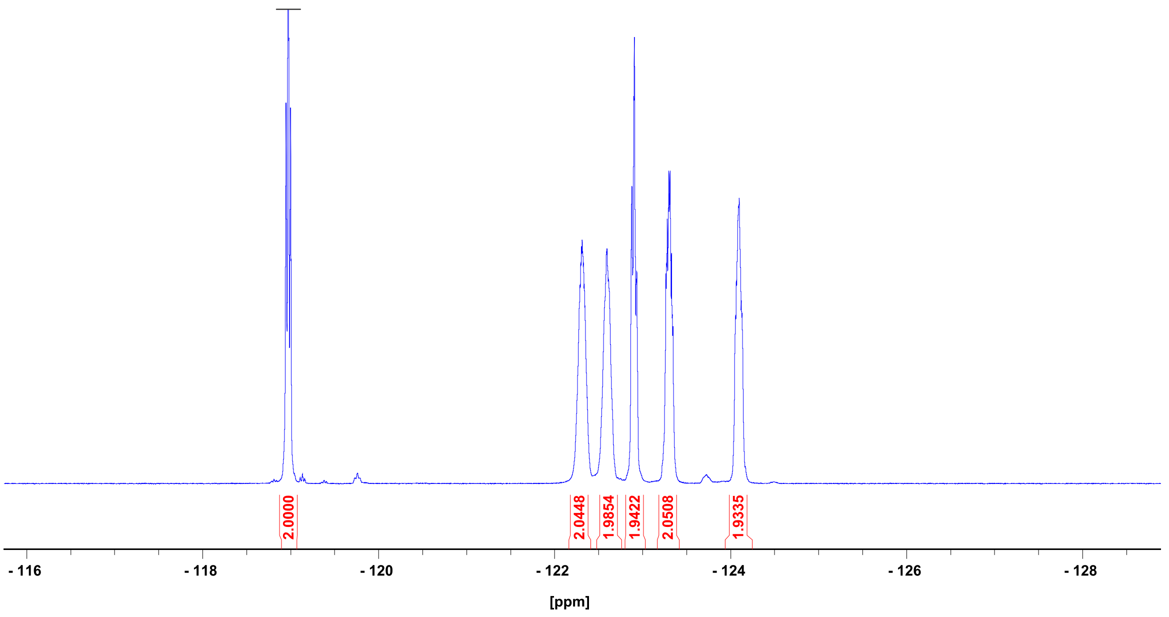

Figure S51: ^{19}F NMR spectrum of compound **15**, measured in $CDCl_3$ at 300 K. The additional signals can presumably be assigned to by-product **39** (see above).

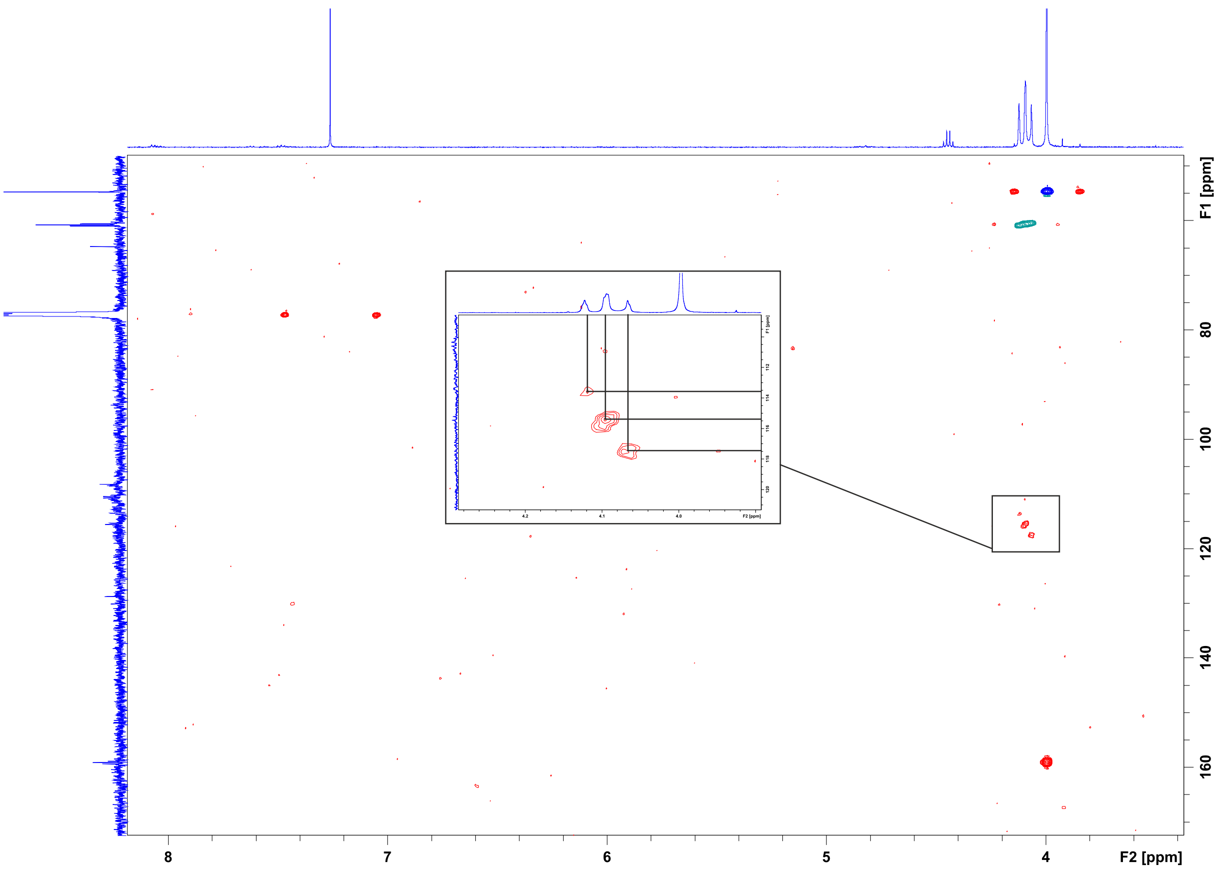

Figure S52: 2D spectra of compound **15**, showing the HSQC in blue/turquoise and the HMBC in red.

3.5 General procedure for the triflation of 13, 14 and 15

The corresponding alcohol **13–15** (1 equiv) and pyridine (1 equiv) were dissolved in dichloromethane (25 mL), the reaction solution was stirred at room temperature for 30 min. A seperate solution was prepared from trifluoromethanesulfonic anhydride (2 equiv) and dichloromethane (10 mL), which was added to the reaction solution over the course of one hour. After stirring for 18 h at room temperature, the solution was washed with dist. water (3 × 50 mL). The organic phase was dried over magnesium sulfate, filtered and the solvent was removed in vacuo. The crude product was then purified by flash column chromatography (cyclohexane/ethyl acetate, gradient: 5%–60%). A yellowish oil was obtained.

Methyl 2,2,3,3-tetrafluoro-4-(trifluoromethanesulfonyloxy)butanoate (16)

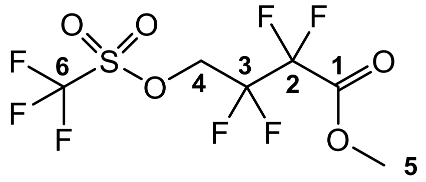

Yield: 28 %.

^{1}H NMR (500 MHz, $CDCl_3$, 300 K, TMS): δ = 4.88 (t, ^3J (H-F) = 12.8 Hz, 2 H, *H*-4), 4.00 (s, 3 H, *H*-5) ppm.

^{13}C NMR (125 MHz, $CDCl_3$, 300 K, TMS): δ = 159.8 (t, ^2J (C-F) = 29.9 Hz, *C*-1), 120.0-119.0 (q, *C*-6), 114.9-110.5 (*C*-2-3), 68.6 (t, ^2J (C-F) = 27.1 Hz, *C*-4), 54.7 (*C*-5) ppm.

For C-2-3 the range was determined by 2D spectra (see Figure S56).

^{19}F NMR (500 MHz, $CDCl_3$, 300 K): δ = -74.5 (t, ^3J (H-F) = 1.4 Hz, 3 F, *F*-6), -120.3 (t, ^3J (H-F) = 1.3 Hz, 2 F, *F*-3), -121.7 (s, 2 F, *F*-2) ppm.

FT-IR: $\tilde{\nu}$ = 2970 (w), 1773 (m), 1425 (m), 1338 (m), 1272 (w), 1250 (m), 1241 (s), 1136 (s), 1046 (m), 1011 (m), 984 (m), 953 (m), 922 (w), 815 (m), 751 (w), 713 (w), 610 (m) cm^{-1}.

MS (EI, 70 eV): *m/z* (%) = no result.

MS (ESI): *m/z* (%) = no result.

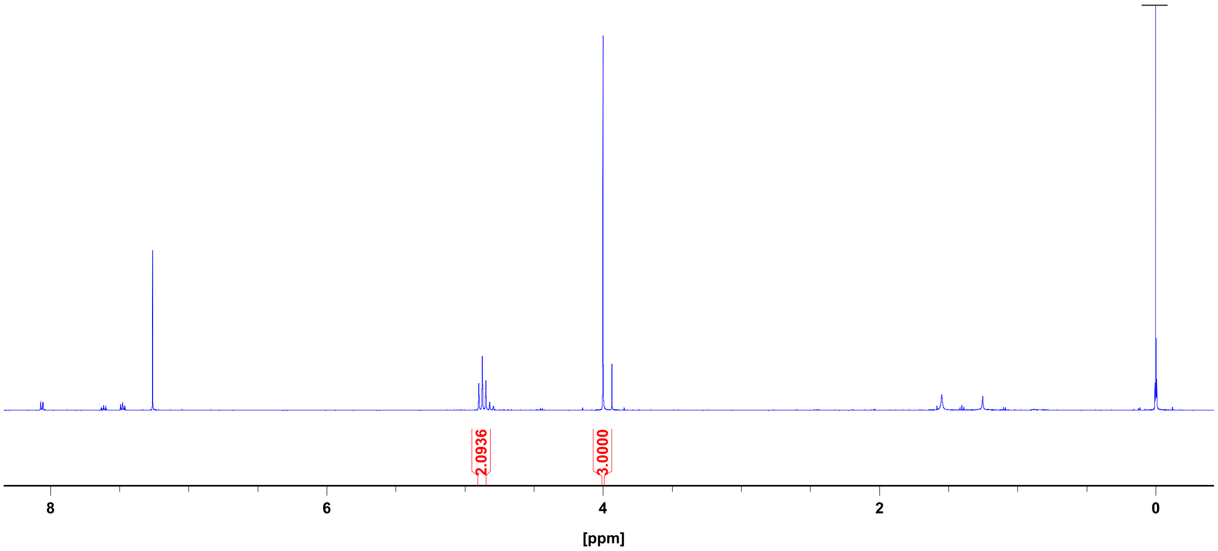

Figure S53: ^{1}H NMR spectrum of compound **16**, measured in $CDCl_3$ at 300 K. The additional signals in the aromatic range can presumably be assigned to by-product **40** (see above). Column chromatography did not separate the product from the by-product.

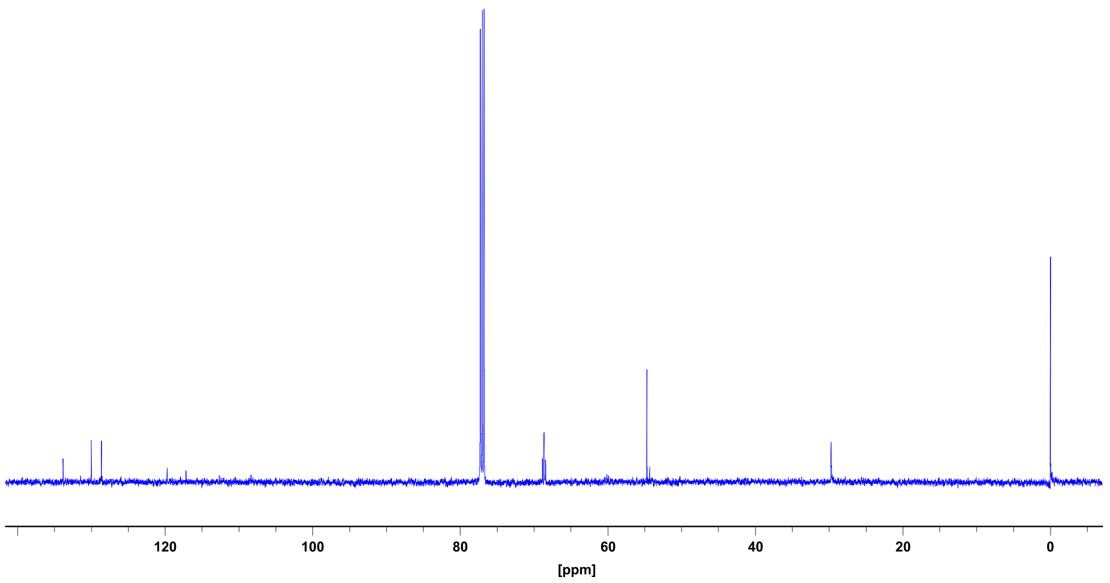

Figure S54: ^{13}C NMR spectrum of compound **16**, measured in $CDCl_3$ at 300 K. The additional signals can presumably be assigned to by-product **40** (see above). Column chromatography did not separate the product from the by-product.

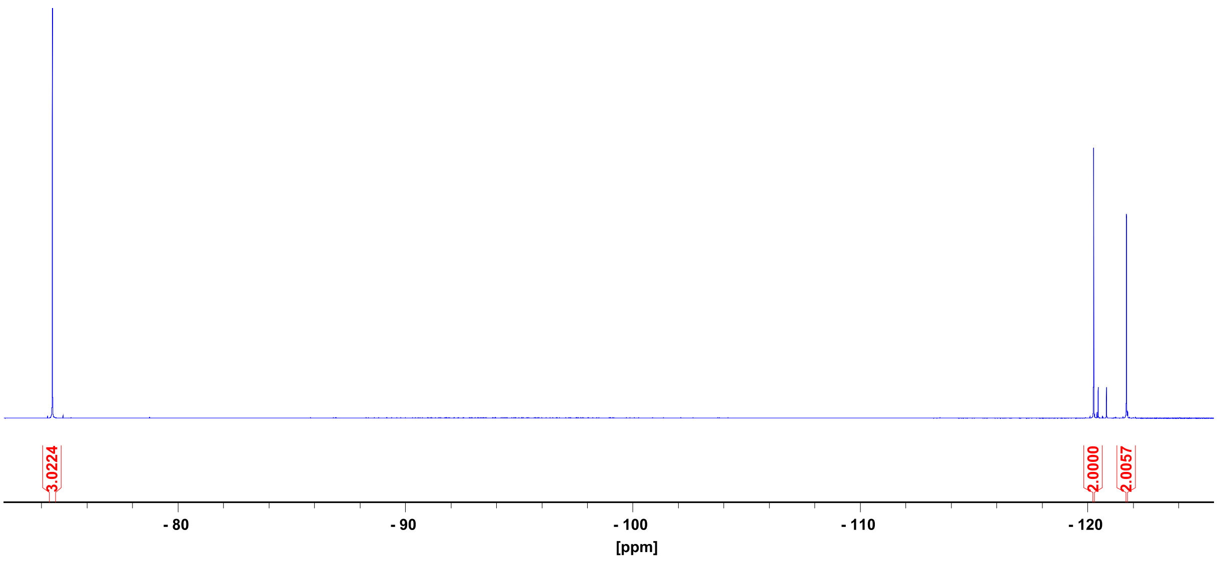

Figure S55: ^{19}F NMR spectrum of compound **16**, measured in $CDCl_3$ at 300 K. The additional signals can presumably be assigned to by-product **40** (see above). Column chromatography did not separate the product from the by-product.

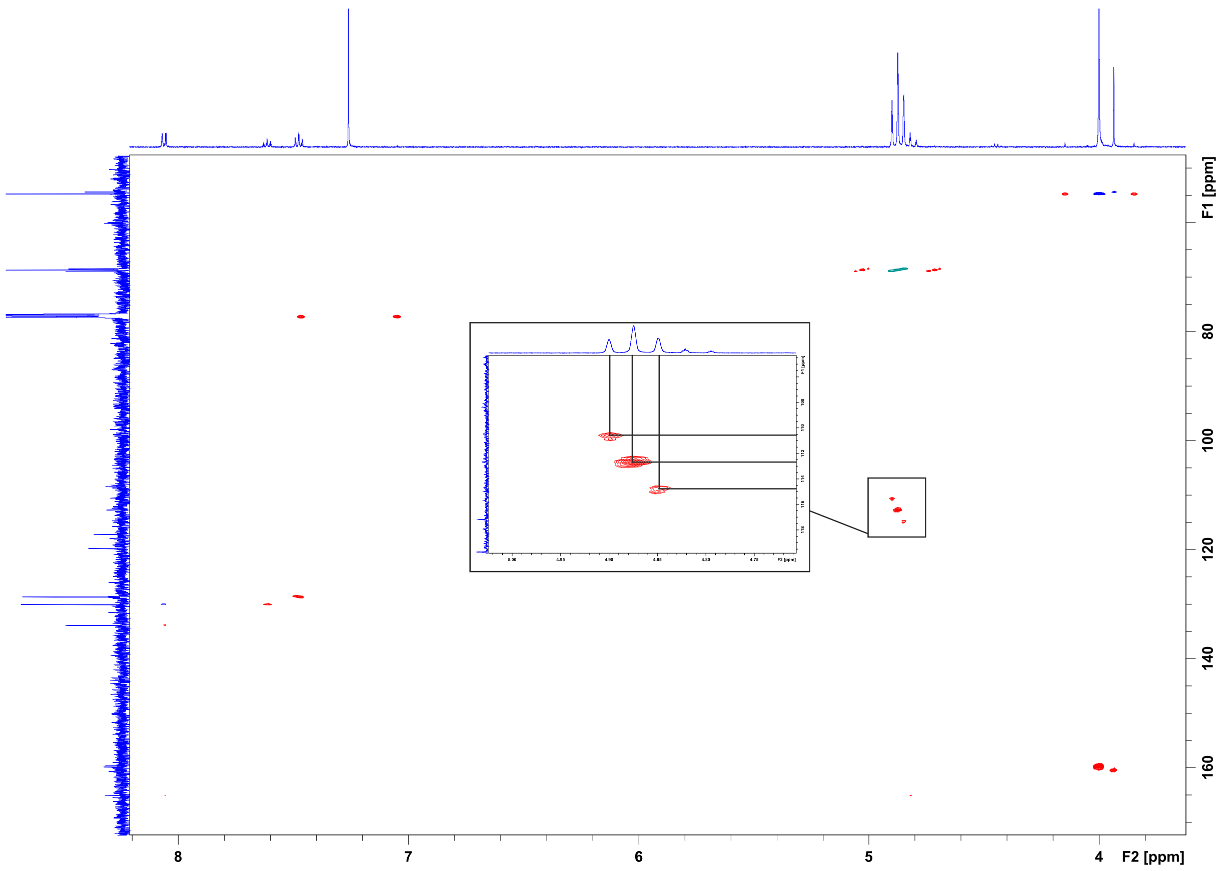

Figure S56: 2D spectra of compound **16**, showing the HSQC in blue/turquoise and the HMBC in red.

Methyl 2,2,3,3,4,4,5,5-octafluoro-6-(trifluoromethanesulfonyloxy)hexanoate (17)

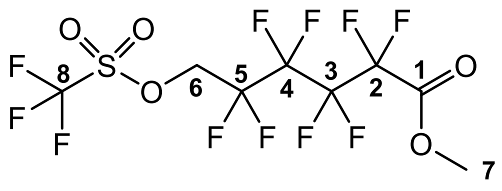

Yield: 41 %.

^{1}H NMR (500 MHz, $CDCl_3$, 300 K): δ = 4.81 (t, ^3J (H-F) = 12.5 Hz, 2 H, *H*-6), 4.00 (s, 3 H, *H*-7) ppm.

^{13}C NMR (125 MHz, $CDCl_3$, 300 K): δ = 158.8(t, ^{2}J (C-F) = 29.4 Hz, *C*-1), 120.0-119.0 (q, *C*-8), 115.0-110.6 (m, *C*-2-5), 68.2 (t, ^{2}J (C-F) = 27.8 Hz, *C*-6), 54.7 (*C*-7) ppm.

For C-2-5 the range was determined by 2D spectra (see Figure S60).

^{19}F NMR (500 MHz, $CDCl_3$, 300 K): δ = -74.4 (br. s, 3 F, *F*-8), -118.9 to -119.0 (m, 2 F, *F*-2), -120.3 to -120.4 (m, 2 F, *F*-5), -123.2 to -123.3 (m, 2 F, *F*-4), -123.5 to -123.6 (m, 2 F, *F*-3) ppm.

FT-IR: $\tilde{\nu}$ = 2969 (w), 1783 (m), 1718 (w), 1427 (m), 1324 (w), 1250 (w) 1190 (s), 1138 (s), 1037 (m), 1017 (m), 956 (m), 871 (w), 840 (m), 806 (s), 769 (m), 746 (w), 713 (m), 610 (s) cm^{-1}.

MS (ESI): *m/z* (%) = 456.94 (100) $[M+Cl]^-$.

MS (ESI, HR): $C_8H_5F_8O_5Cl$ *m/z* = calc.: 456.93761, found: 456.93860 diff.: -2.17 ppm.

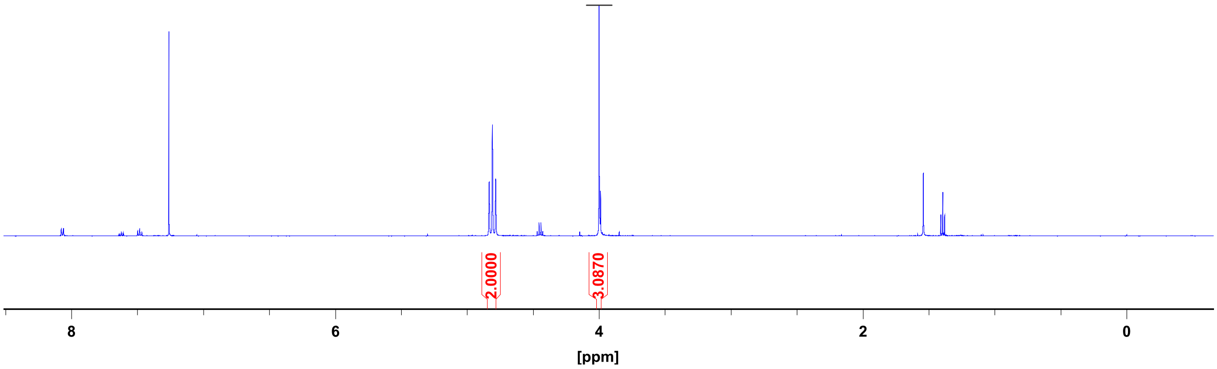

Figure S57: ^{1}H NMR spectrum of compound **17**, measured in $CDCl_3$ at 300 K. The additional signals in the aromatic range can presumably be assigned to by-product **38** (see above). Column chromatography did not separate the product from the by-product.

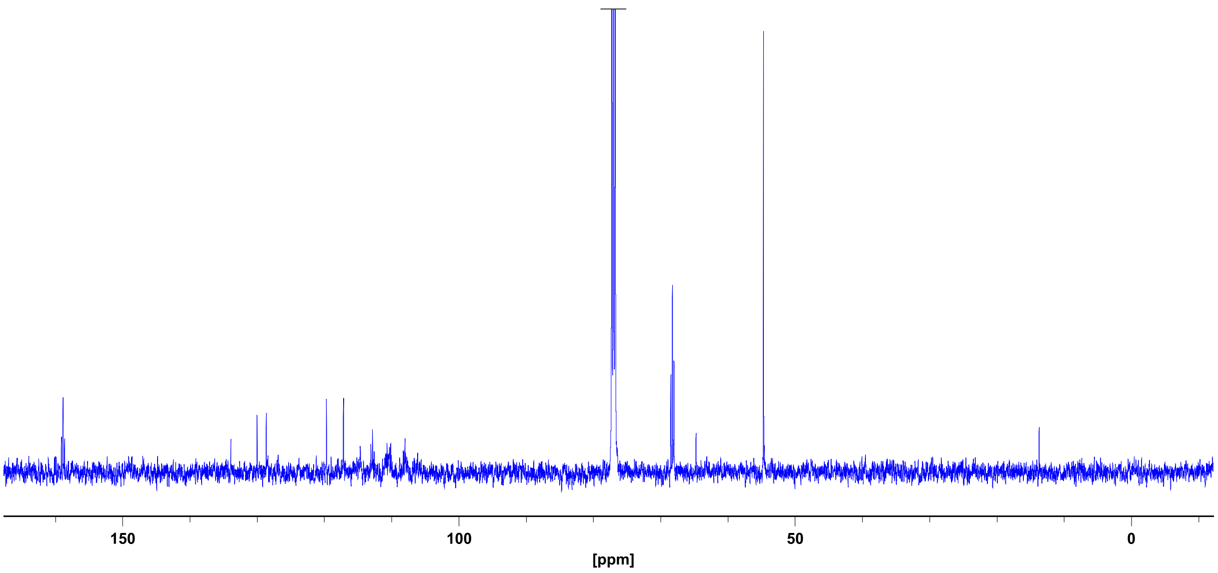

Figure S58: ^{13}C NMR spectrum of compound **17**, measured in $CDCl_3$ at 300 K. The additional signals can presumably be assigned to by-product **38** (see above). Column chromatography did not separate the product from the by-product.

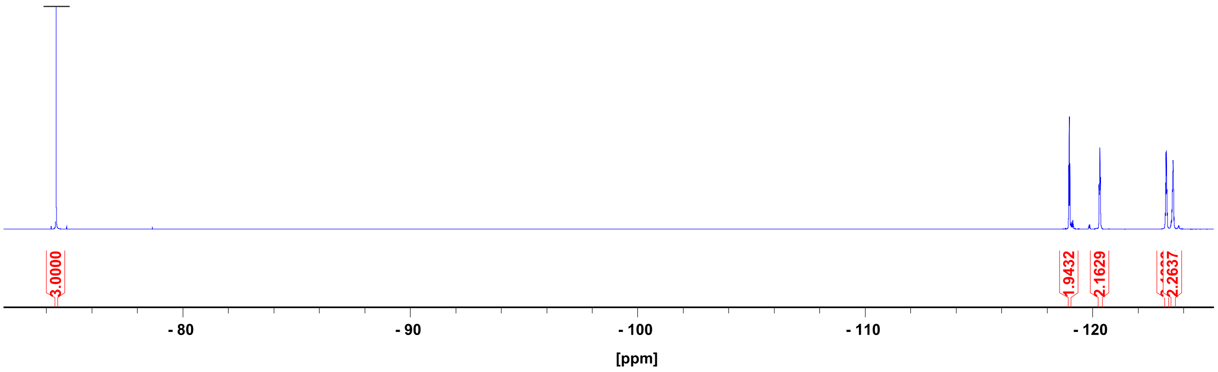

Figure S59: ^{19}F NMR spectrum of compound **17**, measured in $CDCl_3$ at 300 K. The additional signals can presumably be assigned to by-product **38** (see above). Column chromatography did not separate the product from the by-product.

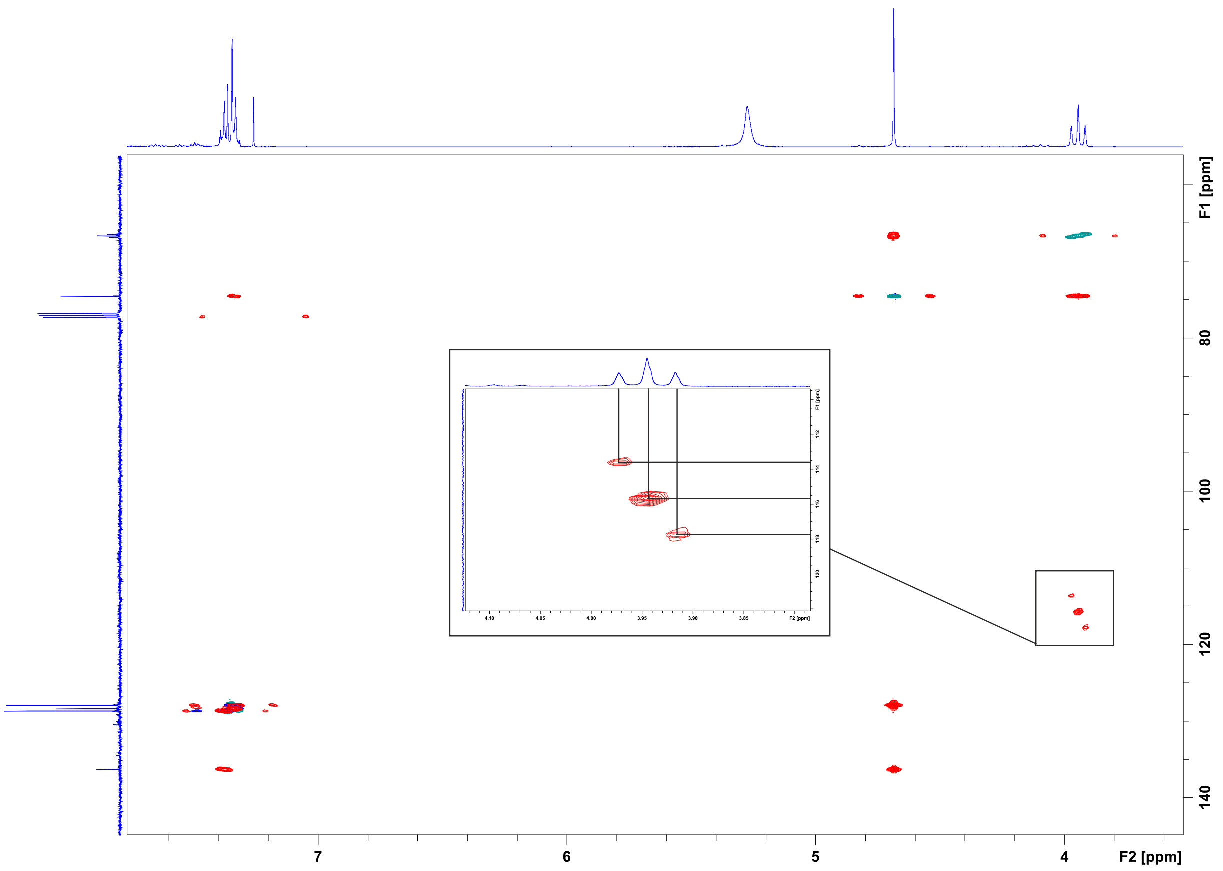

Figure S60: 2D spectra of compound **17**, showing the HSQC in blue/turquoise and the HMBC in red.

Methyl 2,2,3,3,4,4,5,5,6,6,7,7-dodecafluoro-8-(trifluoromethanesulfonyloxy)-octanoate (18)

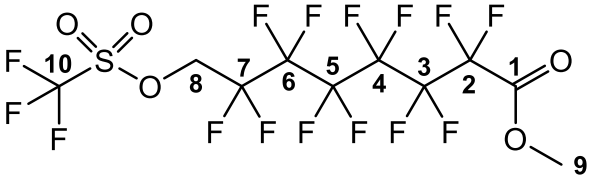

Yield: 63 %.

^{1}H NMR (500 MHz, $CDCl_3$, 300 K): δ = 4.82 (t, ^3J (H-F) = 12.3 Hz, 2 H, *H*-8), 4.00 (s, 3 H, *H*-9) ppm.

^{13}C NMR (125 MHz, $CDCl_3$, 300 K): δ = 158.8 (t, ^{2}J (C-F) = 29.4 Hz, *C*-1), 120.0-119.0 (q, ^{1}J (C-F) = 319.3 Hz, *C*-10), 115.0-110.9 (m, *C*-2-7), 68.1 (t, ^{2}J (C-F) = 28.2 Hz, *C*-8), 54.7 (*C*-9) ppm.

For C-2-7 the range was determined by 2D spectra (see Figure S64).

^{19}F NMR (500 MHz, $CDCl_3$, 300 K): δ = -74.5 (br. s, 3 F, *F*-10), -118.9 to -119.0 (m, 2 F, *F*-2), -120.1 to -120.2 (m, 2 F, *F*-7), -122.1 to -122.4 (m, 4 F, *F*-4, *F*-5), -123.1 to -123.3 (m, 2 F, *F*-6), -123.3 to -123.5 (m, 2 F, *F*-3) ppm.

FT-IR: ṽ = 2970 (w), 1785 (s), 1428 (s), 1325 (m), 1249 (w), 1199 (s) 1137 (s), 1015 (s), 958 (w), 904 (w), 816 (m), 763 (m), 716 (w), 691 (w), 657 (w), 610 (s) cm^{-1}.

MS (ESI): *m/z* (%) = 544.95 (100) $[M+Na]^{+\cdot}$.

MS (ESI, HR): $C_{10}H_5F_{15}O_5NaS$ *m/z* = calc.: 544.95104, found: 544.95095, diff.: -0.17 ppm.

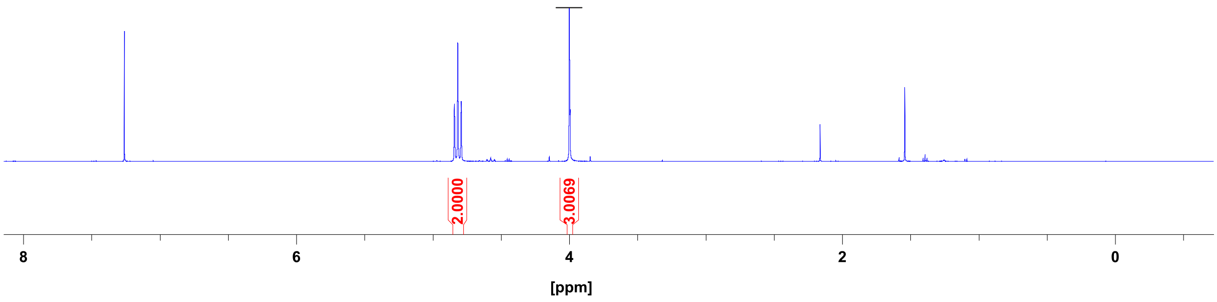

Figure S61: ^{1}H NMR spectrum of compound **18**, measured in $CDCl_3$ at 300 K.

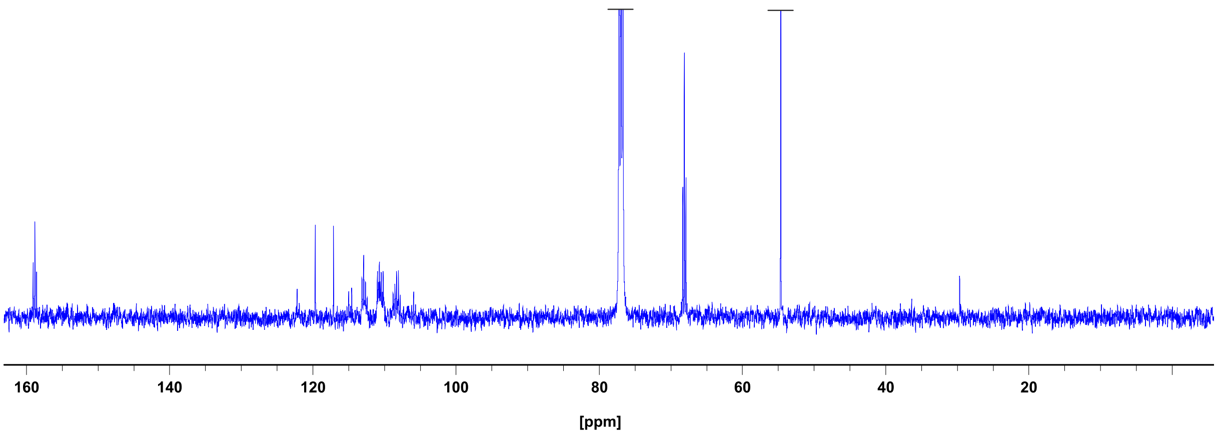

Figure S62: ^{13}C NMR spectrum of compound **18**, measured in $CDCl_3$ at 300 K.

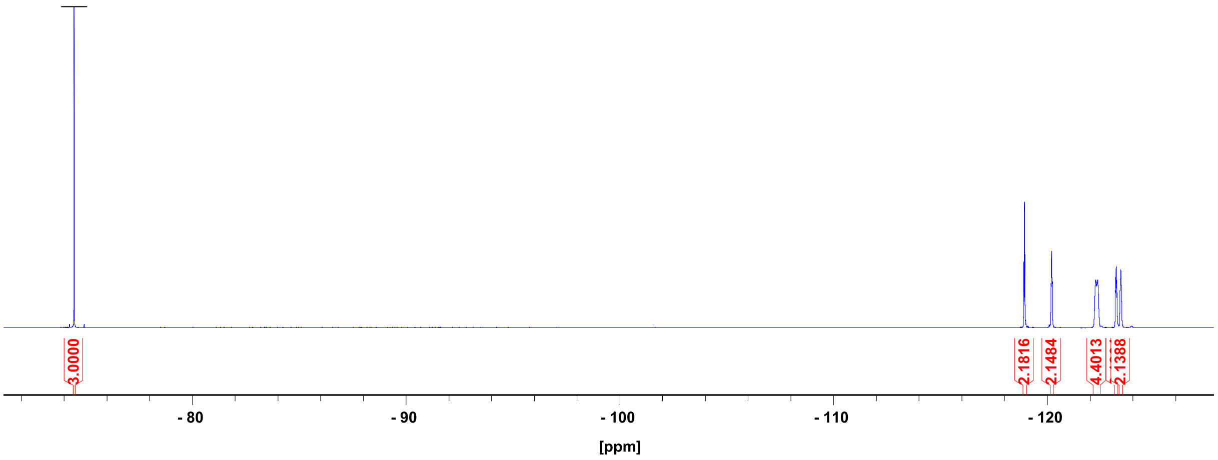

Figure S63: ^{19}F NMR spectrum of compound **18**, measured in $CDCl_3$ at 300 K.

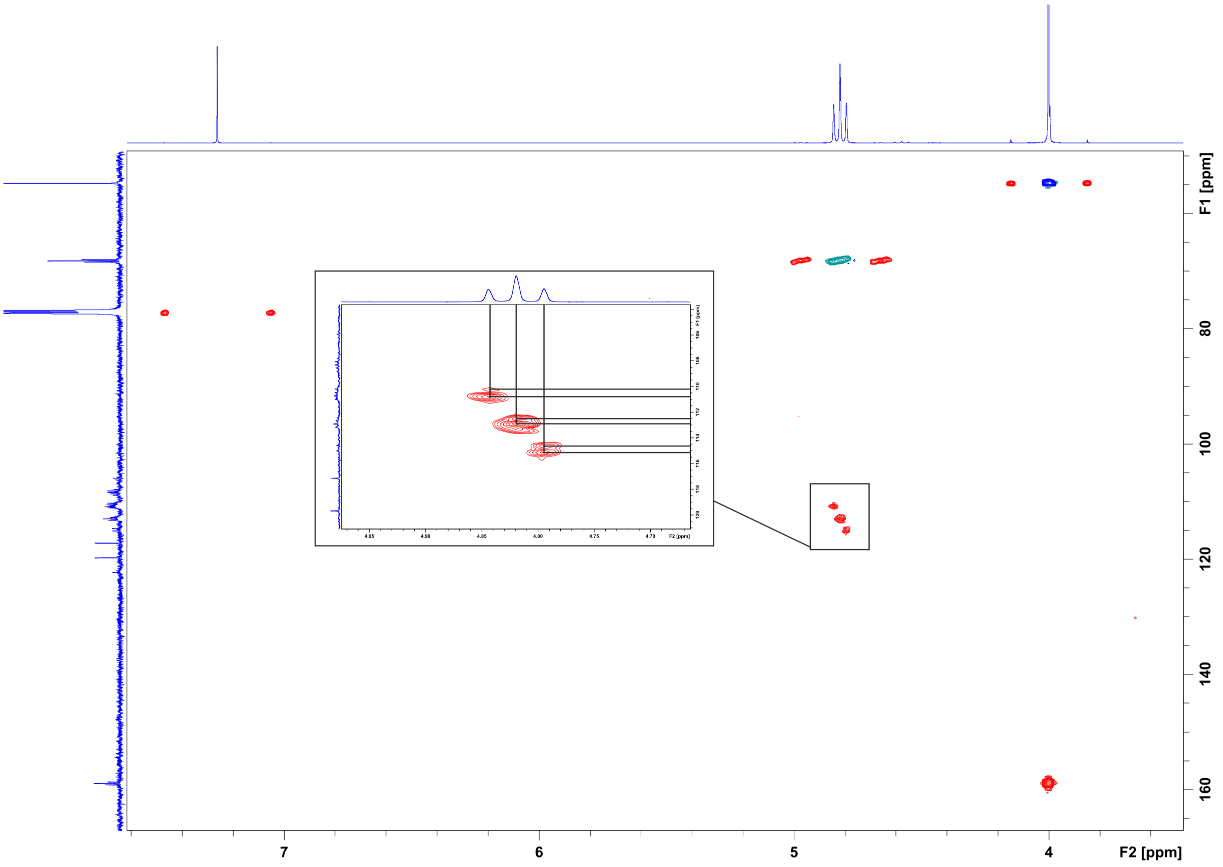

Figure S64: 2D spectra of compound **18**, showing the HSQC in blue/turquoise and the HMBC in red.

3.6 Synthesis of 2-iodo-3,4,5-trimethoxybenzaldehyde (20)

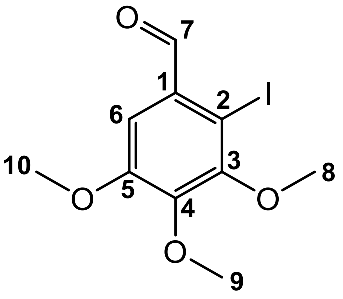

3,4,5-Trimethoxybenzaldehyde (3.00 g, 15.3 mmol) was dissolved in acetonitrile (20 mL), then *N*-iodosuccinimide (6.87 g, 30.6 mmol) and three drops of trifluoroacetic acid were added and the solution was refluxed for 18 h. After cooling the reaction

mixture, sodium sulfite (1.92 g, 15.3 mmol) was added and stirred for a further 2 h at room temperature. Then, dichloromethane (150 mL) was added and diluted with sodium chloride solution (2 × 50 mL) and dist. water (1 × 50 mL). The organic layer was dried over magnesium sulfate, filtered, the solvent removed in vacuo, and the crude product was purified by flash column chromatography (cyclohexane/ethyl acetate, gradient 5%–60% ethyl acetate). A yellow solid was obtained.

Yield: 4.39 g (13.7 mmol, 89 %) (Lit.[1]: 99 %).

Melting Point: 66.7 °C.

^{1}H NMR (500 MHz, $CDCl_3$, 300 K): δ = 10.05 (s, 1 H, *H*-7), 7.35 (s, 1 H, *H*-6), 3.97 (s, 3 H, *H*-9), 3.92 (s, 3 H, *H*-8), 3.90 (s, 3 H, *H*-10) ppm.

^{13}C NMR (125 MHz, $CDCl_3$, 300 K): δ = 195.3 (*C*-7), 154.0 (*C*-5), 153.0 (*C*-3), 147.8 (*C*-4), 130.6 (*C*-1), 108.6 (*C*-6), 91.5 (*C*-2), 61.2 (*C*-9), 61.0 (*C*-8), 56.3 (*C*-10) ppm.

FT-IR: $\tilde{\nu}$ = 2936 (m), 2853 (m), 2249 (w), 1685 (s), 1573 (m), 1424 (m), 1446 (m), 1398 (m), 1378 (s), 1320 (s), 1283 (m), 1242 (m), 1197 (s), 1161 (s), 1100 (s), 1041 (m), 997 (s), 978 (s), 920 (s), 862 (s), 808 (m), 773 (m), 750 (w), 722 (m), 663 (m), 637 (m), 577 (m), 522 (m) cm^{-1}.

MS (EI, 70 eV): *m/z* (%) = 321.97 (100) $[M]^{+\cdot}$.

MS (EI, HR, 70 eV): $C_{10}H_{11}O_4I$ *m/z* = calc.: 321.97020, found: 321.97020, diff.: 0.00 ppm.

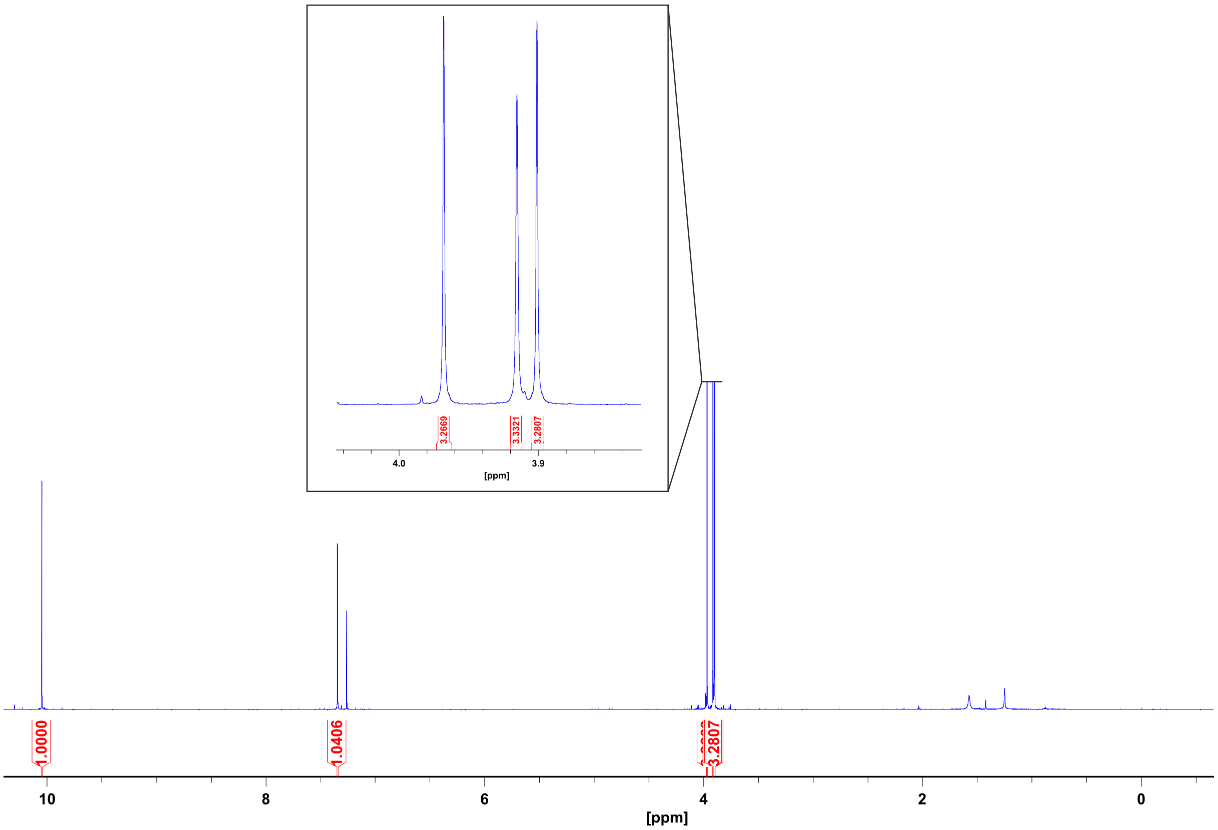

Figure S65: ^{1}H NMR spectrum of compound **20**, measured in $CDCl_3$ at 300 K.

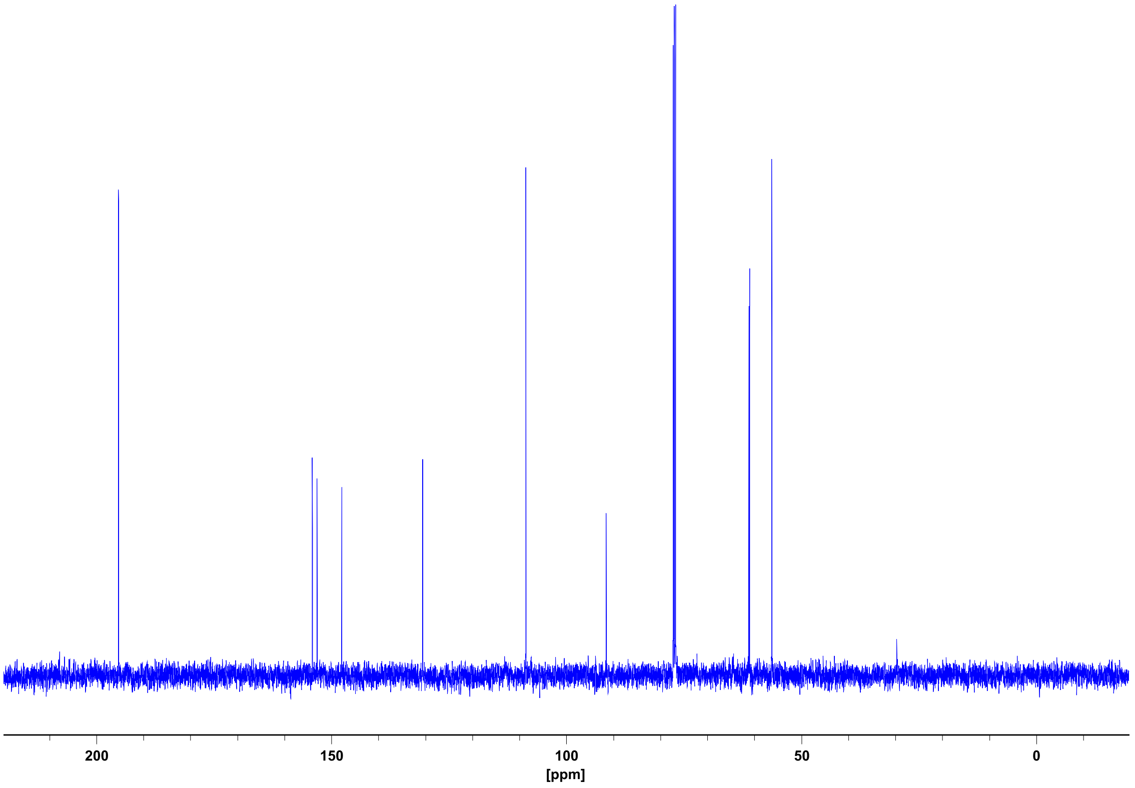

Figure S66: ^{13}C NMR spectrum of compound **20**, measured in $CDCl_3$ at 300 K.

3.7 Synthesis of 2-hydroxy-3,4,5-trimethoxybenzaldehyde (21)

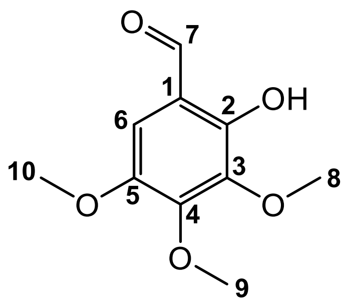

2-Iodo-3,4,5-trimethoxybenzaldehyde (**44**, 4.04 g, 12.6 mmol) was placed in a three-necked flask under a nitrogen atmosphere. Copper(I) oxide (90.0 mg, 630 µmol), pyridine-2-aldoxime (154 mg, 1.26 mmol), tetrabutylammonium bromide (811 mg, 2.52 mmol) and caesium hydroxide monohydrate (9.41 g, 63.0 mmol) were added countercurrently. Then dist. water (20 mL) was added and stirred for 18 h at room temperature. Afterwards the reaction mixture was heated to 60 °C for 4 h and DMSO (10 mL) was added for a better solubility. Then the reaction was stirred another 65 h at room temperature. A pH value of 2 was adjusted with 1 M hydrochloric acid. The organic phase was extracted with dichloromethane (4 × 60 mL) and dried over magnesium sulfate. The solvent was then removed in vacuo and analyzed by flash column chromatography (cyclohexane/ethyl acetate, gradient: 5%–60% ethyl acetate). A yellow solid was obtained.

Yield: 1.73 g (8.15 mmol, 65 %) (Lit.[1]: 83 %).

Melting Point: 44.8 °C.

^{1}H NMR (500 MHz, $CDCl_3$, 300 K): δ = 10.99 (s, 1 H, *OH*), 9.77 (s, 1 H, *H*-7), 6.78 (s, 1 H, *H*-6), 4.05 (s, 3 H, *H*-9), 3.94 (s, 3 H, *H*-8), 3.86 (s, 3 H, *H*-10) ppm.

^{13}C NMR (125 MHz, $CDCl_3$, 300 K): δ = 194.9 (*C*-7), 151.8 (*C*-5), 150.4 (*C*-3), 146.3

(*C*-4), 141.0 (*C*-1), 115.2 (*C*-2), 109.3 (*C*-6), 61.4 (*C*-9), 61.1 (*C*-8), 56.5 (*C*-10) ppm.

FT-IR: $\tilde{\nu}$ = 2947 (m), 2841 (m), 1642 (s), 1586 (w), 1488 (s), 1454 (s), 1432 (s), 1392 (s), 1319 (s), 1274 (s), 1217 (w), 1199 (m), 1140 (s), 1097 (s), 1032 (m), 987 (m), 935 (m), 897 (s), 844 (m), 781 (m), 733 (m), 610 (m) cm^{-1}.

MS (EI, 70 eV): *m/z* (%) = 212.07 (100) $[M]^{+\cdot}$.

MS (EI, HR, 70 eV): $C_{10}H_{12}O_5$ *m/z* = calc.: 212.06847, found: 212.06843, diff.: -0.22 ppm.

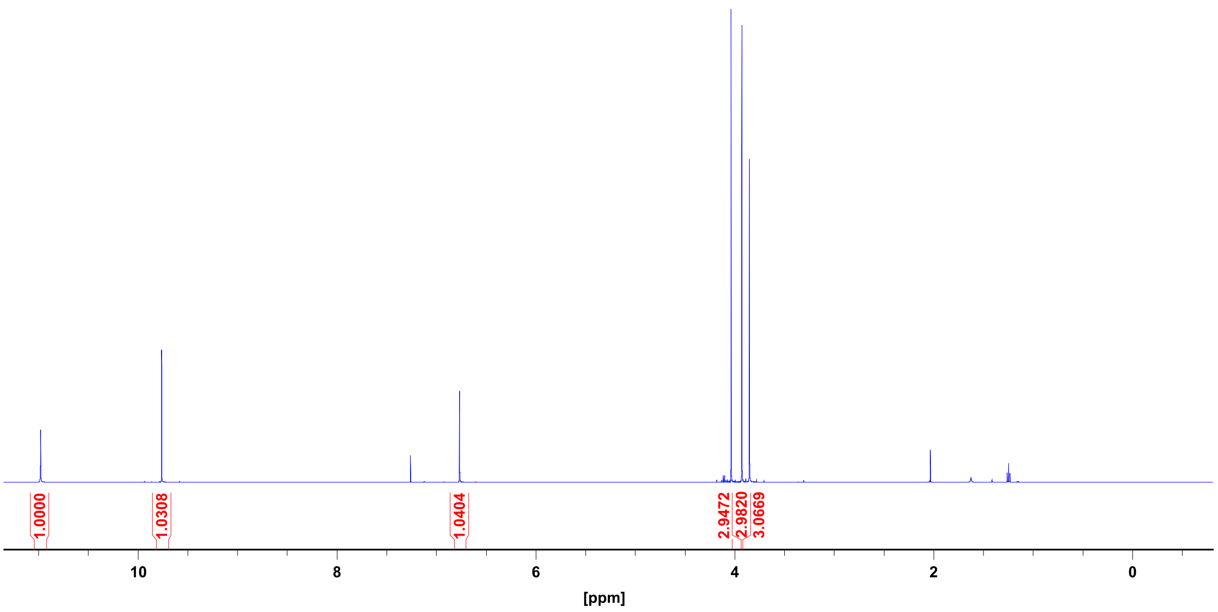

Figure S67: ^{1}H NMR spectrum of compound **21**, measured in $CDCl_3$ at 300 K.

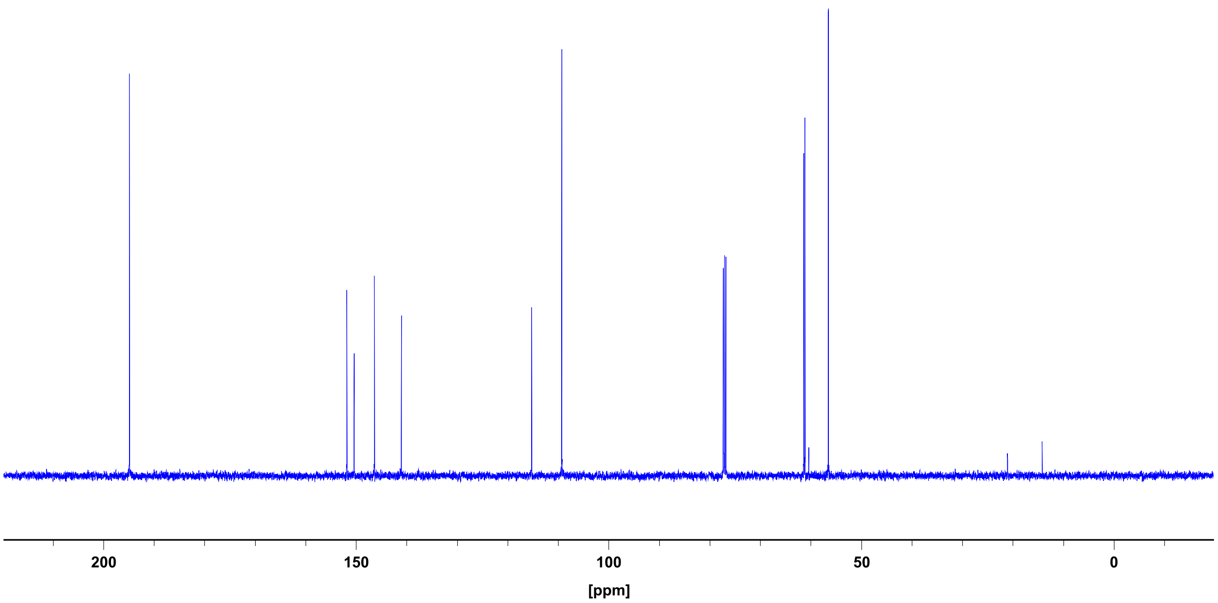

Figure S68: ^{1}H NMR spectrum of compound **21**, measured in $CDCl_3$ at 300 K.

3.8 General procedure for the ethers 22, 23 and 24

Under a nitrogen atmosphere, 2-hydroxy-3,4,5-trimethoxybenzaldehyde (**21**, 1 equiv), the corresponding fluorinated alcohol **16**–**18** (1 equiv) and cesium carbonate (1 equiv) were dissolved in *N*,*N*-dimethylacetamide (25 mL). The solution was stirred at room temperature for 3 h and then combined with dist. water (75 mL). It was extracted with ethyl acetate (3 × 50 mL) and the organic phases were separated with dist. water (2 × 50 mL) and saturated sodium chloride solution (2 × 50 mL). The organic phase was dried over magnesium sulfate, filtered and the solvent removed in vacuo. A dark yellow oil was obtained.

Methyl 2,2,3,3-tetrafluoro-4-(6-formyl-2,3,4-trimethoxyphenoxy)butanoate (22)

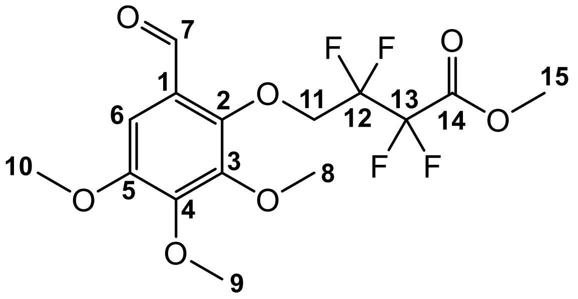

Yield: 78 %.

^{1}H NMR (500 MHz, $CDCl_3$, 300 K, TMS): δ = 10.32 (s, 1 H, *H*-7), 7.14 (s, 1 H, *H*-6), 4.64 (t, ^3J (H-F) = 13.2 Hz, 2 H, *H*-11), 4.00 (s, 3 H, *H*-9), 3.96 (s, 3 H, *H*-15), 3.92 (s, 3 H, *H*-8), 3.88 (s, 3 H, *H*-10) ppm.

^{13}C NMR (125 MHz, $CDCl_3$, 300 K, TMS): δ = 188.3 (*C*-7), 160.4 (t, ^2J (C-F) = 29.0 Hz, *C*-14), 150.4 (*C*-5), 149.5 (*C*-2), 149.3 (*C*-4), 145.8 (*C*-3), 123.7 (*C*-1), 117.0-112.9 (*C*-12-13), 104.1 (*C*-6), 69.9 (t, ^2J (C-F) = 25.6 Hz, *C*-11), 61.5 (*C*-8), 61.3 (*C*-9), 56.2 (*C*-10), 54.4 (*C*-15) ppm.

For C-12-13 the range was determined by 2D spectra (see Figure S72).

^{19}F NMR (500 MHz, $CDCl_3$, 300 K): δ = -120.5 (s, 2 F, *F*-12), -121.7 (s, 2 F, *F*-13) ppm.

FT-IR: $\tilde{\nu}$ = 2947 (w), 2250 (w), 1776 (m), 1682 (m), 1591 (m), 1486 (m), 1466 (m), 1421 (m), 1388 (m), 1342 (m), 1284 (m), 1257 (m), 1199 (m), 1127 (s), 1084 (s), 1032 (s), 986 (m), 956 (m), 922 (m), 856 (w), 818 (m), 800 (w), 750 (m), 641 (m), 596 (w) cm^{-1}.

MS (ESI): *m/z* (%) = 407.07 (100) $[M+Na]^+$.

MS (ESI, HR): $C_{15}H_{16}F_4O_7Na$ *m/z* = calc.: 407.07244, found: 407.07177, diff.: -1.64 ppm.

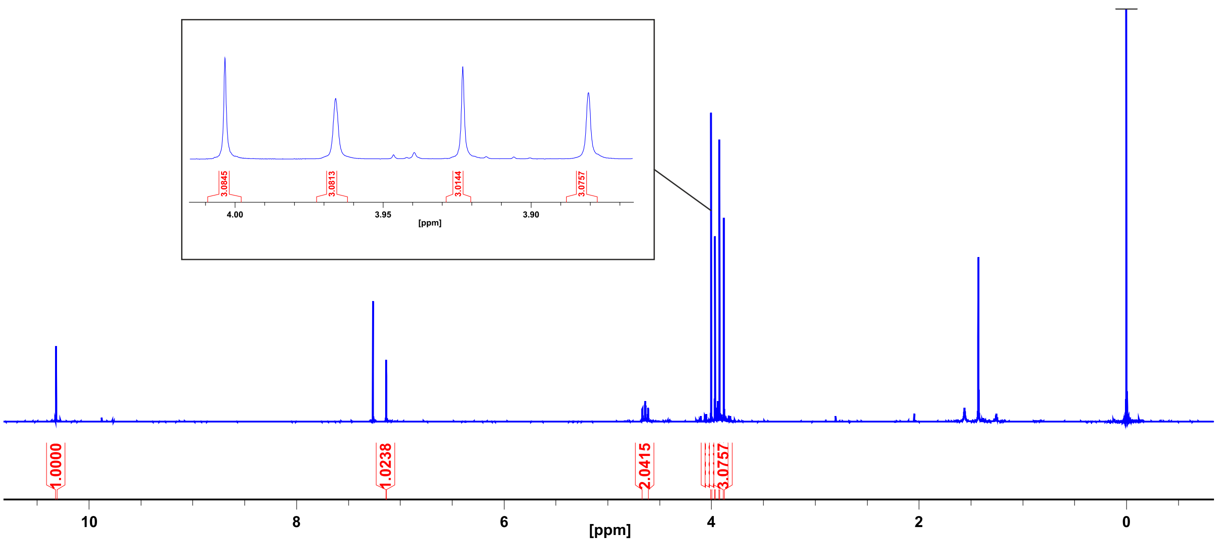

Figure S69: ^{1}H NMR spectrum of compound **22**, measured in $CDCl_3$ at 300 K.

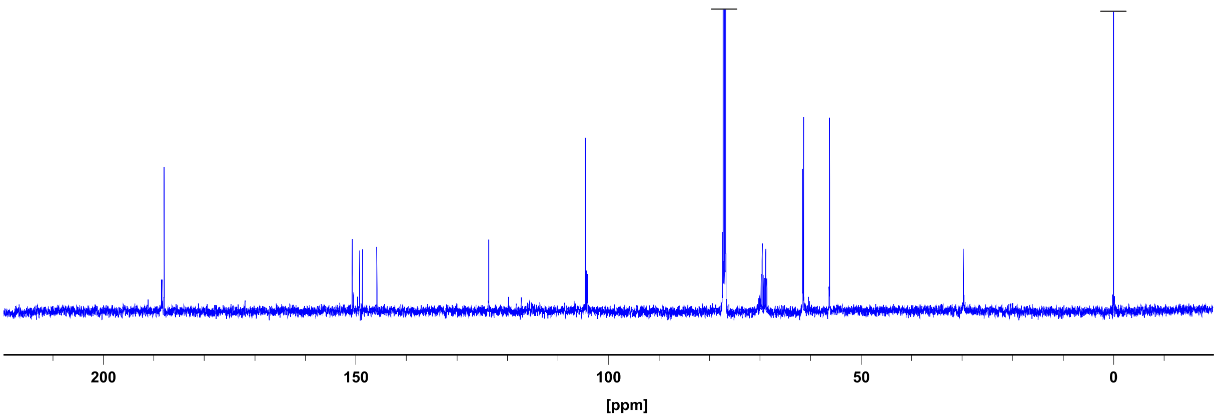

Figure S70: ^{13}C NMR spectrum of compound **22**, measured in $CDCl_3$ at 300 K.

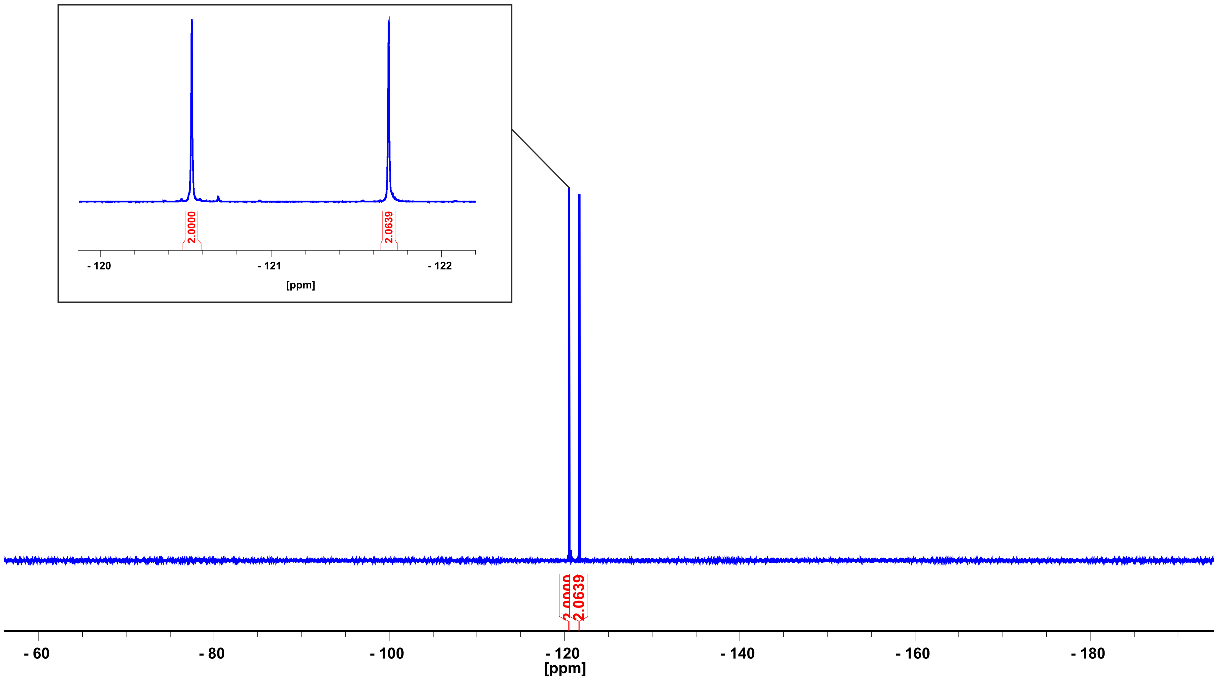

Figure S71: ^{19}F NMR spectrum of compound **22**, measured in $CDCl_3$ at 300 K.

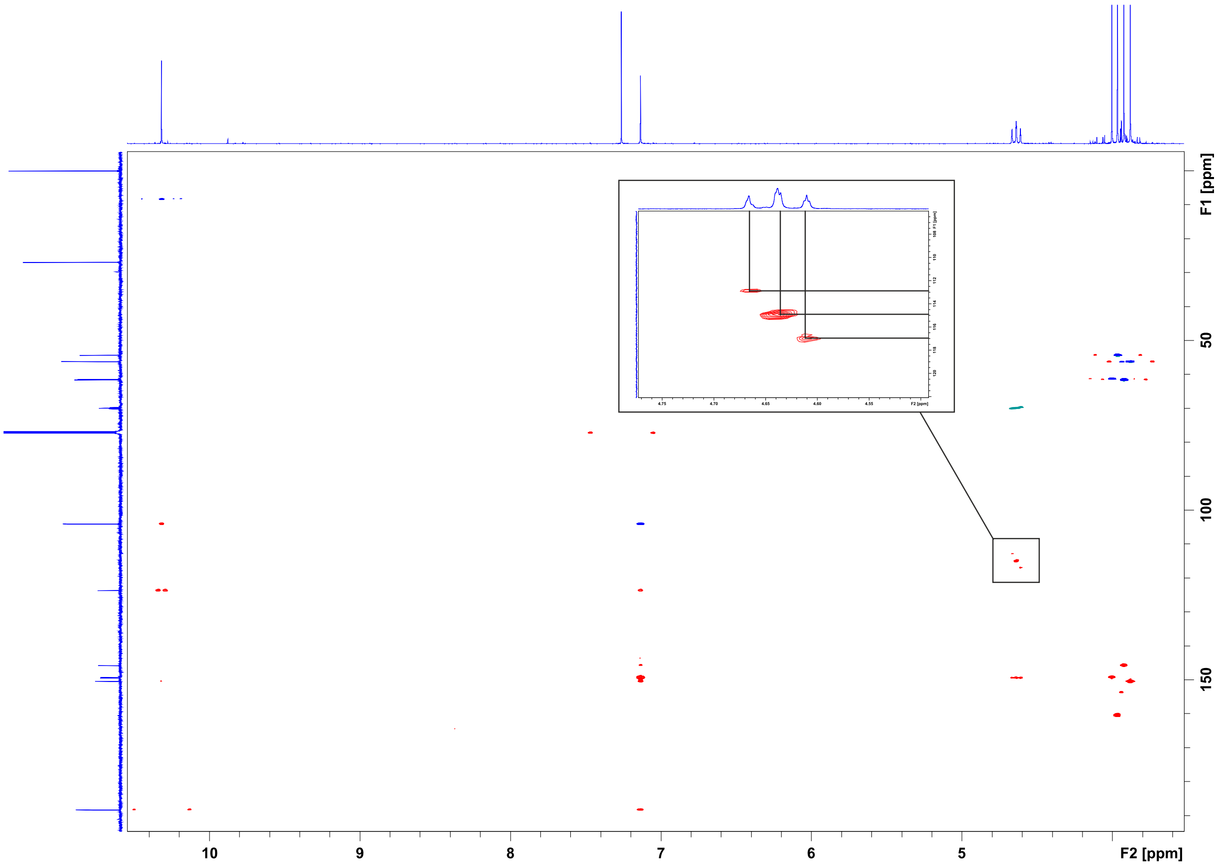

Figure S72: 2D spectra of compound **22**, showing the HSQC in blue/turquoise and the HMBC in red.

Methyl 2,2,3,3,4,4,5,5-oktafluoro-6-(6-formyl-2,3,4-trimethoxyphenoxy)-hexanoate (23)

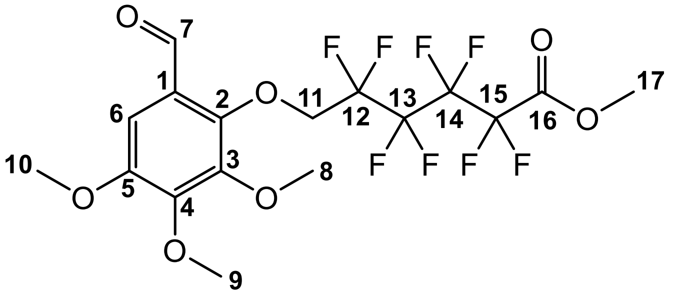

Yield: 44 %.

^{1}H NMR (500 MHz, $CDCl_3$, 300 K): δ = 10.32 (s, 1 H, *H*-7), 7.14 (s, 1 H, *H*-6), 4.63 (t, ^3J (H-F) = 14.1 Hz, 2 H, *H*-11), 4.01 (s, 3 H, *H*-9), 3.99 (s, 3 H, *H*-17), 3.92 (s, 3 H, *H*-8), 3.88 (s, 3 H, *H*-10) ppm.

^{13}C NMR (125 MHz, $CDCl_3$, 300 K): δ = 188.1 (*C*-7), 159.2 (*C*-16), 150.4 (*C*-5), 149.3 (*C*-2), 149.2 (*C*-4), 145.5 (*C*-3), 123.5 (*C*-1), 117.2-113.0 (*C*-12-15), 104.1 (*C*-6), 69.9 (t, ^2J (C-F) = 24.2 Hz, *C*-11), 61.5 (*C*-8), 61.3 (*C*-9), 56.2 (*C*-10), 54.6 (*C*-17) ppm.

Due to the large number of ^{19}F-coupled C atoms, it was not possible to obtain sufficient signal intensity for the *C*-16 atom. The shift of C-16 was determined by 2D spectra. For C-12-15 the range was determined also by 2D spectra (see Figure S76).

^{19}F NMR (500 MHz, $CDCl_3$, 300 K): δ = -119.1 (t, ^3J (H-F) = 11.7 Hz, 2 F, *F*-15), -120.9 (dt, ^3J (H-F) = 14.3 Hz, ^4J (H-F) = 2.80 Hz, 2 F, *F*-12), -123.4 to -123.5 (m, 2 F, *F*-13), -123.7 to -123.9 (m, 2 F, *F*-14) ppm.

FT-IR: $\tilde{\nu}$ = 2946 (m), 1782 (m), 1684 (m), 1591 (m), 1486 (m), 1467 (m), 1421 (m), 1389 (m), 1343 (s), 1283 (m), 1187 (s), 1133 (s), 1086 (s), 1046 (m), 987 (w), 960 (w), 925 (w), 868 (m), 809 (m), 757 (m), 711 (w), 637 (m) cm^{-1}.

MS (EI, 70 eV): *m/z* (%) = 484 (90) $[M]^{+\cdot}$, 211.06 (100) $[M-C_7H_5F_8O_2]^{+\cdot}$.

MS (EI, HR, 70 eV): $C_{17}H_{16}F_8O_7$ *m/z* = calc.: 484.07683, found: 484.07686, diff.: 0.06 ppm.

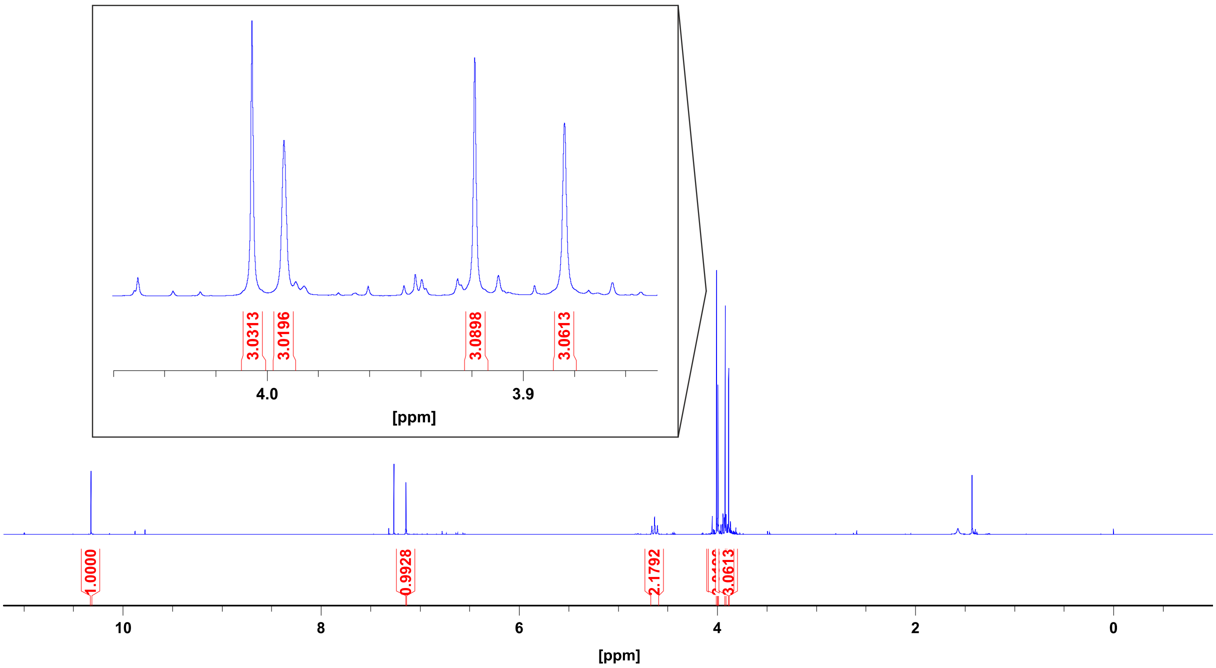

Figure S73: ^{1}H NMR spectrum of compound **23**, measured in $CDCl_3$ at 300 K.

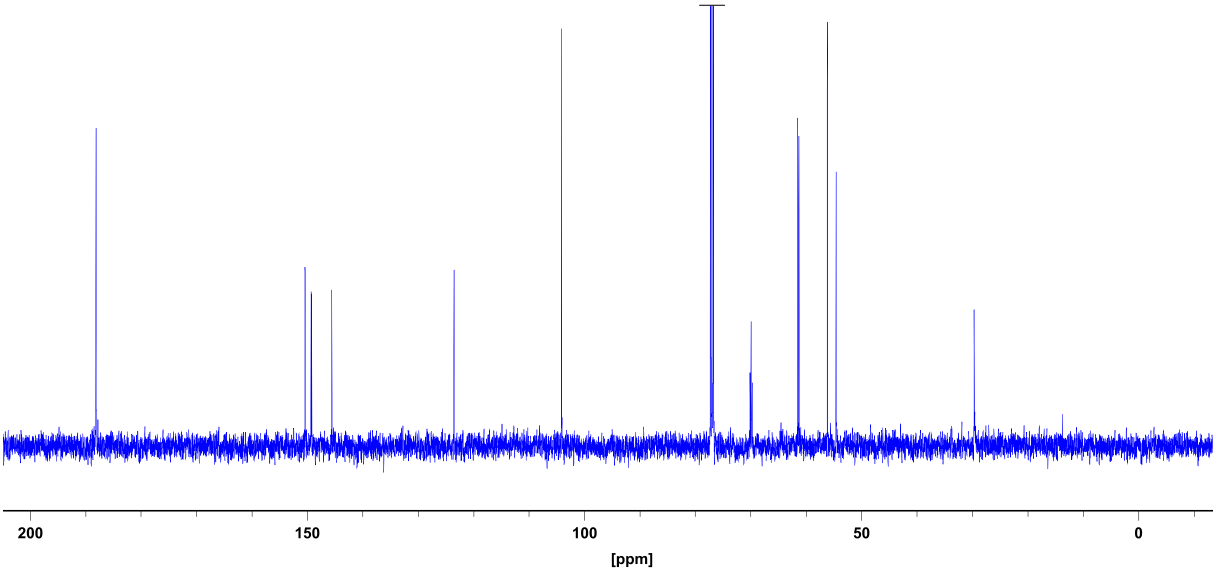

Figure S74: ^{13}C NMR spectrum of compound **23**, measured in $CDCl_3$ at 300 K.

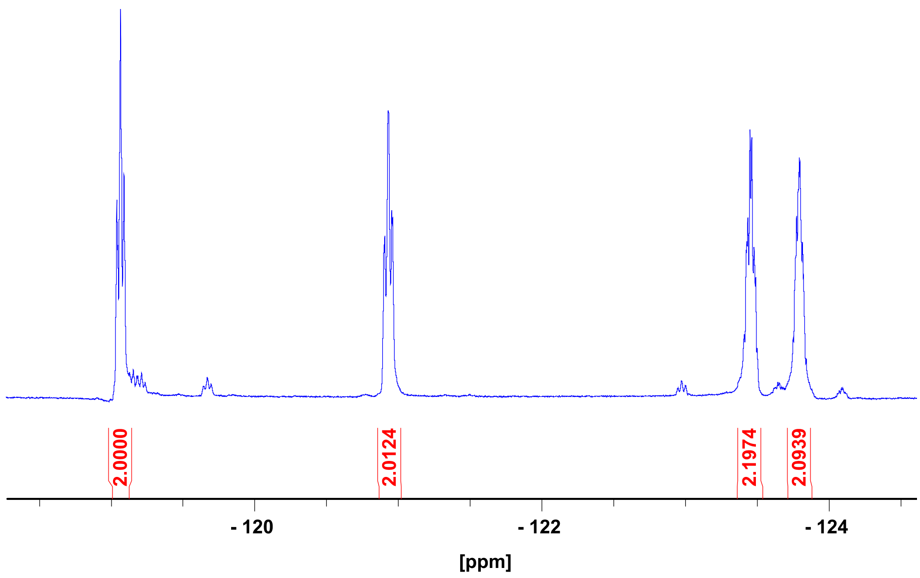

Figure S75: ^{19}F NMR spectrum of compound **22**, measured in $CDCl_3$ at 300 K.

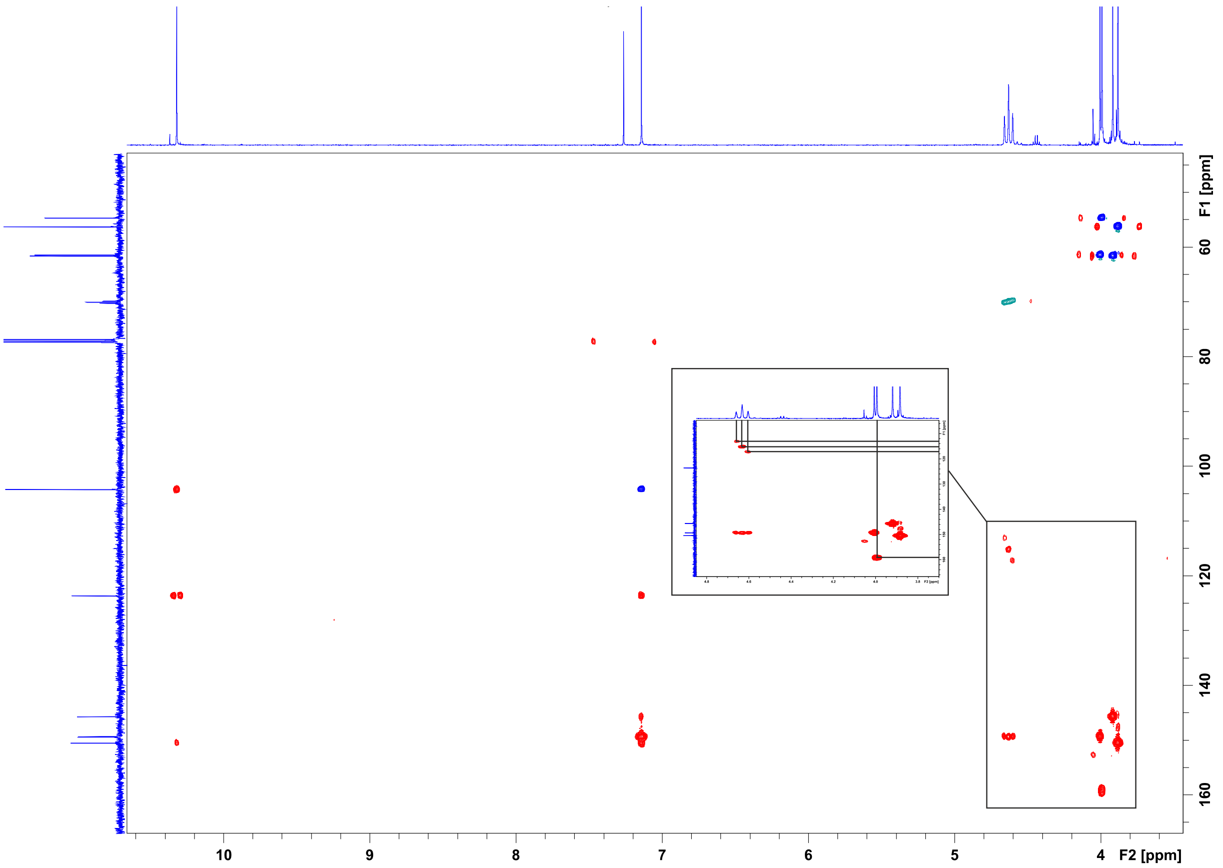

Figure S76: 2D spectra of compound **23**, showing the HSQC in blue/turquoise and the HMBC in red.

Methyl 2,2,3,3,4,4,5,5,6,6,7,7-dodecafluoro-8-(6-formyl-2,3,4-trimethoxyphen-oxy)octanoate (24)

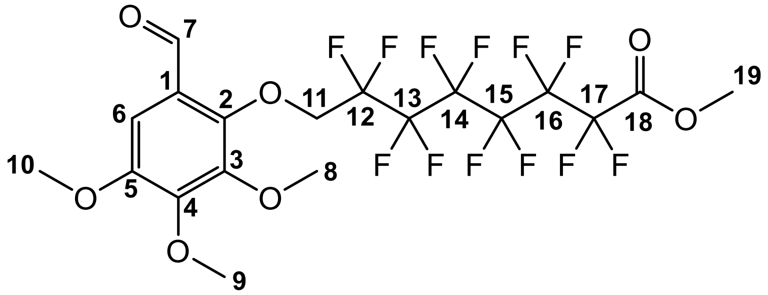

Yield: 44 %.

^{1}H NMR (500 MHz, $CDCl_3$, 300 K, TMS): δ = 10.32 (s, 1 H, *H*-7), 7.15 (s, 1 H, *H*-6), 4.64 (t, ^3J (H-F) = 14.0 Hz, 2 H, *H*-11), 4.01 (s, 3 H, *H*-9), 4.00 (s, 3 H, *H*-17), 3.92 (s, 3 H, *H*-8), 3.89 (s, 3 H, *H*-10) ppm.

^{13}C NMR (125 MHz, $CDCl_3$, 300 K): δ = 188.0 (*C*-7), 159.0 (t, ^2J (C-F) = 27.4, *C*-18), 150.5 (*C*-5), 149.3 (*C*-2), 149.2 (*C*-4), 145.6 (*C*-3), 123.4 (*C*-1), 117.3-113.1 (*C*-12-14), 104.2 (*C*-6), 69.9 (t, ^2J (C-F) = 24.0, *C*-11), 61.5 (*C*-8), 61.3 (*C*-9), 56.2 (*C*-10), 54.7 (*C*-19) ppm.

Due to the large number of ^{19}F-coupled C atoms, it was not possible to obtain sufficient signal intensity for the *C*-15, *C*-16 and *C*-17 atom. For C-12-14 the range was determined also by 2D spectra (see Figure 80).

^{19}F NMR (500 MHz, $CDCl_3$, 300 K): δ = -118.9 to -118.0 (m, 2 F, *F*-17), -120.8 to -120.9 (m, 2 F, *F*-12), -122.1 to -122.3 (m, 2 F, *F*-15), -122.3 to -122.5 (m, 2 F, *F*-14), -123.2 to -123.3 (m, 2 F, *F*-13), -123.6 to -123.8 (m, 2 F, *F*-16) ppm.

FT-IR: $\tilde{\nu}$ = 2946 (m), 1784 (m), 1685 (m), 1591 (m), 1487 (m), 1467 (m), 1432 (w), 1421 (m), 1389 (m), 1344 (m), 1283 (w), 1198 (s), 1133 (s), 1087 (s), 1036 (m), 986 (w), 962 (w), 925 (w), 842 (w), 805 (w), 755 (m), 716 (m), 640 (m) cm^{-1}.

MS (EI, 70 eV): *m/z* (%) = 584.07 (82) $[M]^{+\cdot}$, 211.06 (100) $[M-C_9H_5F_{12}O_2]^{+\cdot}$.

MS (EI, HR, 70 eV): $C_{19}H_{16}F_{12}O_7$ *m/z* = calc.: 584.07044, found: 584.07020, diff.: -0.42 ppm.

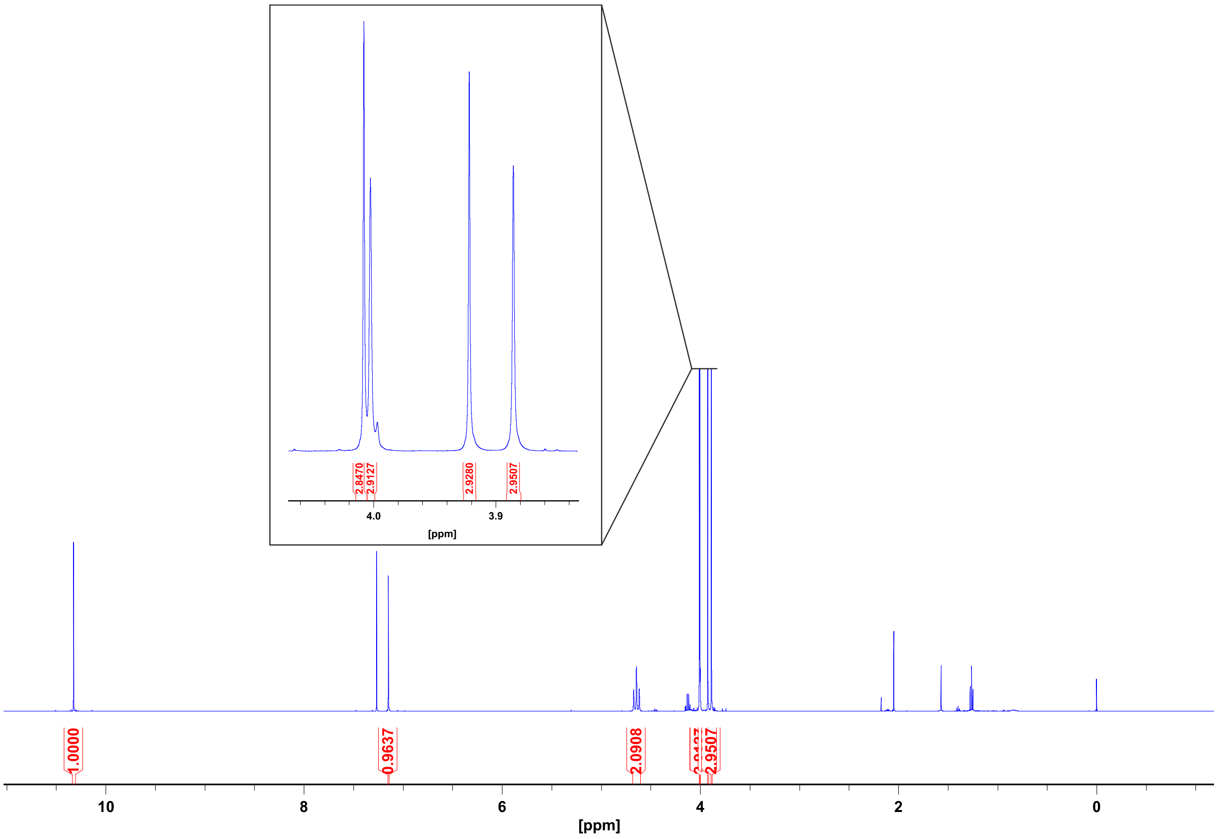

Figure S77: ^{1}H NMR spectrum of compound **24**, measured in $CDCl_3$ at 300 K.

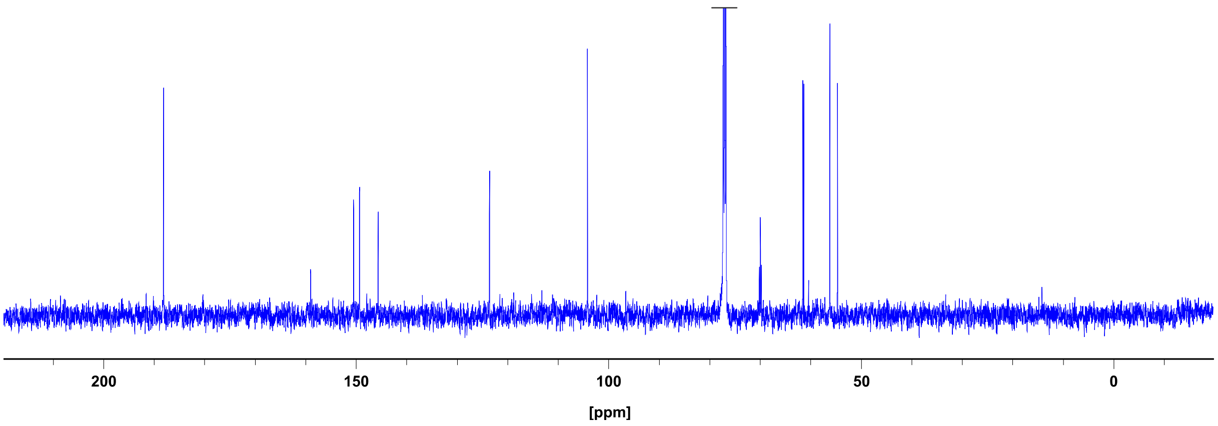

Figure S78: ^{13}C NMR spectrum of compound **24**, measured in $CDCl_3$ at 300 K.

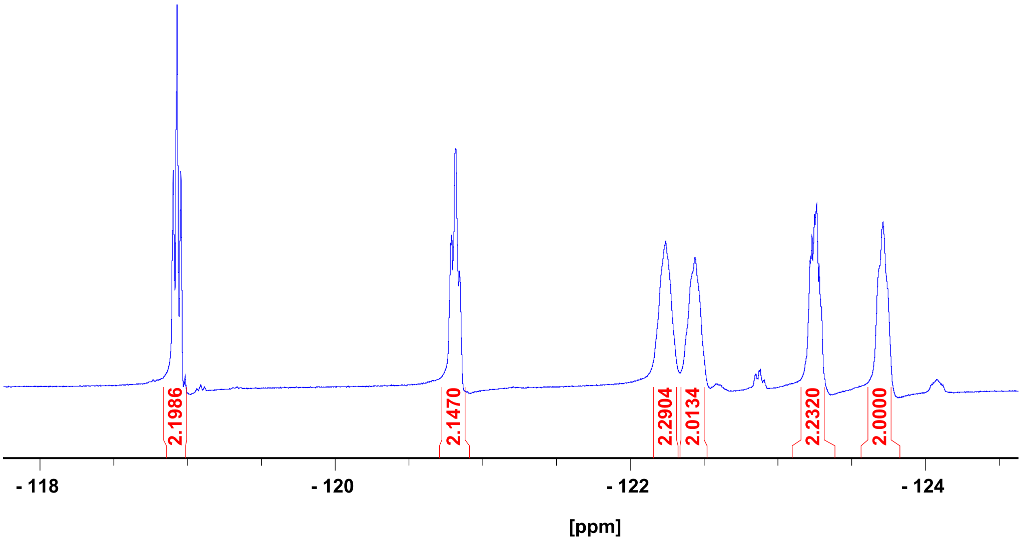

Figure S79: ^{19}F NMR spectrum of compound **24**, measured in $CDCl_3$ at 300 K.

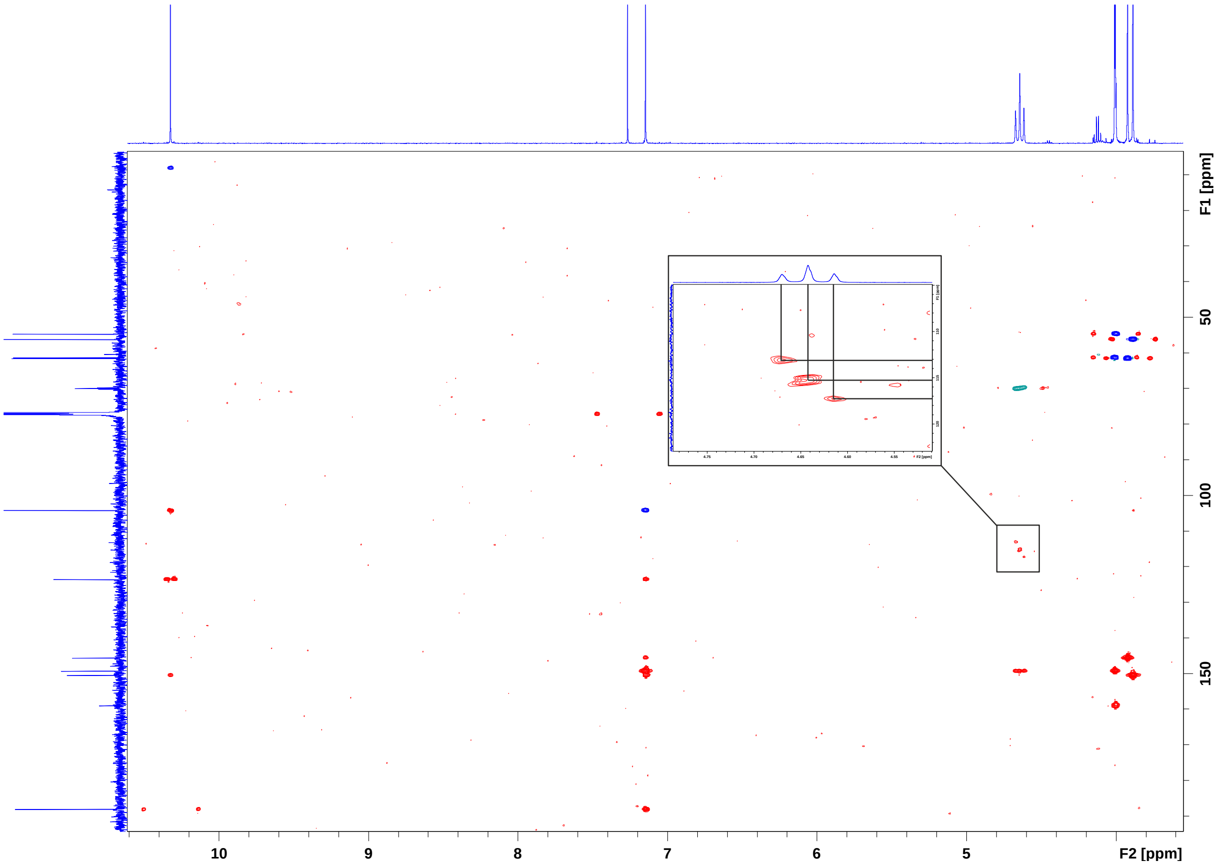

Figure S80: 2D spectra of compound **24**, showing the HSQC in blue/turquoise and the HMBC in red.

3.9 General procedure for the metal-free porphyrins 26, 27 and 28

Under a nitrogen atmosphere, the corresponding aldehyde **22–24** (1 equiv) and trifluoroacetic acid (0.31 equiv) were introduced into abs. dichloromethane (50 mL) and the solution was refluxed for 30 min. Pyrrole (1 equiv) was added and the reaction mixture was stirred under reflux for a further 2.5 h. After the addition of 2,3-dichloro-5,6-dicyano-1,4-benzoquinone (DDQ, 0.48 equiv) it was again refluxed for 2 h. The crude product was purified on silica gel by column chromatography (cyclohexane/ethyl acetate 1:1). Afterwards the product was dissolved in dichloromethane (50 mL), washed with saturated sodium carbonate solution (3 × 50 mL), dried over magnesium sulfate and the solvent was removed in vacuo. A purple, crystalline solid was obtained.

5,10,15,20-Tetrakis-(2-(2,2,3,3-tetrafluoro-3-methoxycarbonylprop-1-yloxy)-3,4,5-trimethoxyphenyl)-21*H*,23*H*-porphyrin (26)

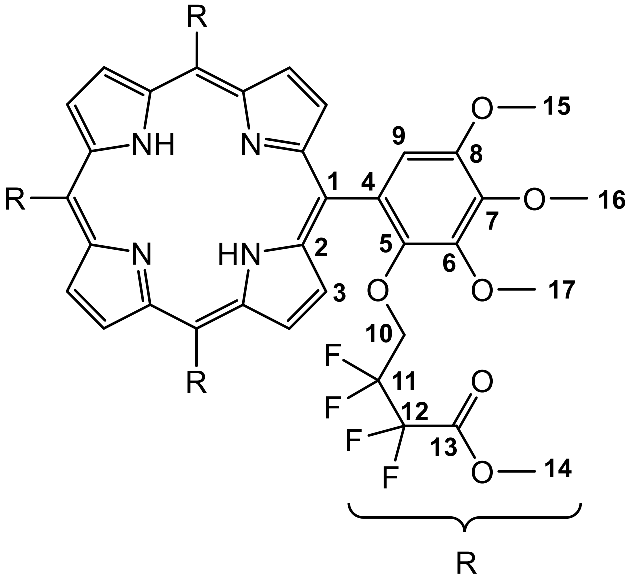

Yield: 9 %.

Melting Point: 61.8 °C.

^{1}H NMR (500 MHz, $CDCl_3$, 300 K): δ = 8.56-8.39 (m, 8 H, *H*-3), 7.65-7.32 (m, 4 H, *H*-9), 4.29-3.71 (m, 44 H, *H*-10, *H*-15, *H*-16, *H*-17), 3.35-2.03 (m, 12 H, *H*-14), -2.71 to -2.76 (m, 2H, N-*H*) ppm.

Due to atropisomers, an exact evaluation of the ^{1}H NMR spectrum was not possible and only the ranges of the signals were given. A qualitative evaluation of the ^{13}C NMR and ^{19}F NMR spectrum was not possible.

FT-IR: $\tilde{\nu}$ = 2923 (m), 2249 (w), 1774 (m), 1583 (m), 1492 (m), 1457 (m), 1413 (m), 1367 (m), 1228 (m), 1120 (s), 1034 (s), 954 (m), 804 (m), 750 (m), 594 (m) cm^{-1}.

MS (ESI): *m/z* (%) = 1727.42 (100) $[M+H]^+$.

MS (ESI, HR): $C_{76}H_{70}F_{16}N_4O_{24}$ *m/z* = calc.: 1727.41973, found: 1727.42122, diff.: 0.87 ppm.

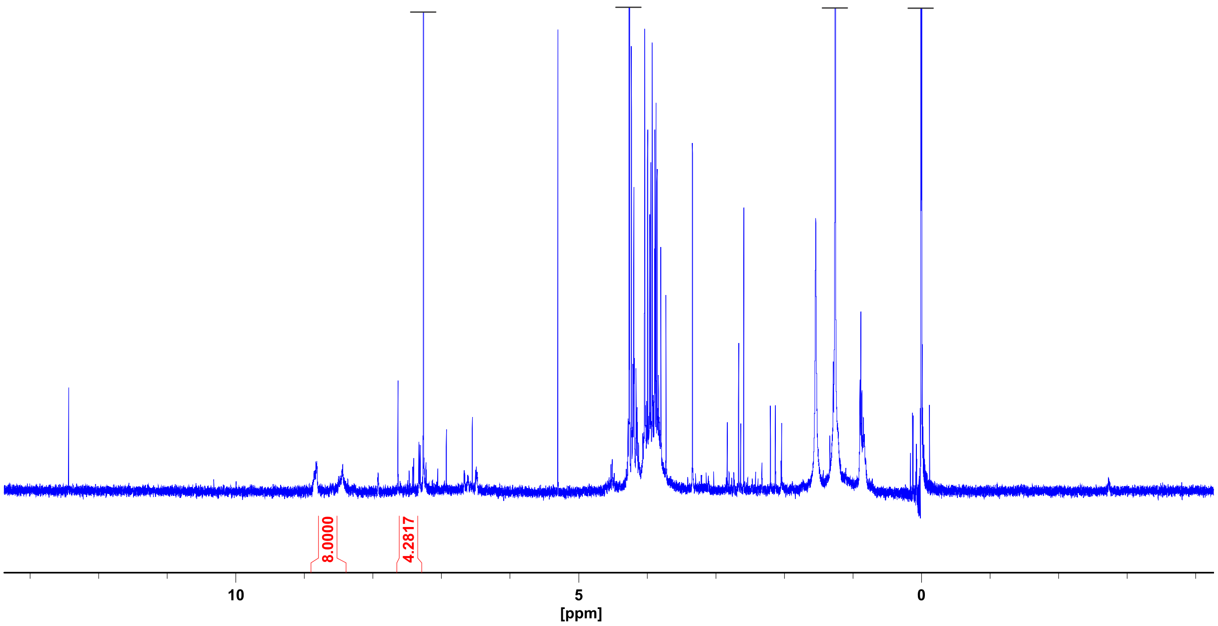

Figure S81: ^{1}H NMR spectrum of compound **26**, measured in $CDCl_3$ at 300 K.

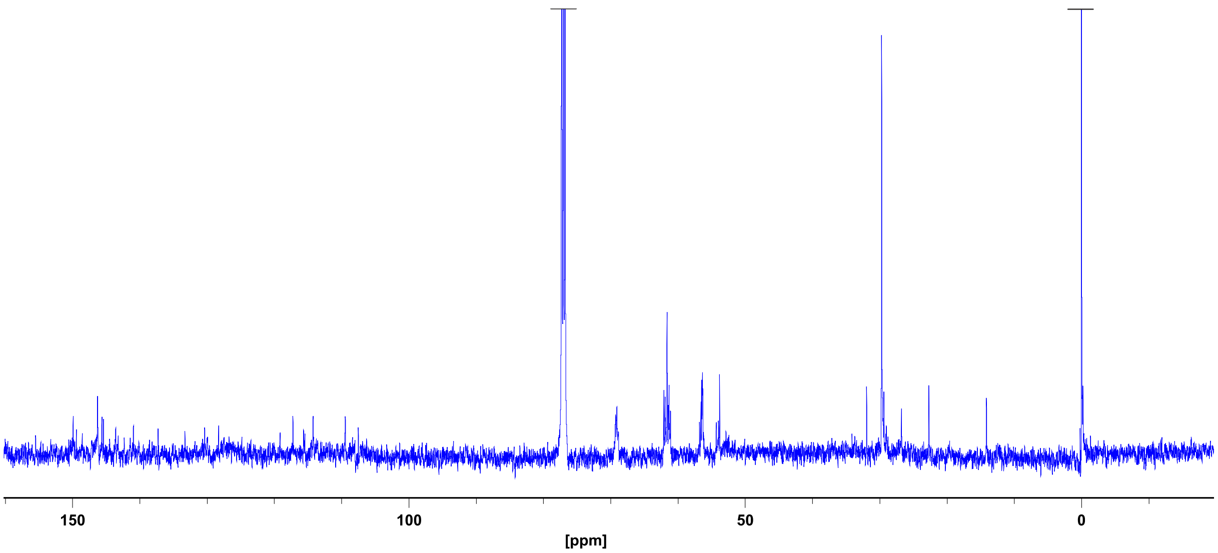

Figure S82: ^{13}C NMR spectrum of compound **26**, measured in $CDCl_3$ at 300 K.

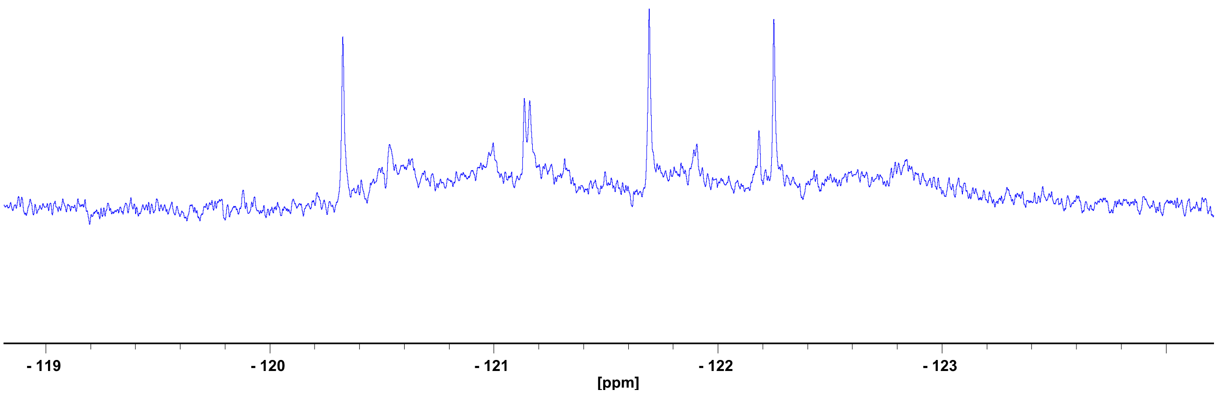

Figure S83: ^{19}F NMR spectrum of compound **26**, measured in $CDCl_3$ at 300 K.

5,10,15,20-Tetrakis-(2-(2,2,3,3,4,4,5,5-octafluoro-5-methoxycarbonylpent-1-yloxy)-3,4,5-trimethoxyphenyl)-21*H*,23*H*-porphyrin (27)

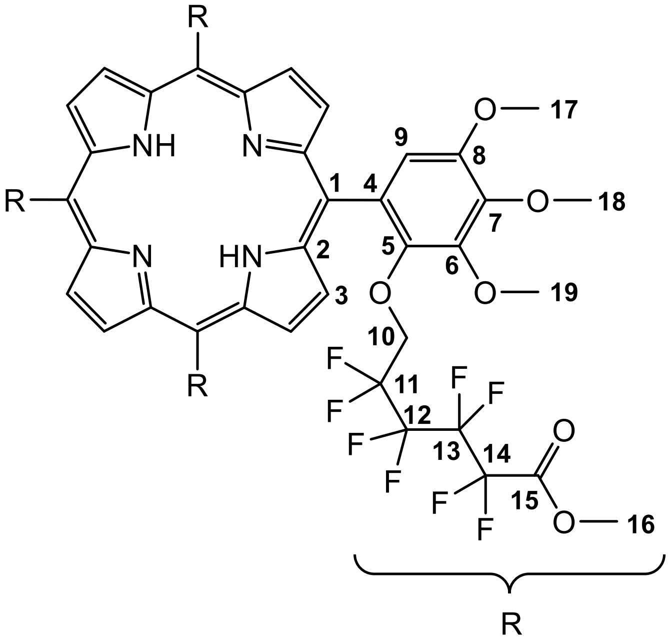

Yield: 18 %.

Melting Point: 181-185 °C.

^{1}H NMR (500 MHz, $CDCl_3$, 300 K): δ = 8.87-8.79 (m, 8 H, *H*-3), 7.43-7.26 (m, 4 H, *H*-9), 4.25-2.42 (m, 44 H, *H*-10, *H*-17, *H*-18, *H*-19), 3.45-2.04 (m, 12 H, *H*-16), -2.69 to -2.84 (m, 2 H, N-*H*) ppm.

Due to atropisomers, an exact evaluation of the ^{1}H NMR spectrum was not possible and only the ranges of the signals were given. A qualitative evaluation of the ^{13}C NMR and ^{19}F NMR spectrum was not possible.

FT-IR: $\tilde{\nu}$ = 3184 (w), 2943 (w), 2255 (w), 1780 (m), 1575 (w), 1492 (w), 1452 (m), 1413 (w), 1357 (w), 1324 (w), 1268 (w), 1183 (m), 1137 (m), 1103 (m), 1073 (m), 1037 (m), 1007 (w), 957 (w), 922 (w), 886 (w), 867 (w), 803 (w), 775 (w), 744 (w), 688 (w), 618 (w) cm^{-1}.

MS (ESI): *m/z* (%) = 2127.39 (100) [M-H]$^+$.

MS (ESI, HR): $C_{84}H_{71}O_{24}N_4F_{32}$: calc.: 2127.39418, found: 2127.39382, diff.: -0.17 ppm.

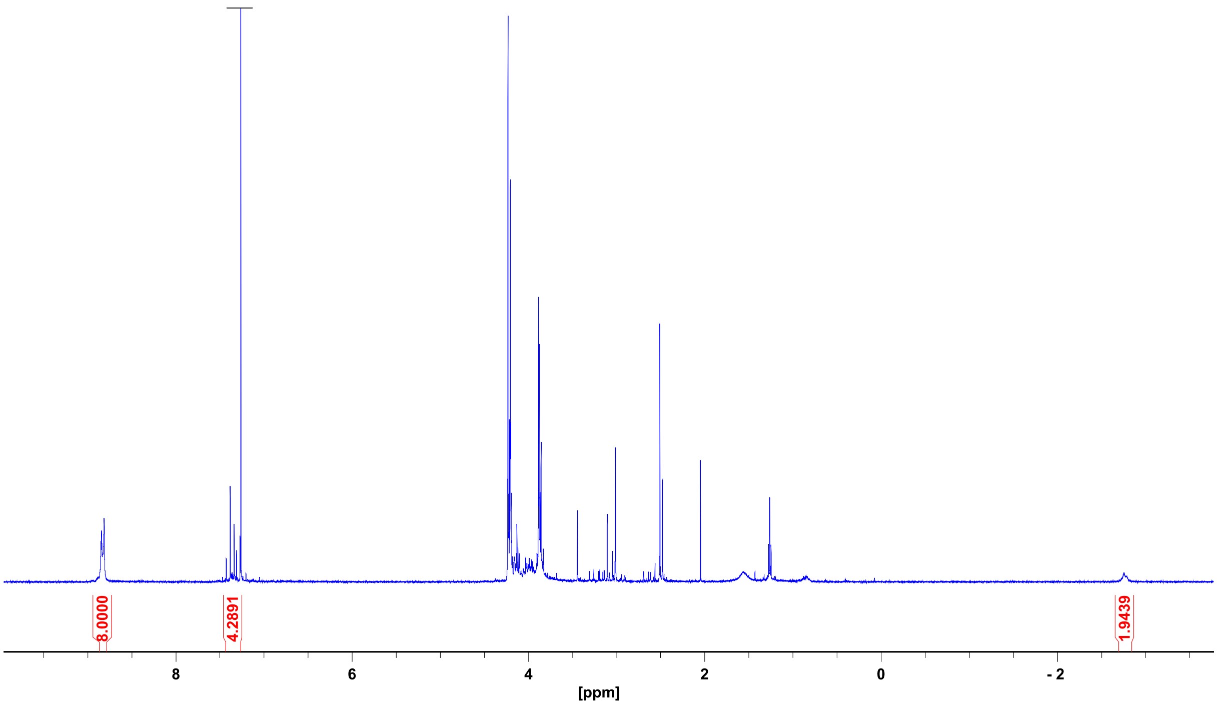

Figure S84: ^{1}H NMR spectrum of compound **27**, measured in $CDCl_3$ at 300 K.

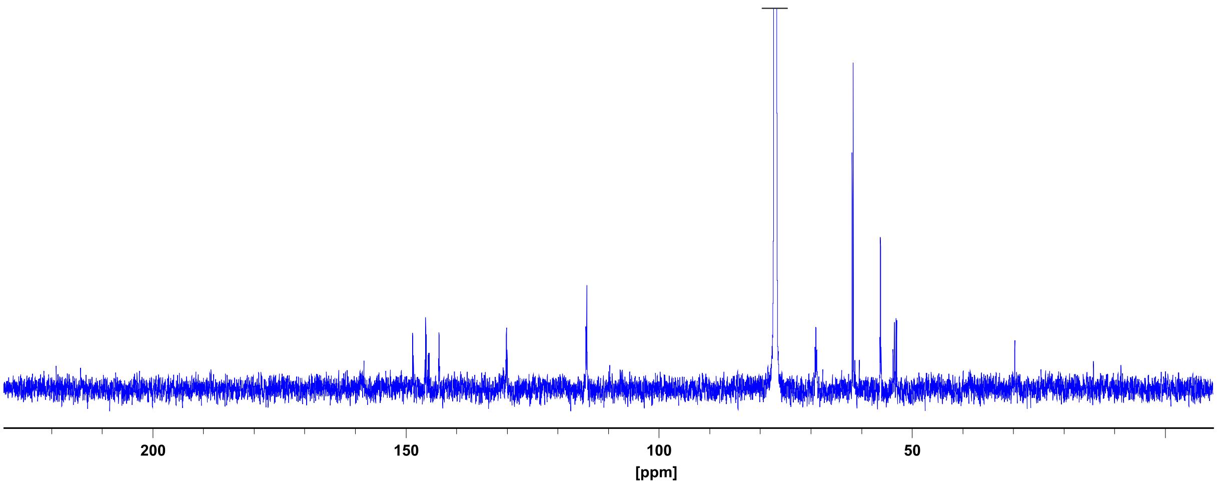

Figure S85: ^{13}C NMR spectrum of compound **27**, measured in $CDCl_3$ at 300 K.

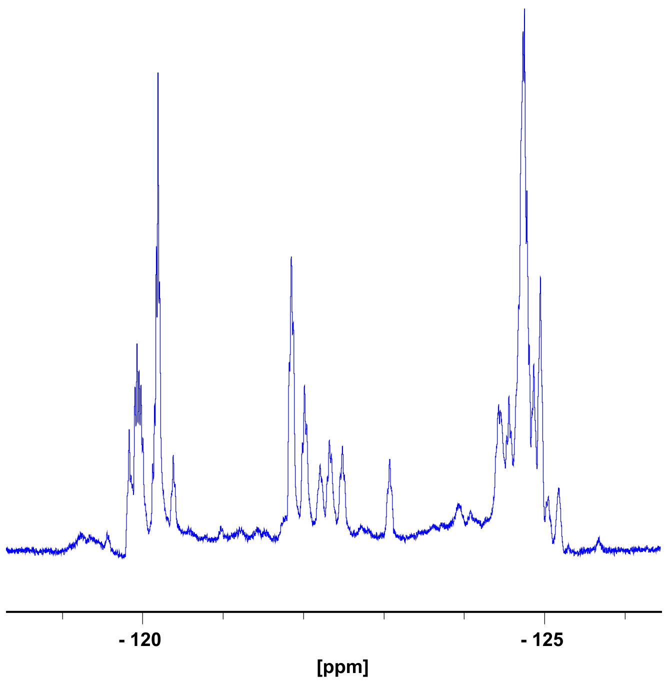

Figure S86: ^{19}F NMR spectrum of compound **27**, measured in $CDCl_3$ at 300 K.

5,10,15,20-Tetrakis-(2-(2,2,3,3,4,4,5,5,6,6,7,7-dodecafluoro-7-methoxycarbonyl-hept-1-yloxy)-3,4,5-trimethoxyphenyl)-21*H*,23*H*-porphyrin (28)

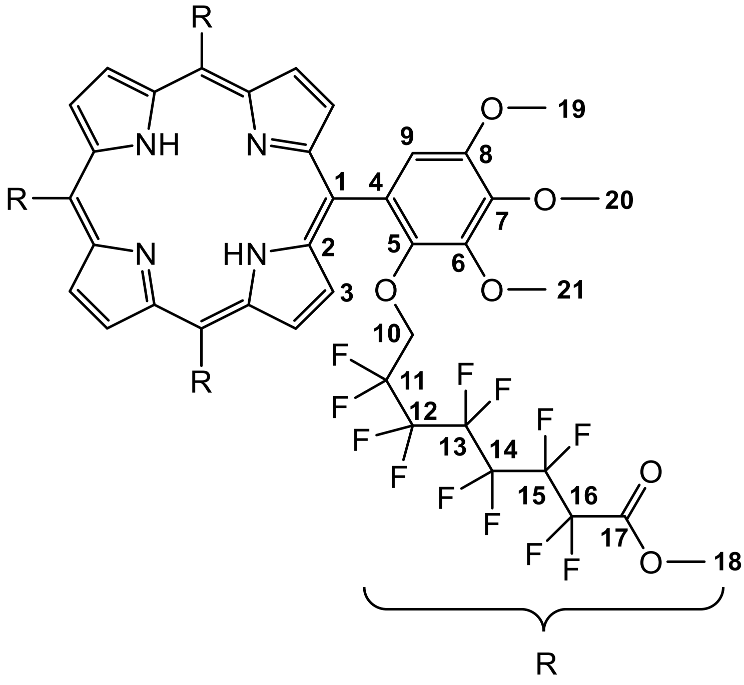

Yield: 21 %.

Melting Point: 72.5 °C.

^{1}H NMR (500 MHz, $CDCl_3$, 300 K, TMS): δ = 8.89-8.80 (m, 8 H, *H*-3), 7.46-7.21 (m, 4 H, *H*-9), 4.25-2.09 (m, 56 H, *H*-10, *H*-18, *H*-19, *H*-20, *H*-21), -2.68 to -2.75 (m, 2 H, N-*H*) ppm.

Due to atropisomers, an exact evaluation of the ^{1}H NMR spectrum was not possible and only the ranges of the signals were given. A qualitative evaluation of the ^{13}C NMR and ^{19}F NMR spectrum was not possible.

FT-IR: $\tilde{\nu}$ = 2917 (w), 2849 (w), 1782 (w), 1690 (w), 1577 (w),1491 (w),1459 (w), 1434 (w), 1413 (w), 1356 (w), 1324 (w), 1195 (m), 1137 (m), 1103 (m), 1038 (w), 1011 (w), 956 (w), 923 (w), 899 (w), 803 (w), 744 (w), 717 (w), 653 (w), 624 (w) cm^{-1}.

MS (ESI): *m/z* (%) = 2527.37 (100) $[M]^{+}$.

MS (ESI, HR): $C_{92}H_{71}F_{48}N_4O_{24}$ *m/z* = calc.: 2527.36863, found: 2527.36760, diff.: -0.41 ppm.

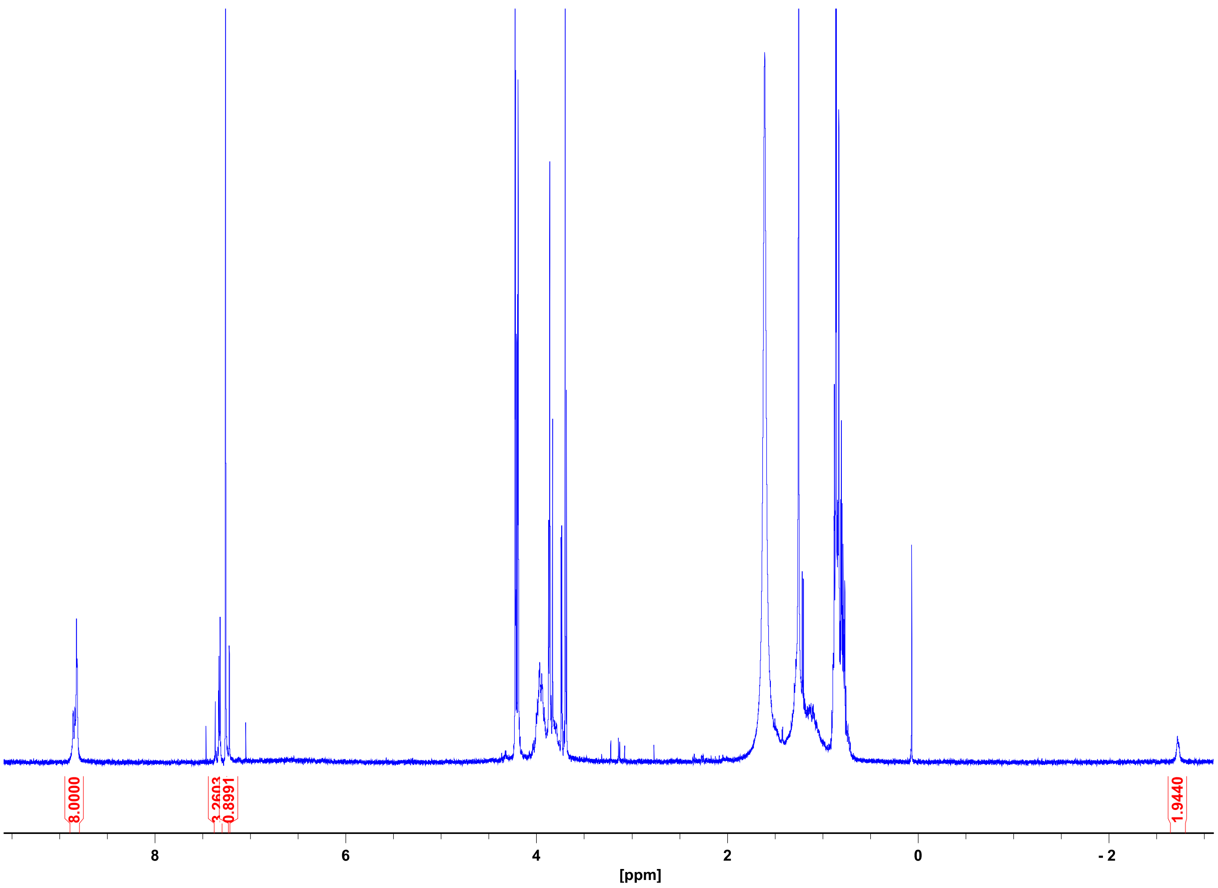

Figure S87: ^{1}H NMR spectrum of compound **28**, measured in $CDCl_3$ at 300 K.

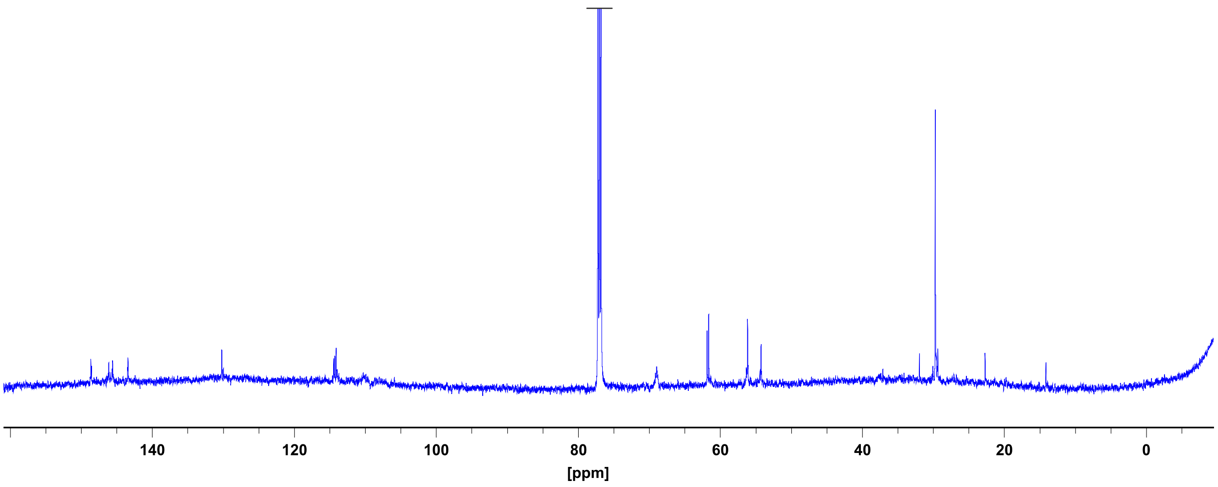

Figure S88: ^{13}C NMR spectrum of compound **28**, measured in $CDCl_3$ at 300 K.

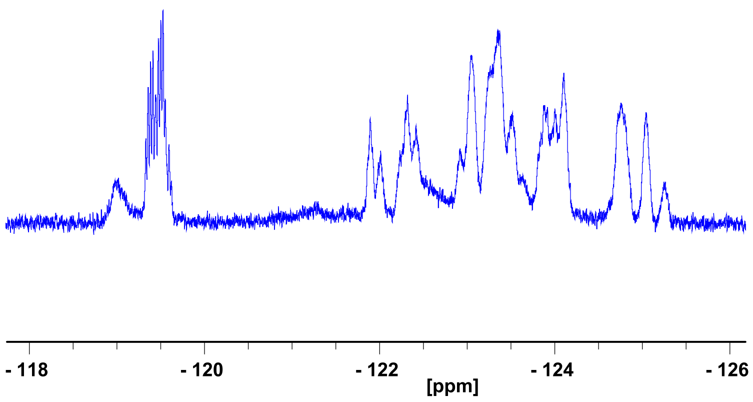

Figure S89: ^{19}F NMR spectrum of compound **28**, measured in $CDCl_3$ at 300 K.

3.10 General procedure for the nickel porphyrins 29, 30 and 31

The corresponding porphyrin **26–28** (1 equiv) was dissolved in toluene (25 mL), nickel(II) acetylacetonate (10 equiv) was added and refluxed for 20 h. The solvent was removed in vacuo and the crude product was purified by column chromatography on silica gel (cyclohexane/ethyl acetate 1:1) by dry loading. A brown, crystalline solid was obtained.

5,10,15,20-Tetrakis-(2-(2,2,3,3-tetrafluoro-3-methoxycarbonylprop-1-yloxy)-3,4,5-trimethoxyphenyl)-nickel(II)porphyrin (29)

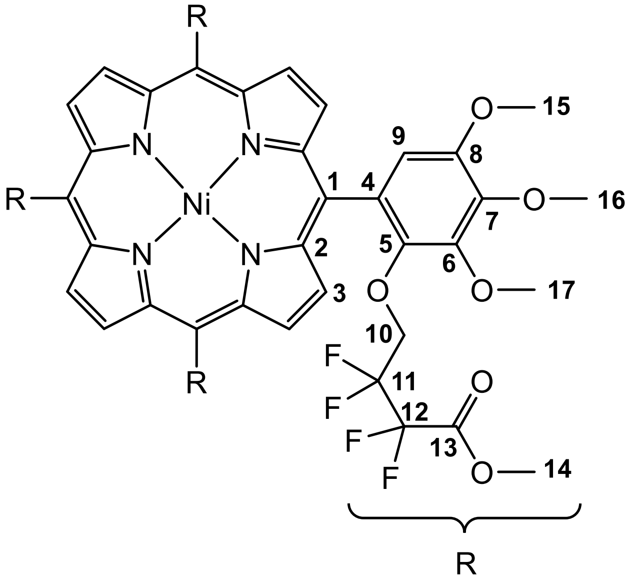

Yield: 78 %.

Melting Point: 84.9 °C.

^{1}H NMR (500 MHz, $CDCl_3$, 300 K, TMS): δ = 8.78-8.70 (m, 8 H, *H*-3), 7.35-6.95 (m, 4 H, *H*-9), 4.15-3.77 (m, 44 H, *H*-10,15,16,17), 3.05-2.52 (m, 12 H, *H*-14) ppm.

Due to atropisomers, an exact evaluation of the ^{1}H NMR and ^{19}F NMR spectrum was not possible and only the ranges of the signals were given. A qualitative evaluation of the ^{13}C NMR spectrum was not possible.

^{19}F NMR (500 MHz, $CDCl_3$, 300 K): δ = -121.3 to -122.3 (m, 2 F, *F*-11), -122.5 to -123.5 (m, 2 F, *F*-12) ppm.

FT-IR: $\tilde{\nu}$ = 2937 (w), 2252 (w), 1982 (w), 1774 (m), 1490 (m), 1456 (m), 1412 (m), 1366 (s), 1110 (s), 1075 (s), 1036 (s), 1006 (s), 751 (s), 632 (m) cm^{-1}.

MS (ESI): *m/z* (%) = 1782.33 (100) $[M]^+$.

MS (ESI, HR): $C_{76}H_{68}F_{16}N_4O_{24}Ni$ *m/z* = calc.: 1782.33160, found: 1782.33356, diff.: 1.10 ppm.

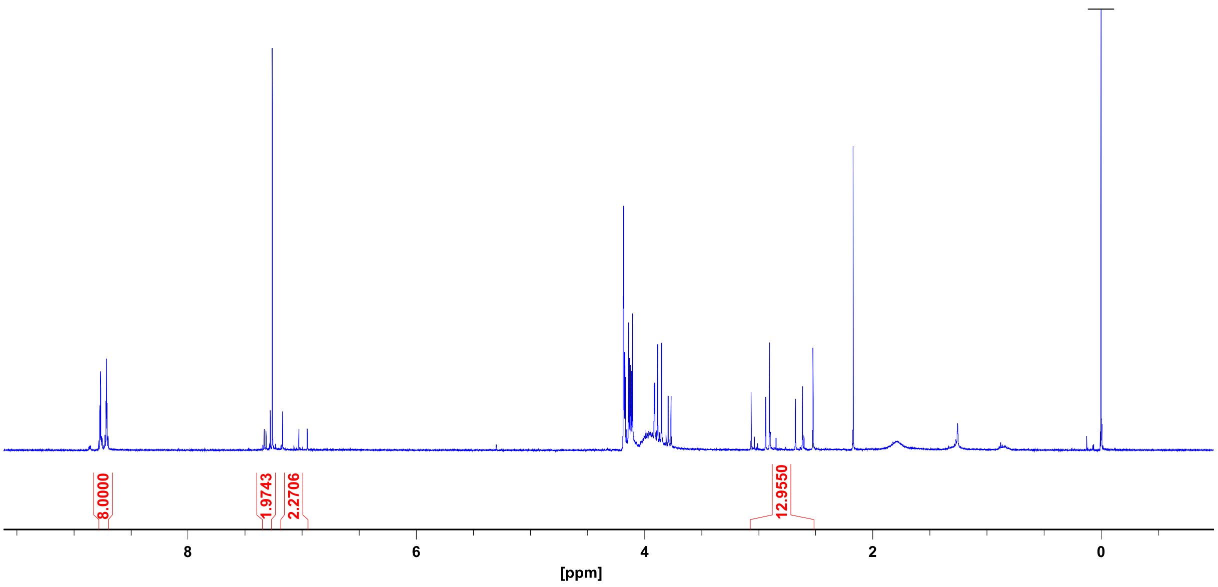

Figure S90: ^{1}H NMR spectrum of compound **29**, measured in $CDCl_3$ at 300 K.

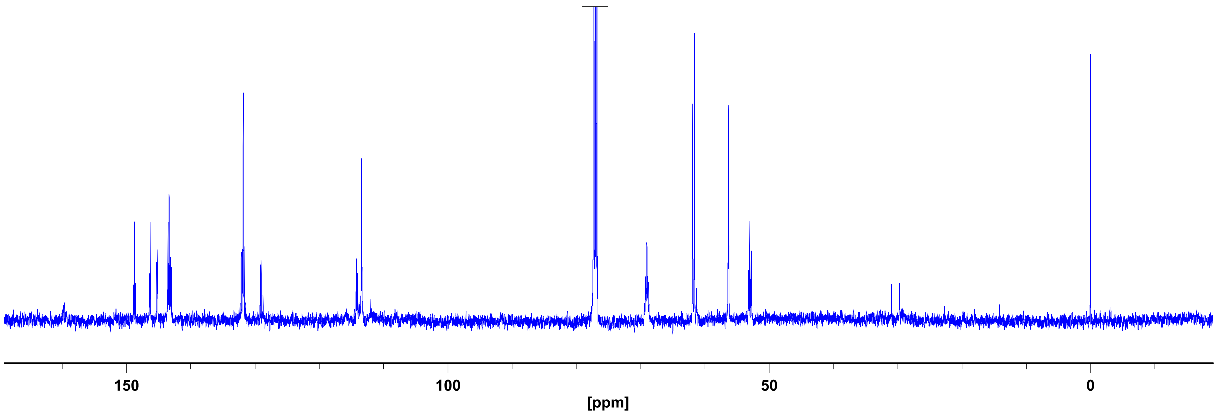

Figure S91: ^{13}C NMR spectrum of compound **29**, measured in $CDCl_3$ at 300 K.

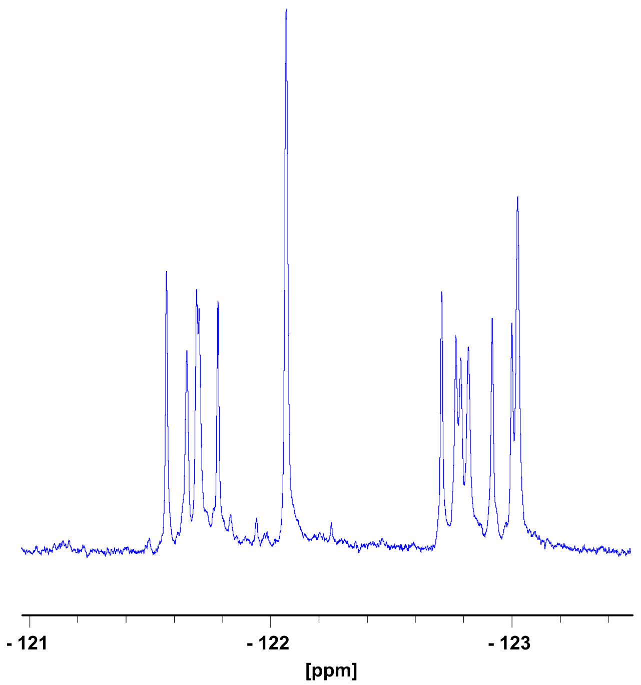

Figure S92: ^{19}F NMR spectrum of compound **29**, measured in $CDCl_3$ at 300 K.

5,10,15,20-Tetrakis-(2-(2,2,3,3,4,4,5,5-octafluoro-5-methoxycarbonylpent-1-yloxy)-3,4,5-trimethoxyphenyl)-nickel(II)porphyrin (30)

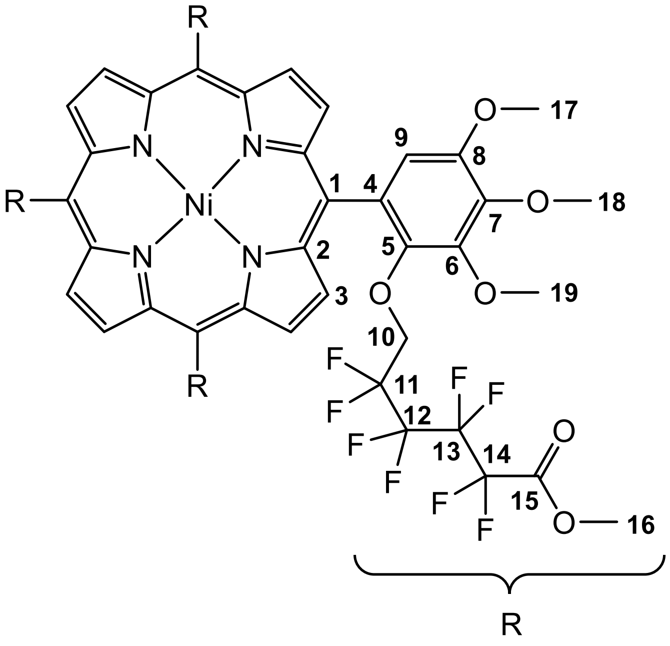

Yield: 97 %.

Melting Point: 86.7 °C.

^{1}H NMR (500 MHz, $CDCl_3$, 300 K): δ = 8.86-8.70 (m, 8 H, *H*-3), 7.22-7.09 (m, 4 H, *H*-9), 4.20-3.00 (m, 44 H, *H*-10, *H*-17, *H*-18, *H*-19), 3.56-3.00 (m, 12 H, *H*-16) ppm.

Due to atropisomers, an exact evaluation of the ^{1}H NMR spectrum was not possible and only the ranges of the signals were given. A qualitative evaluation of the ^{13}C NMR and ^{19}F NMR spectrum was not possible.

FT-IR: $\tilde{\nu}$ = 2925 (w), 2851 (w), 1780 (w), 1578 (w), 1490 (w), 1456 (w), 1434 (w),1413 (w), 1366 (m), 1323 (w), 1253 (w), 1183 (m), 1141 (m), 1109 (s), 1074 (m), 1039 (m), 1005 (m), 967 (w), 897 (w), 867 (w), 827 (w), 770 (w), 750 (w), 731 (w), 710 (w) cm^{-1}.

MS (MALDI-TOF, matrix: ClCCA): *m/z* (%) = 2182.2 (99).

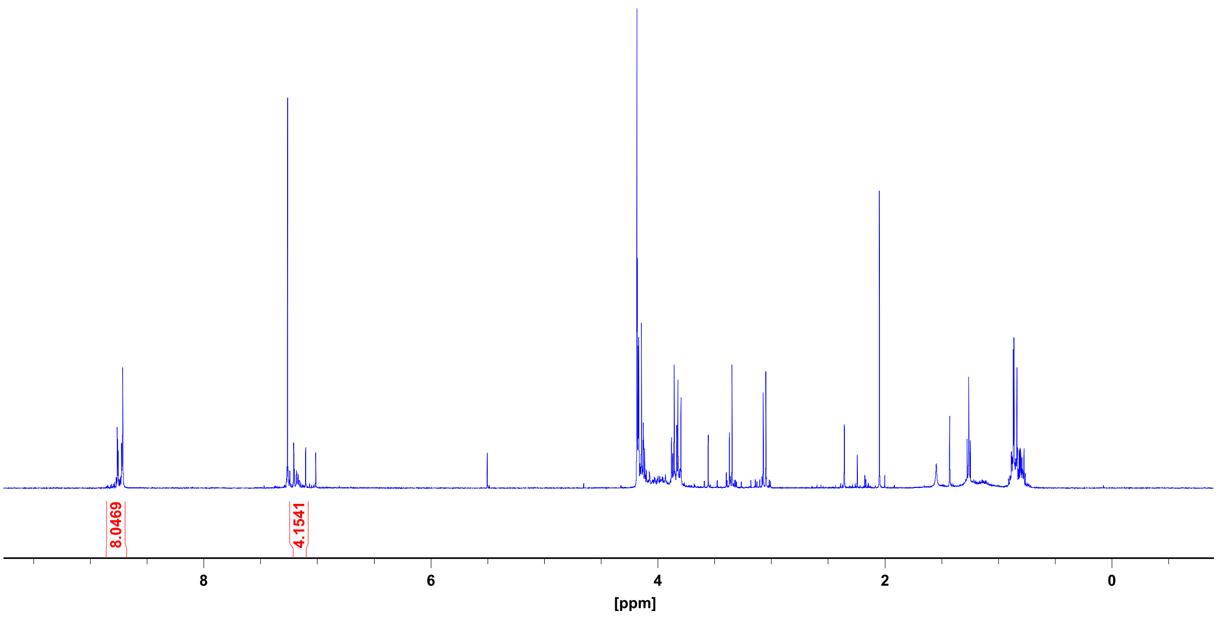

Figure S93: ^{1}H NMR spectrum of compound **30**, measured in $CDCl_3$ at 300 K.

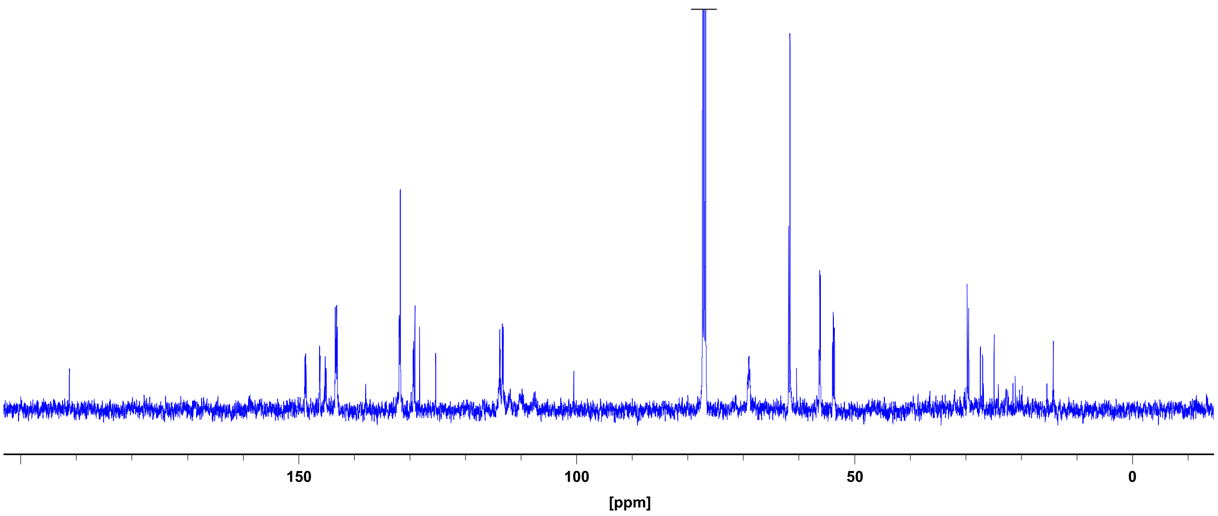

Figure S94: ^{13}C NMR spectrum of compound **30**, measured in $CDCl_3$ at 300 K.

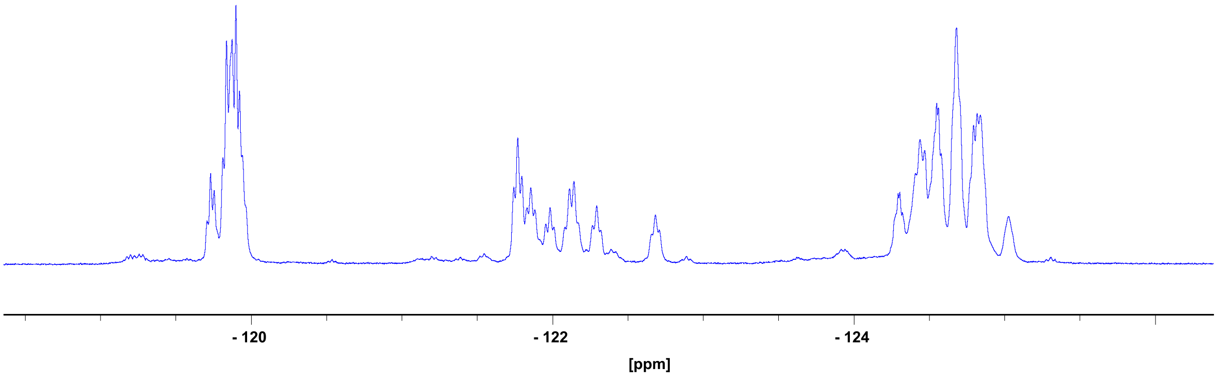

Figure S95: ^{19}F NMR spectrum of compound **30**, measured in $CDCl_3$ at 300 K.

5,10,15,20-Tetrakis-(2-(2,2,3,3,4,4,5,5,6,6,7,7-dodecafluoro-7-methoxycarbonyl-hept-1-yloxy)-3,4,5-trimethoxyphenyl)-nickel(II)porphyrin (31)

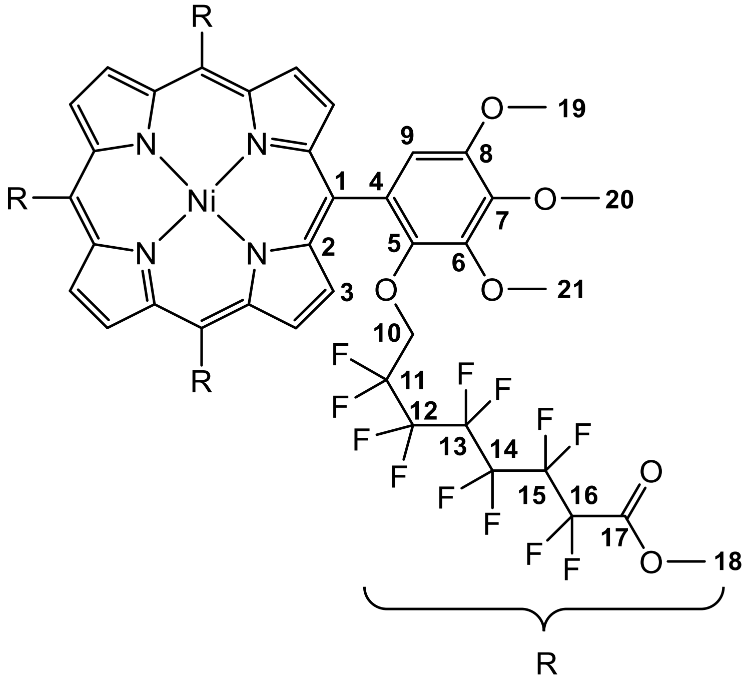

Yield: 57 %.

Melting Point: 63.2 °C.

^{1}H NMR (500 MHz, $CDCl_3$, 300 K, TMS): δ = 8.78-8.70 (m, 8 H, *H*-3), 7.23-7.04 (m, 4 H, *H*-9), 4.20-3.71 (m, 56 H, *H*-10, *H*-18, *H*-19, *H*-20, *H*-21) ppm.

Due to atropisomers, an exact evaluation of the ^{1}H NMR spectrum was not possible and only the ranges of the signals were given. A qualitative evaluation of the ^{13}C NMR and ^{19}F NMR spectrum was not possible.

FT-IR: $\tilde{\nu}$ = 2942 (w), 2852 (w), 1781 (w), 1578 (w), 1491 (w), 1456 (w), 1434 (w), 1414 (w), 1366 (w), 1322 (w), 1195 (m), 1139 (m), 1109 (m), 1075 (m), 1040 (w), 1006 (w), 968 (w), 899 (w), 826 (w), 803 (w), 765 (w), 715 (w), 697 (w), 655 (w), 613 (w) cm^{-1}.

MS (ESI): *m/z* (%) = 2582.29 (100) $[M]^+$.

MS (ESI, HR): $C_{92}H_{68}F_{48}N_4O_{24}Ni$ *m/z* = calc.: 2582.28050, found: 2582.28562, diff.: 1.98 ppm.

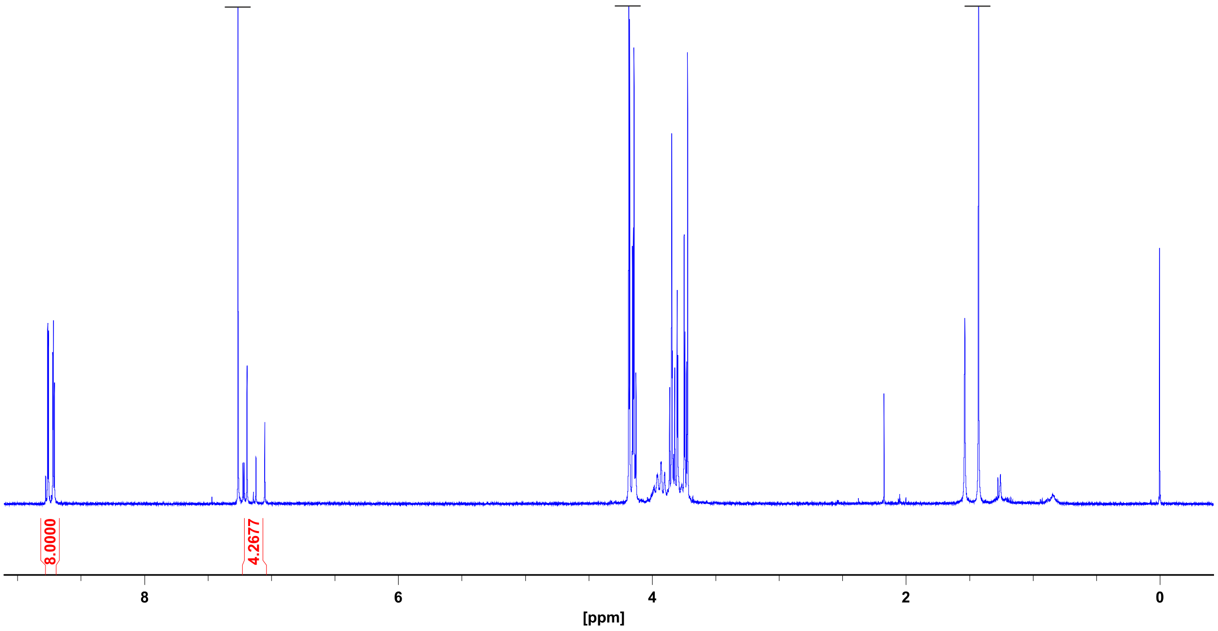

Figure S96: ^{1}H NMR spectrum of compound **31**, measured in $CDCl_3$ at 300 K.

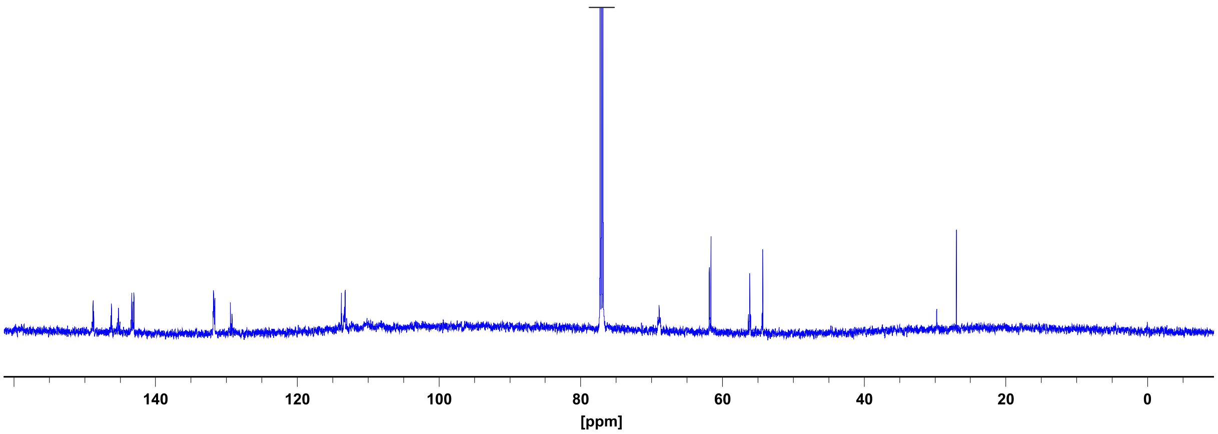

Figure S97: ^{13}C NMR spectrum of compound **31**, measured in $CDCl_3$ at 300 K.

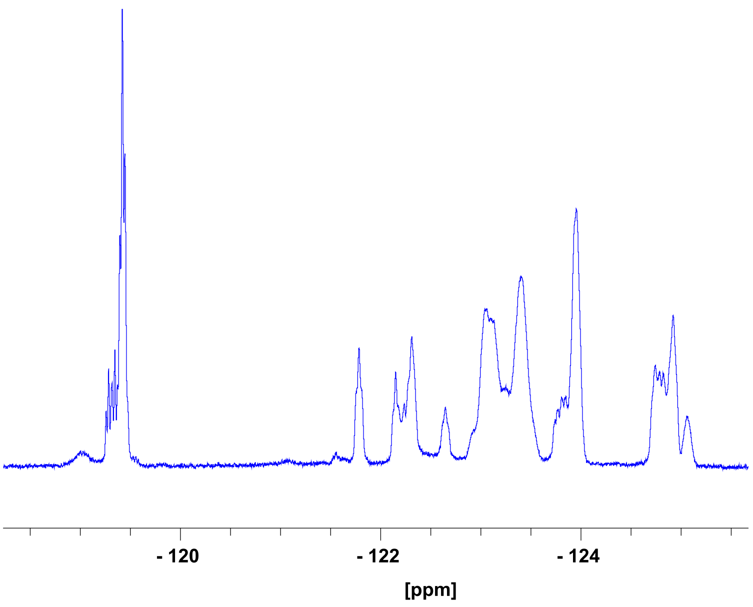

Figure S98: ^{19}F NMR spectrum of compound **31**, measured in $CDCl_3$ at 300 K.

3.11 General procedure of nickel porphyrins 32, 33 and 34 (free acid)

The corresponding nickel porphyrin **29–31** (1 equiv) was dissolved in methanol (40 mL) and lithium hydroxide (10 equiv) was added. This reaction mixture was stirred for 18 h at room temperature, then the solvent was removed in vacuo and water was added until all material had dissolved (30 mL). Afterwards 2 M HCl (20 mL) was added and a red solid precipitated and the aqueos phase turned colorless. The precipitate was filtered in vacuo and then dissolved in acetone (40 mL). After three cycles of freeze pump thaw (to remove HCl) the solvent was removed in vacuo to obtain a red solid.

5,10,15,20-Tetrakis-(2-(2,2,3,3-tetrafluoro-3-hydroxycarbonylprop-1-yloxy)-3,4,5-trimethoxyphenyl)-nickel(II)porphyrin (32)

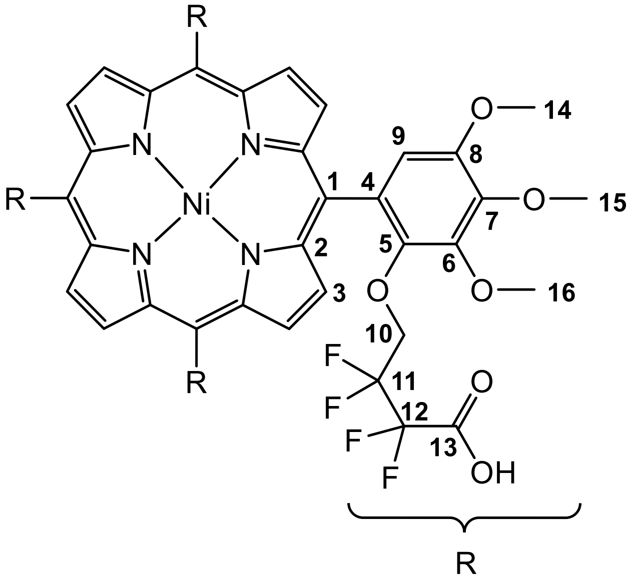

Yield: 94 %.

Melting Point: 127.5 °C.

^{1}H NMR (500 MHz, $CDCl_3$, 300 K, TMS): δ = 8.79-8.65 (m, 8 H, *H*-3), 7.47-6.96 (m, 4 H, *H*-9), 4.26-3.64 (m, 44 H, *H*-10, *H*-14, *H*-15, *H*-16), 3.59 (br. s, 4 H, O-*H*) ppm.

Due to atropisomers, an exact evaluation of the ^{1}H NMR spectrum was not possible and only the ranges of the signals were given. A qualitative evaluation of the ^{13}C NMR and ^{19}F NMR spectrum was not possible.

FT-IR: $\tilde{\nu}$ = 2921 (br, m), 2849 (w), 2041 (w), 1765 (br, m), 1578 (w), 1491 (m), 1458 (m), 1412 (m), 1365 (s), 1251 (m), 1226 (m), 1106 (s), 1075 (s), 1006 (s), 966 (m), 823 (m), 804 (m), 732 (m) cm^{-1}.

MS (ESI): *m/z* (%) = 431.17 (100) $[M]^{4-}$.

MS (ESI, HR): $C_{72}H_{56}F_{16}N_4O_{24}Ni$ *m/z* = calc.: 430.56011, found: 430.56032, diff.: 0.49 ppm.

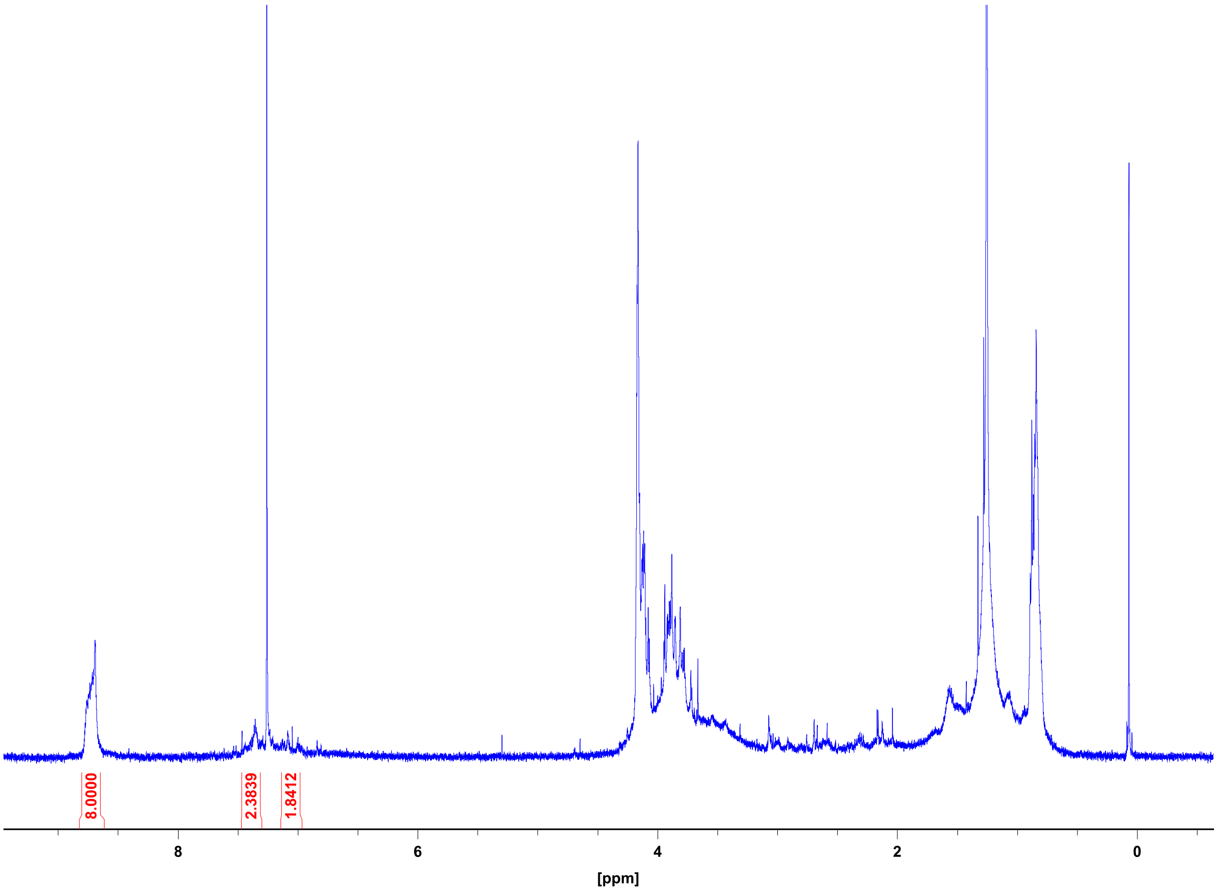

Figure S99: ^{1}H NMR spectrum of compound **32**, measured in $CDCl_3$ at 300 K.

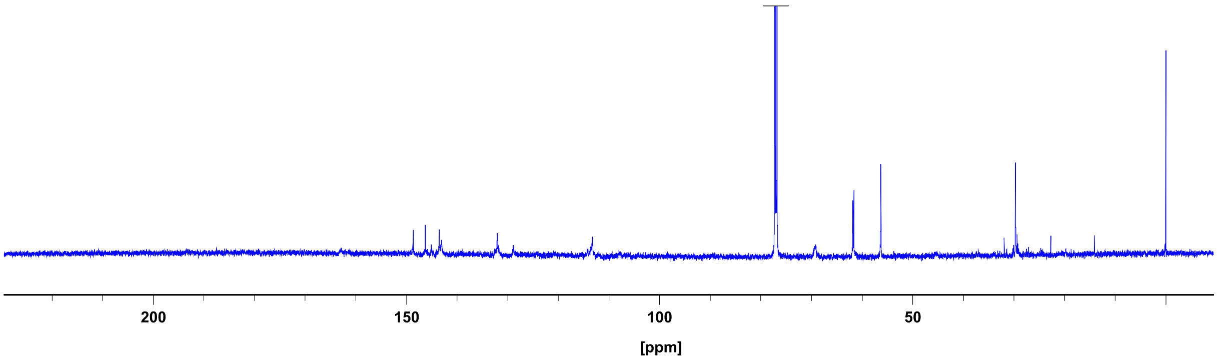

Figure S100: ^{13}C NMR spectrum of compound **32**, measured in $CDCl_3$ at 300 K.

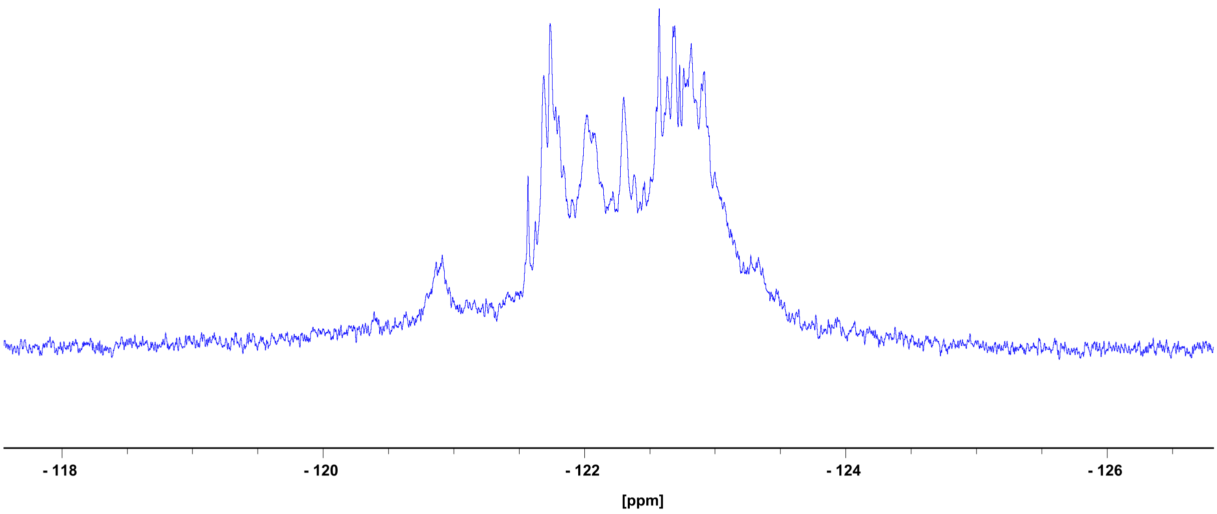

Figure S101: ^{19}F NMR spectrum of compound **32**, measured in $CDCl_3$ at 300 K.

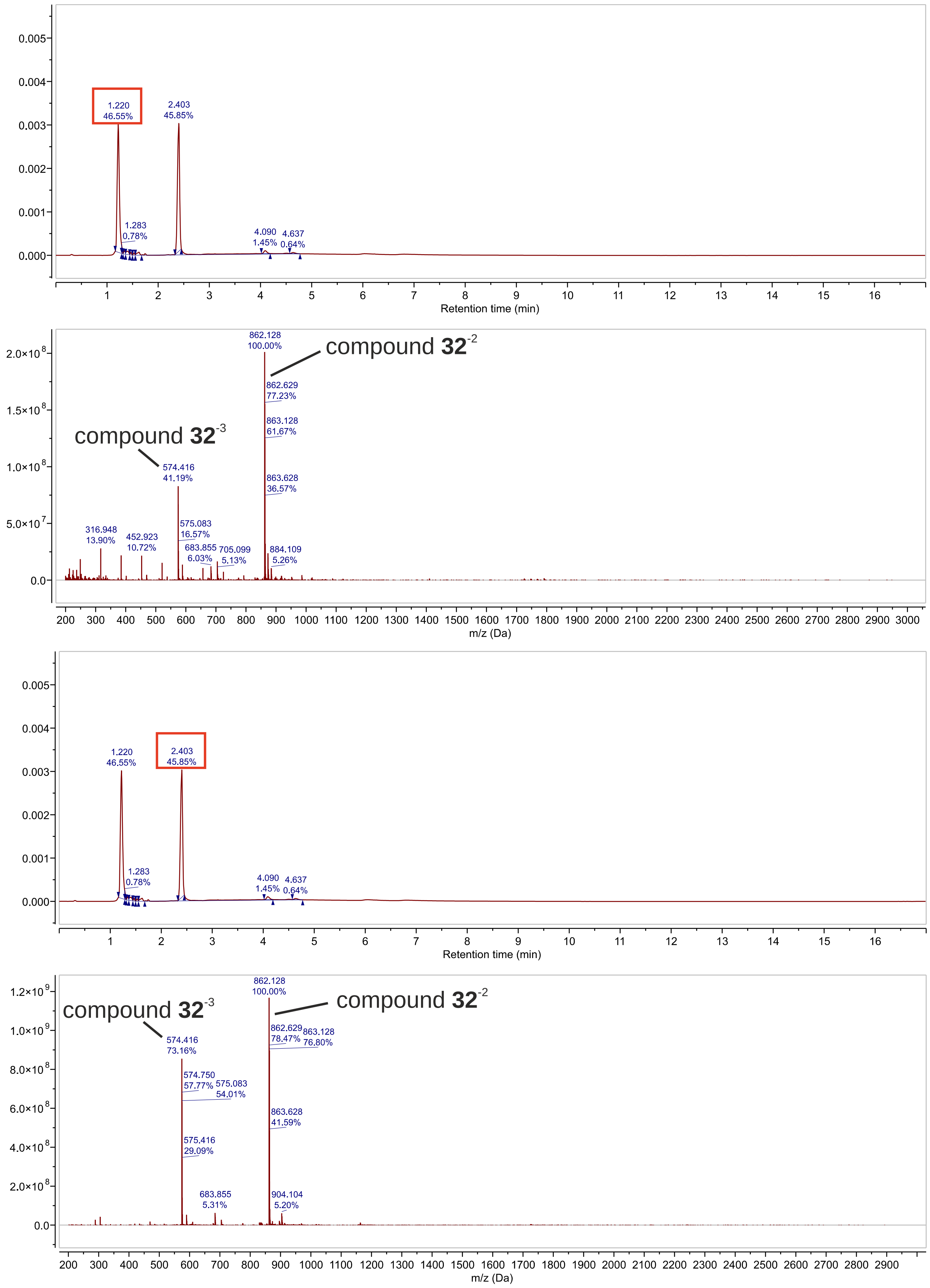

Figure S102: The product was separated by UHPLC. The HPLC was coupled with an ESI-MS. The separation was carried out at room temperature (22 °C). A linear gradient

of MeCN/H_2O (MeCN: 40%–80%) was used from 0–12 minutes. A linear gradient of MeCN/H_2O (MeCN: 80%–90%) was used from 12–17 minutes. Two main fractions were obtained with retention times of 1.220 min and 2.403 min. This indicates the separation of 2 atropisomers. In both fractions, the 2-fold and 3-fold negatively charged target product **32** could be identified.

HR masses could also be determined:

Retention time: 1.220 min:

MS (ESI, HR): $C_{72}H_{57}F_{16}N_4O_{24}Ni$ *m/z* = calc.: 574.41591, found: 574.41589, diff.: -0.02 ppm.

MS (ESI, HR): $C_{72}H_{58}F_{16}N_4O_{24}Ni$ *m/z* = calc.: 862.12750, found: 862.12769, diff.: 0.22 ppm.

Retention time: 2.403 min:

MS (ESI, HR): $C_{72}H_{57}F_{16}N_4O_{24}Ni$ *m/z* = calc.: 574.41591, found: 574.41589, diff.: -0.02 ppm.

MS (ESI, HR): $C_{72}H_{58}F_{16}N_4O_{24}Ni$ *m/z* = calc.: 862.12750, found: 862.12762, diff.: 0.15 ppm.

5,10,15,20-Tetrakis-(2-(2,2,3,3,4,4,5,5-octafluoro-5-hydroxycarbonylpent-1-yloxy)-3,4,5-trimethoxyphenyl)-nickel(II)porphyrin (33)

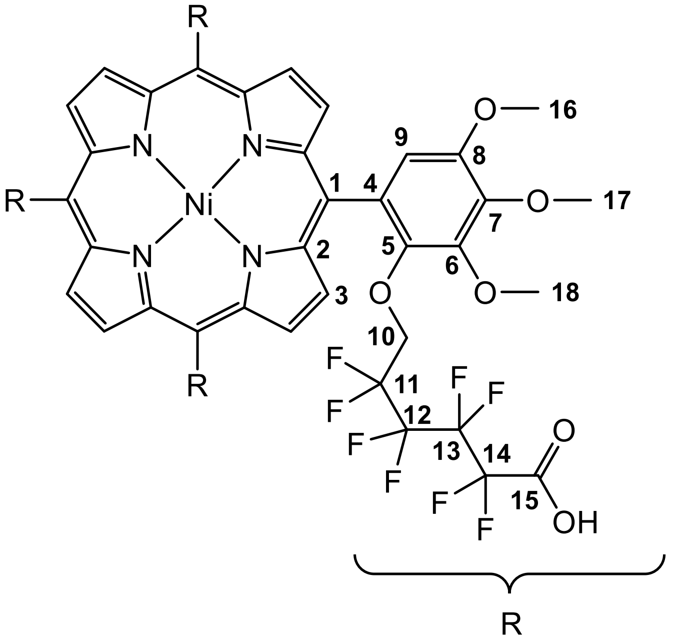

Yield: 39 %.

Melting Point: 86.4 °C.

^{1}H NMR (500 MHz, $CDCl_3$, 300 K, TMS): δ = 8.85-8.63 (m, 8 H, *H*-3), 7.61-6.65 (m, 4 H, *H*-9), 6.34 (s, 4 H, O-*H*), 4.24-3.05 (m, 44 H, *H*-10, *H*-16, *H*-17, *H*-18) ppm. Due to atropisomers, an exact evaluation of the ^{1}H NMR spectrum was not possible and only the ranges of the signals were given. A qualitative evaluation of the ^{13}C NMR and ^{19}F NMR spectrum was not possible.

FT-IR: $\tilde{\nu}$ = 2920 (w), 2850 (w), 2358 (w), 1772 (w), 1578 (w), 1489 (w), 1456 (w), 1433 (w), 1413 (w), 1365 (w), 1250 (w), 1179 (m), 1147 (w), 1108 (m), 1072 (m), 1034 (w), 1004 (w), 964 (w), 895 (w), 867 (w), 827 (w), 801 (w), 766 (w), 746 v 727 (w), 609 (w), 541 (w), 494 (w), 475 (w), 457 (w), 434 (w), 425 (w), 415 (w), 403 (w) cm^{-1}.

MS (ESI): *m/z* (%) = 530.55 (100) $[M]^{4-}$.

MS (ESI, HR): $C_{80}H_{56}F_{32}N_4O_{24}Ni$ *m/z* = calc.:530.55372, found: 530.55399, diff.: 0.51 ppm.

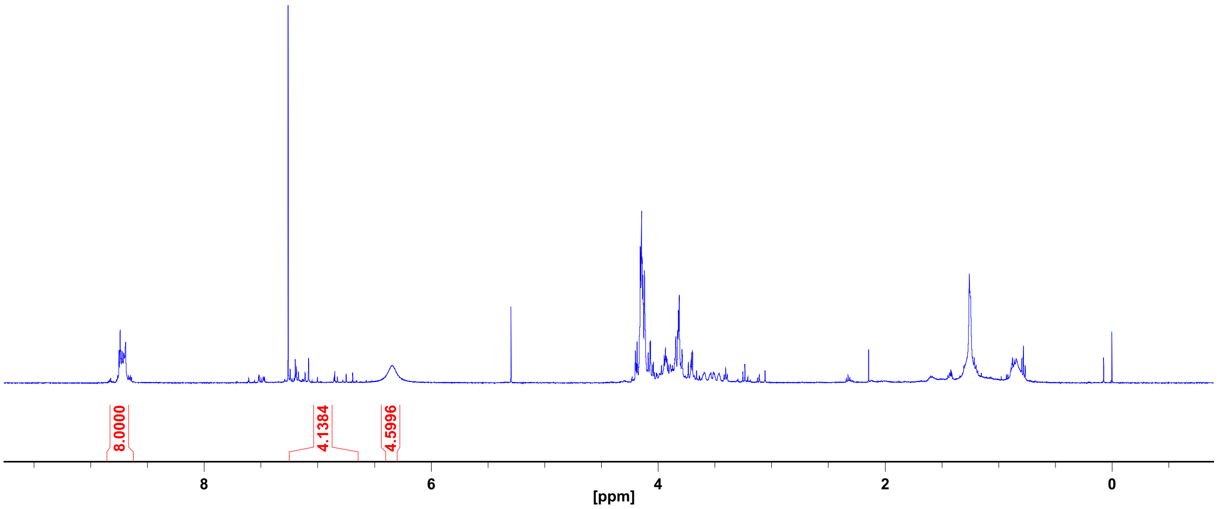

Figure S103: ^{1}H NMR spectrum of compound **33**, measured in $CDCl_3$ at 300 K.

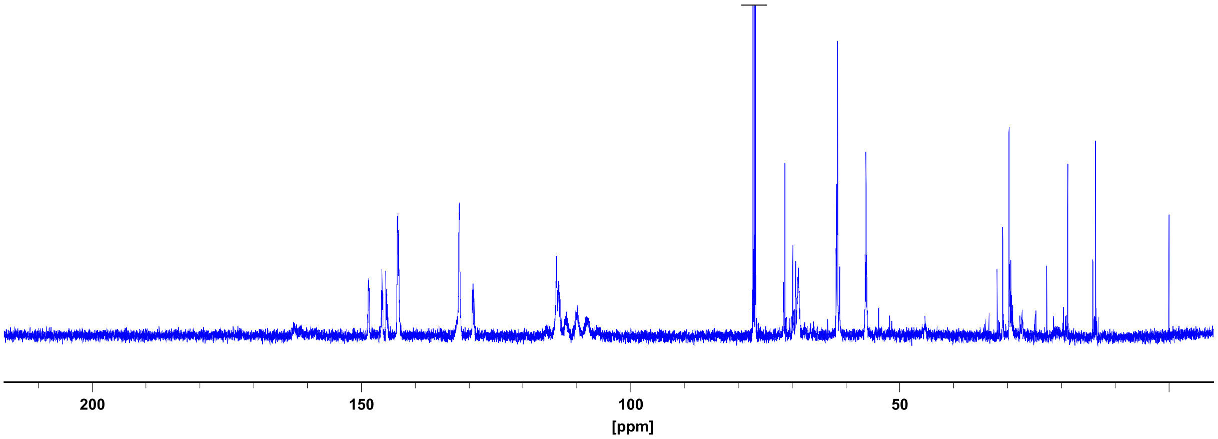

Figure S104: ^{13}C NMR spectrum of compound **33**, measured in $CDCl_3$ at 300 K.

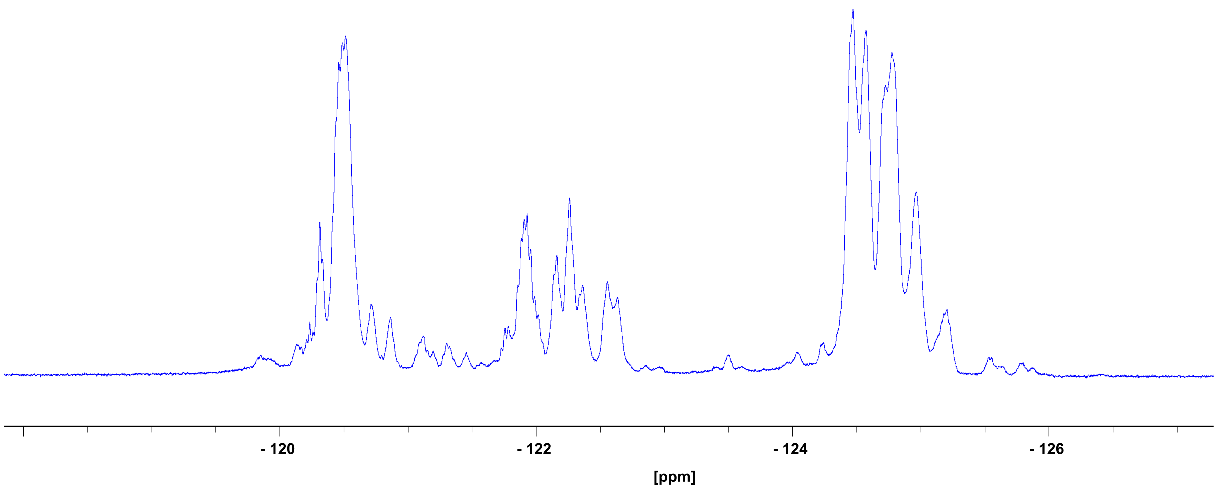

Figure S105: ^{19}F NMR spectrum of compound **33**, measured in $CDCl_3$ at 300 K.

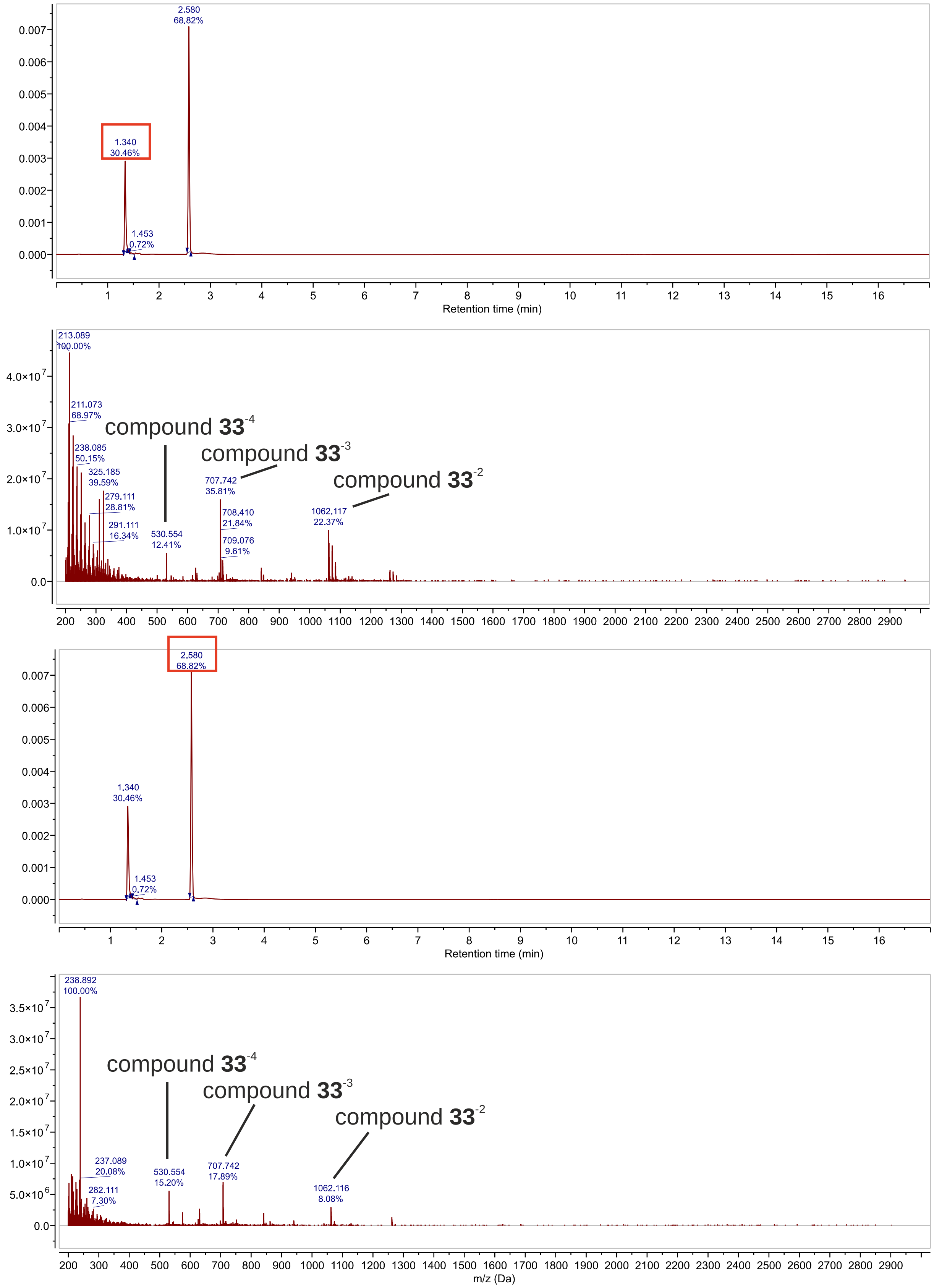

Figure S106: The product was separated by UHPLC. The HPLC was coupled with an ESI-MS. The separation was carried out at room temperature (22 °C). A linear gradient of MeCN/H_2O (MeCN: 60%–90%) was used from 0–12 minutes. A linear gradient of

MeCN/H_2O (MeCN: 90%–100%) was used from 12–17 minutes. Two main fractions were obtained with retention times of 1.340 min and 2.580 min. This indicates the separation of 2 atropisomers. In both fractions, the 2-fold, 3-fold and 4-fold negatively charged target product **33** could be identified.

HR masses could also be determined:

Retention time: 1.340 min:

MS (ESI, HR): $C_{80}H_{56}F_{32}N_4O_{24}Ni$ *m/z* = calc.:530.55372, found: 530.55431, diff.: 1.11 ppm.

MS (ESI, HR): $C_{80}H_{57}F_{32}N_4O_{24}Ni$ *m/z* = calc.:707.74072, found: 707.74181, diff.: 1.53 ppm.

MS (ESI, HR): $C_{80}H_{58}F_{32}N_4O_{24}Ni$ *m/z* = calc.:1062.11472, found: 1062.11649, diff.: 0.51 ppm.

Retention time: 2.580 min:

MS (ESI, HR): $C_{80}H_{56}F_{32}N_4O_{24}Ni$ *m/z* = calc.:530.55372, found: 530.55438, diff.: 1.24ppm.

MS (ESI, HR): $C_{80}H_{57}F_{32}N_4O_{24}Ni$ *m/z* = calc.:707.74072, found: 707.74182, diff.: 1.55 ppm.

MS (ESI, HR): $C_{80}H_{58}F_{32}N_4O_{24}Ni$ *m/z* = calc.:1062.11472, found: 1062.11646, diff.: 1.63 ppm.

5,10,15,20-Tetrakis-(2-(2,2,3,3,4,4,5,5,6,6,7,7-dodecafluoro-7-hydroxycarbonyl-hept-1-yloxy)-3,4,5-trimethoxyphenyl)-nickel(II)porphyrin (34)

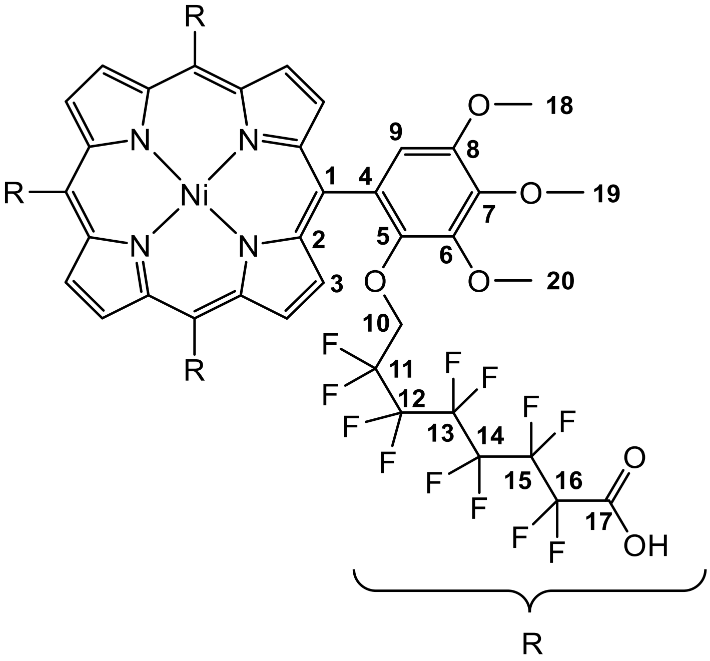

Yield: 45 %.

Melting Point: 112.4 °C.

^{1}H NMR (500 MHz, $CDCl_3$, 300 K, TMS): δ = 8.78-8.67 (m, 8 H, *H*-3), 7.47-7.04 (m, 4 H, *H*-9), 4.19-3.37 (m, 48 H, *H*-10, *H*-18, *H*-19, *H*-20, O-*H*) ppm.

Due to atropisomers, an exact evaluation of the ^{1}H NMR spectrum was not possible and only the ranges of the signals were given. A qualitative evaluation of the ^{13}C NMR and ^{19}F NMR spectrum was not possible.

FT-IR: $\tilde{\nu}$ = 2921 (w), 2850 (w), 1773 (w), 1578 (w), 1490 (w), 1458 (w), 1434 (w), 1414 (w), 1366 (w), 1275 (w), 1260 (w), 1196 (m), 1138 (m), 1108 (m), 1034 (w), 1005 (w), 965 (w), 897 (w), 828 (w), 801 (w), 764 (m), 749 (m), 707 (w), 653 (w), 536 (w), 494 (w), 480 (w), 443 (w), 434 (w), 419 (w) cm^{-1}.

MS (ESI): *m/z* (%) = 630.55 (100) [M]$^{4-}$.

MS (ESI, HR): $C_{88}H_{56}F_{48}N_4O_{24}Ni$ *m/z* = calc.:630.54734, found: 630.54785, diff.: 0.81.

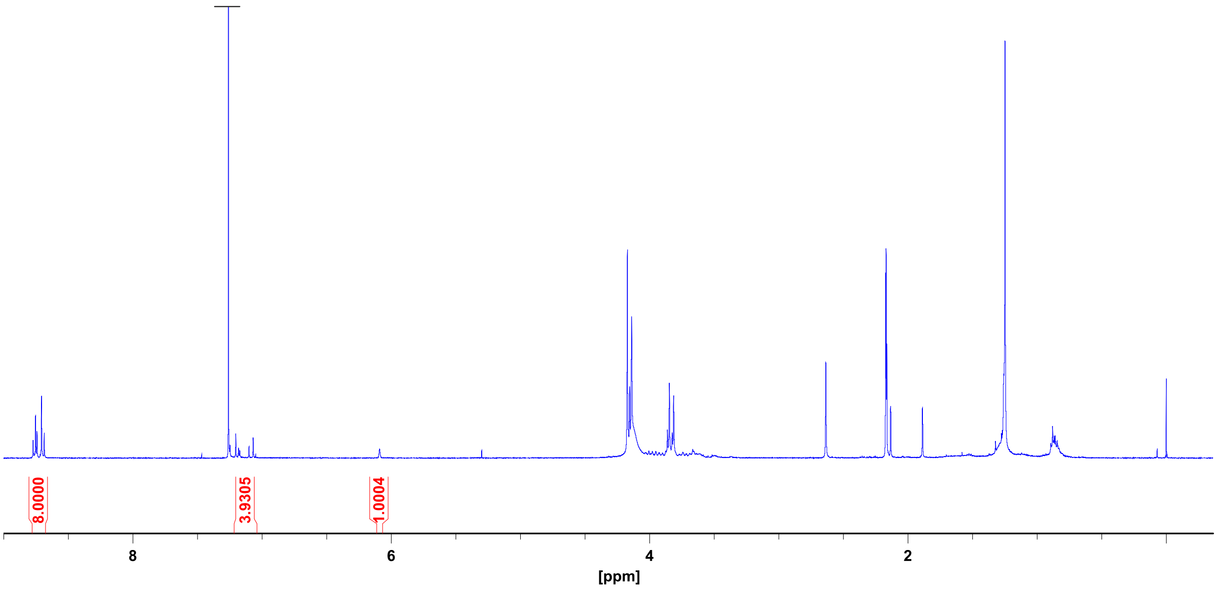

Figure S107: ^{1}H NMR spectrum of compound **34**, measured in $CDCl_3$ at 300 K.

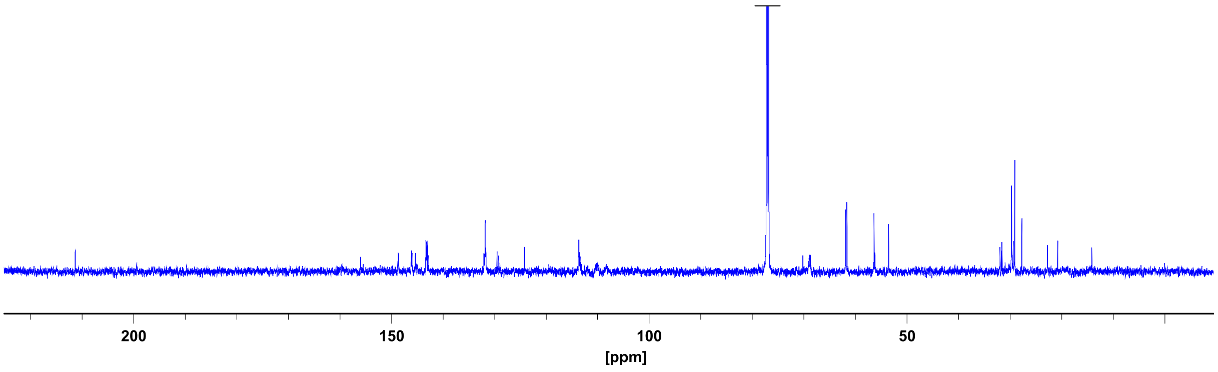

Figure S108: ^{13}C NMR spectrum of compound **34**, measured in $CDCl_3$ at 300 K.

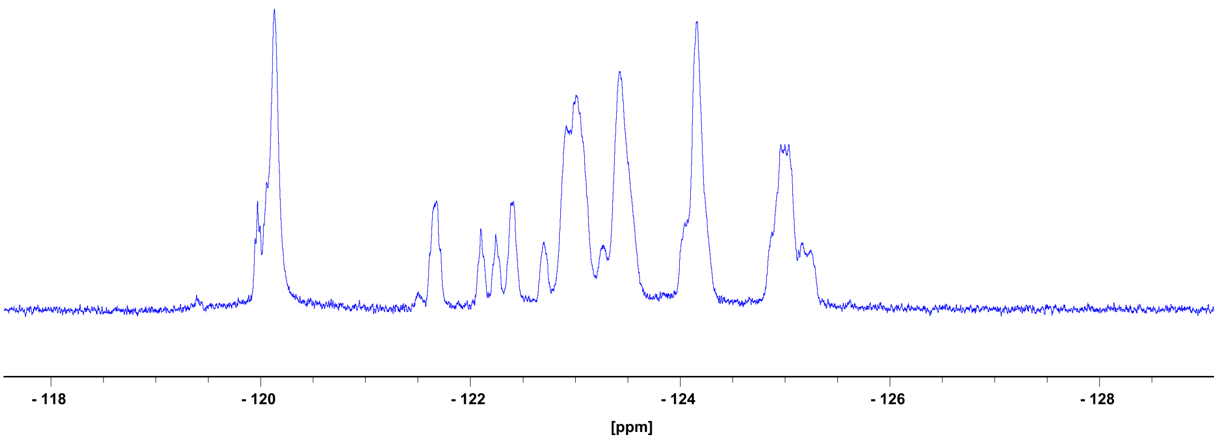

Figure S109: ^{19}F NMR spectrum of compound **34**, measured in $CDCl_3$ at 300 K.

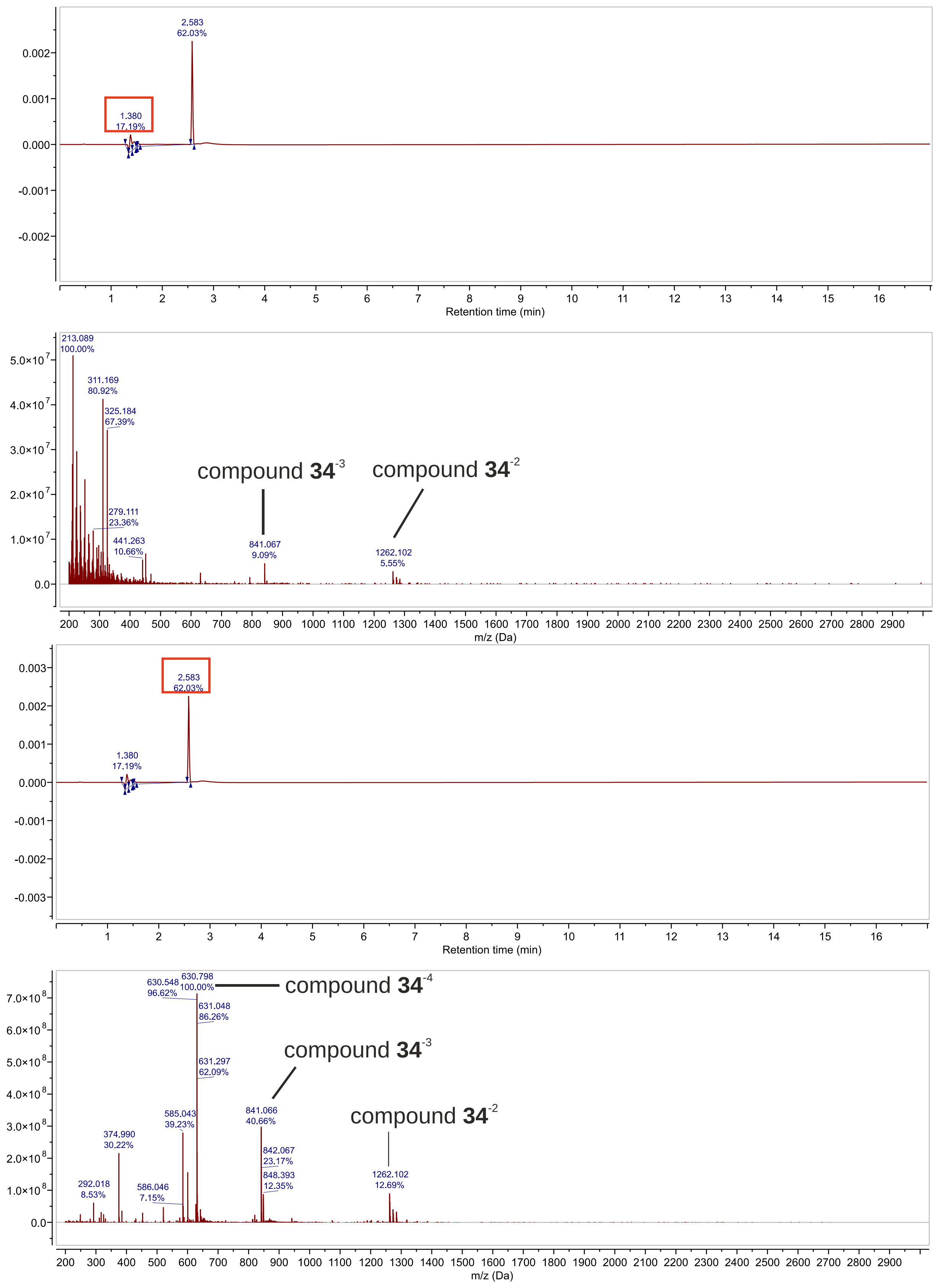

Figure S110: The product was separated by UHPLC. The HPLC was coupled with an ESI-MS. The separation was carried out at room temperature (22 °C). A linear gradient

of MeCN/H_2O (MeCN: 60%–90%) was used from 0–12 minutes. A linear gradient of MeCN/H_2O (MeCN: 90%–100%) was used from 12–17 minutes. Two main fractions were obtained with retention times of 1.380 min and 2.583 min. This indicates the separation of 2 atropisomers of which the one with the shorter retention time is obviously formed as a minor product. In the first fraction the 2-fold and 3-fold negatively charged target product **34** could be identified. In the second fraction the 2-fold, 3-fold and 4-fold negatively charged target product **34** could be identified.

HR masses could also be determined:

Retention time: 1.380 min:

MS (ESI, HR): $C_{88}H_{57}F_{48}N_4O_{24}Ni$ *m/z* = calc.: 841.06554, found: 841.06653, diff.: 1.18 ppm.

MS (ESI, HR): $C_{88}H_{58}F_{48}N_4O_{24}Ni$ *m/z* = calc.: 1262.10195, found: 1262.10181, diff.: -0.11 ppm.

Retention time: 2.583 min:

MS (ESI, HR): $C_{88}H_{56}F_{48}N_4O_{24}Ni$ *m/z* = calc.:630.54734, found: 630.54797, diff.: 1.01 ppm.

MS (ESI, HR): $C_{88}H_{57}F_{48}N_4O_{24}Ni$ *m/z* = calc.:841.06554, found: 841.06610, diff.: 0.67 ppm.

MS (ESI, HR): $C_{88}H_{58}F_{48}N_4O_{24}Ni$ *m/z* = calc.:1262.10195, found: 1262.10193, diff.: -0.02 ppm.

References

(1) Gao, W.; Li, Q.; Chen, J.; Wang, Z.; Hua, C. *Molecules* **2013**, *18* (12), 15613–15623. doi: 10.3390/molecules181215613.
